# Temporary bilateral clamping of renal arteries induces ischemia–reperfusion: A new pig model of acute kidney injury using total intravenous anesthesia

**DOI:** 10.14814/phy2.70203

**Published:** 2025-02-02

**Authors:** Axel Guilpin, Mathieu Magnin, Axel Aigle, Jean‐Yves Ayoub, Timothée Schuhler, Romain Lac, Thierry Marchal, Thomas Brichart, Abdessalem Hammed, Vanessa Louzier

**Affiliations:** ^1^ MexBrain Villeurbanne France; ^2^ Université de Lyon, UR APCSe Agressions Pulmonaires et Circulatoires Dans le Sepsis, VetAgro Sup Marcy l'Etoile France; ^3^ Université de Lyon, VetAgro Sup, Unité de Physiologie, Pharmacodynamie et Thérapeutique Marcy l'Etoile France; ^4^ Université de Lyon, VetAgro Sup, Pole de Pathologie Vétérinaire Marcy l'Etoile France

**Keywords:** Acute kidney injury (AKI), ischemia‐reperfusion, porcine model, preclinical model, renal ischemia, total intravenous anesthesia

## Abstract

Ischemia‐reperfusion (IR) is a leading cause of acute kidney injury (AKI), and pigs are commonly used in preclinical AKI models. However, existing models often vary in the methods used to induce ischemia, and the resulting AKI tends to be mild‐to‐moderate. Moreover, follow‐up is often performed under volatile anesthesia, which, in contrast to total intravenous anesthesia (TIVA), can induce malignant hyperthermia and cause hemodynamic instability. Here we present a novel surgical model of IR‐induced AKI using bilateral renal artery clamping under TIVA. Anesthesia was induced via TIVA with diazepam, ketamine, and morphine. After retroperitoneal exposure, the renal arteries were isolated and clamped with a plastic tube for 90 min, followed by 8 h of reperfusion. The IR group (*n* = 6) was compared with a Sham group (*n* = 5) that underwent the same procedure without IR. The IR group developed moderate‐to‐severe AKI as evidenced by reduced glomerular filtration, a 158% increase in plasma creatinine versus 21% in the Sham group, and elevated neutrophil gelatinase‐associated lipocalin levels (+280% in IR vs. 0% in Sham), indicating tubular injury. Histopathology confirmed these findings. Thus, this preclinical model successfully induced moderate‐to‐severe AKI in pigs. The TIVA anesthetic protocol offered several advantages compared to halogenated gas anesthesia.

## 1 INTRODUCTION

Acute kidney injury (AKI) is a severe syndrome characterized by impaired renal function, defined by a decrease in glomerular filtration rate (GFR), which leads to the retention of nitrogenous waste (Siew & Davenport, 2015). AKI represents a significant public health problem, affecting 5 to 20% of hospitalized patients and up to 50% of those in intensive care units (Ronco et al., 2019; Singbartl & Kellum, 2012). AKI has varying stages of severity which are classified according to the “Kidney Disease: Improving Global Outcome (KDIGO)” guidelines (Khwaja, 2012). Effective medical management, tailored to the stage of AKI, is crucial to prevent the progression to chronic kidney disease.

A major cause of AKI is ischemia–reperfusion (IR) syndrome, commonly observed after cardiopulmonary bypass (CPB). The incidence of AKI after CPB can reach 42% and is associated with a higher mortality rate (Hobson et al., 2009; Wang et al., 2017). In 80% of cases of AKI observed during resuscitation, acute tubular necrosis (ATN) is the underlying cause, which results from ischemic damage on the tubules (Ducheyron et al., 2008).

Preclinical models of IR‐induced AKI have been established in rodents (Hesketh et al., 2014; Huang et al., 2015; Liu et al., 2016; Wei & Dong, 2012). However, mouse models have limitations due to anatomical differences from humans, especially in the context of renal diseases. In contrast, pigs are more anatomically similar to humans particularly in terms of renal architecture and physiological function (Huang et al., 2021; Packialakshmi et al., 2020; Parekh et al., 2013; Pereira‐Sampaio et al., 2004). Additionally, their larger size facilitates repeated blood sampling, which is more challenging in rodent models.

Various techniques are used to induce AKI by IR (Huang et al., 2021). Clamping of the entire renal vascular pedicle (veins and arteries), or clamping just the veins, results in more severe IR than clamping the arteries alone (Huang et al., 2022; Orvieto et al., 2007; Owji et al., 2018; Park et al., 2008; Silberstein et al., 2013). Venous clamping can lead to an increase in capillary blood pressure, causing congestion in the kidneys, which in turn amplifies oxidative stress; reactive oxygen species may accumulate in the kidneys, leading to increased damage (Owji et al., 2018). However, arterial clamping more accurately replicates AKI secondary to CPB bypass in humans (Li et al., 2012; Mitty et al., 1996; Thadhani et al., 1996). Unilateral clamping allows for direct comparison between both kidneys, but it carries the risk of compensatory kidney function, whereas bilateral clamping eliminates this risk. Fu et al. reported that bilateral IR more closely mirrors the human physiopathology although careful selection of ischemia duration is necessary to avoid irreversible damage (Fu et al., 2018).

The duration of ischemia to induce experimental acute kidney injury (AKI) in the literature is highly variable, and there is no consensus on the choice of time. Huang et al. state, in a review, that this duration is generally between 30 min and 2 h (Huang et al., 2021). A period of ischemia of less than 30 min has been shown not to induce significant lesions. Conversely, ischemia lasting 2 h or more causes irreversible damage (Bechara et al., 2016; Horikawa et al., 2022; Huang et al., 2021; Owji et al., 2018). Concerning unilateral clamping, Laven et al. suggested that ischemia of up to 90 min can result in full recovery, and Horikawa et al. showed that ischemia of 60 min did not result in measurable lesions whereas ischemia duration of 120 min was associated with severe AKI that persist 7 days after surgery (Horikawa et al., 2022; Laven et al., 2004). Gardner et al. established a porcine model of mild‐to‐moderate AKI by bilateral cross‐clamping of kidney arteries for 40 min (Gardner et al., 2014).

Pigs are prone to malignant hyperthermia (MH), a genetic condition that is particularly triggered by anesthetic halogenated gases (Musk, 2015; Pehböck et al., 2015). Although halogenated gases are commonly used for pig preclinical models, strict control of the pig's genetic profile is necessary during long‐term anesthesia to prevent MH (Gardner et al., 2014). Furthermore, the use of halogenous could be associated with hypotension, which can compromise kidney function. Maintaining stable hemodynamics is especially important in preclinical models of renal disease (Ebert et al., 1995; Lehman et al., 2010).

The aim of our study was to describe a model of IR‐induced AKI using surgical bilateral clamping of the renal arteries for 90 min. We hypothesized that this procedure would lead to moderate to severe AKI. The model was implemented on anesthetized pigs using a total intravenous anesthesia (TIVA) protocol to minimize the risk of malignant hyperthermia (MH).

## 2 MATERIALS AND METHODS

### 2.1 Ethics statement

This study was conducted in accordance with the Guide for the Care and Use of Laboratory Animals, and all procedures performed on animals were approved by the Ethics Committee of our institution (VetAgroSup, Marcy l'Etoile, France, authorization number: 2146). All procedures adhered to the guidelines set forth by Directive 2010/63/EU of the European Parliament on the protection of animals used for scientific purposes. This article was written following the ARRIVE 2.0 guidelines.

### 2.2 Animals and sample size calculation

In this study, two groups of pigs were compared. The “IR group” consisted of animals that underwent bilateral renal ischemia–reperfusion (IR) while the “Sham group” underwent identical procedures, excluding the IR procedure. A total of 11 healthy piglets (Youna, *Sus scrofa domesticus*), including seven males and four females, were used. The pigs were randomly assigned to one of these two groups.

The pigs were 3 months old with a median weight of 38 kg (ranging from 35 to 45 kg). The sample size was calculated to demonstrate AKI in the group undergoing renal IR. Specifically, we aimed to observe a plasma creatinine concentration 1.5 times higher in this group compared to the “Sham group”. In our laboratory, the average creatinine for pigs of the same age is 143 mmol/L (±38). To calculate the sample size, we set the effect size at 71 mmol/L, the standard deviation at 38 mmol/L, the significance level at 0.05, and the power at 0.9. The calculation indicated that five pigs per group would be required. To account for potential losses, one additional pig was added to the IR group. The power *t*‐test function of R software was used for this calculation. The “IR” group consisted of 6 pigs (2 females and 4 males), while the “Sham group” consisted of 5 pigs (2 females and 3 males).

### 2.3 Refinement

Pigs underwent a one‐week acclimation period, receiving a standard diet twice daily (with extra food hidden in toys) and unrestricted access to water. Enrichment included toys and hanging chains, with daily visits to promote acclimatization and regular cage cleaning for hygiene. Housing conditions were maintained at 22°C (±1°C) and 55% (±5%) humidity.

### 2.4 Study design

**FIGURE 1:**
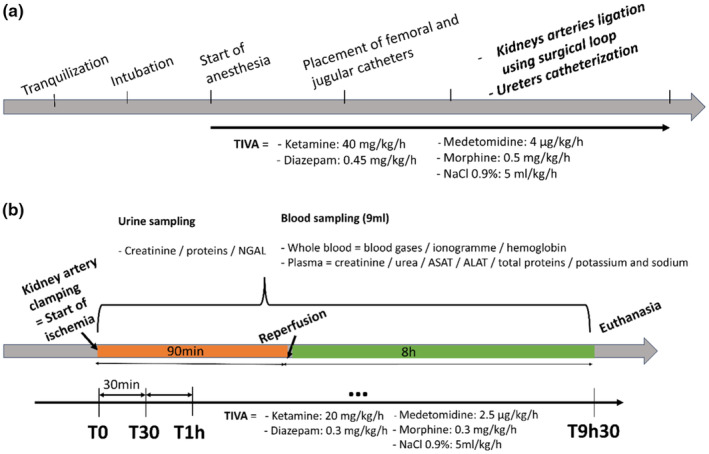
Study design. (a) Animal preparation and surgical procedures. (b) IR establishment and follow‐up. Samples were collected every 30 min (all the procedure lasted 9 h 30 composed by 90 min of ischemia (orange) followed by 8 h of reperfusion (green)). The black arrow represents time passed under total intravenous anesthesia (TIVA).

The design of this study was described in Figure 1. Figure 1a depicts animal preparation, from tranquilization to the surgical act. Figure 1b resume IR establishment and follow‐up, when blood and urine samples were collected. Total intravenous anesthesia (TIVA) was described with black arrow.

### 2.5 Anesthesia

Premedication was administered using a combination of tiletamine and zolazepam (Zoletil® 100, Virbac, France) at a dose of 6 mg/kg (IM). A peripheral venous catheter was then inserted into the auricular vein. For anesthesia induction, pigs received an intravenous (IV) bolus of ketamine (5 mg/kg), diazepam (0.5 mg/kg), and medetomidine (2.5 μg/kg). was maintained using a total intravenous anesthesia (TIVA) protocol with continuous infusions of ketamine (20 mg/kg/h—Ketamidor® 100 mg/mL, Axience, France), medetomidine (2.5 μg/kg/h—Medetor® 1 mg/mL, F1 10 mL, Virbac, France), and diazepam (0.3 mg/kg/h—Diazedor® 5 mg/mL, Axience, France). For analgesia, pigs received morphine (MCHL 10 mg/mL, ABT 10 mL, Aguettant, France), with an initial IV bolus of 0.3 mg/kg followed by a continuous infusion at 0.3 mg/kg/h IV. During the surgical procedures, the infusion rate of anesthetics and analgesics were increased to 40 mg/kg/h for ketamine, 0.45 mg/kg/h for diazepam, 4 μg/kg/h for medetomidine and 0.5 mg/kg/h for morphine. After induction, the pigs were placed into prone position for endotracheal intubation using a 7.5–8 mm‐diameter tube. Once intubated, the animals were placed on controlled mechanical ventilation with an initial tidal volume of 8.0 mL/kg and a respiratory rate of 16 breaths/min, which were subsequently adjusted to maintain end‐tidal carbon dioxide between 35 and 45 mmHg (4.5–6 kPa). Intravenous fluids (NaCl 0.9%) were administered at a rate of 5 mL/kg/h. When glycemia dropped below 3 mmol/L, glucose 5% was administered intravenously along with saline perfusion. The glucose and saline solutions were combined in a ratio of 1/3 of glucose 5% and 2/3 of saline (0.9%). Noradrenaline (Noradrenaline Tartrate 2 mg/mL, Aguettant, France) was administered intravenously if the mean arterial pressure (MAP) dropped below 55 mmHg, starting at a dose of 0.05 μg/kg/min. The noradrenaline dose was adjusted as needed to maintain MAP above 60 mmHg.

### 2.6 Surgical procedures

#### 2.6.1 Blood vessel catheterization

After intubation, pigs were put into supine position, and then, a venous and an arterial catheter were placed using the Seldinger's method. The modified Seldinger technique involved inserting a needle directly into the arterial lumen without piercing the posterior wall, passing a guidewire through the needle into the vessel lumen and introducing the vascular catheter over the guidewire. The venous catheter (double‐lumen, 7 French, SA7FD15 ADVETIS) was placed in the right jugular vein and was used for drug perfusion and fluid delivery. The arterial catheter (single‐lumen, 4 French, 5115.112 Vygon) was inserted in the right femoral artery and was used for blood sampling and arterial pressure's measurement. Both catheters were sutured to the skin.

#### 2.6.2 Renal arteries clamping

After catheterization, pigs were placed in lateral decubitus position and in a hyperextension position, and the operative area was disinfected with antiseptic solutions (povidone‐iodine or chlorhexidine). To open up the space between the abdomen and the costal margin, a cushion was placed under the animal so that the flank just behind the ribs is flexed 20–30 degrees in an inverted‐V position. The first incision was made below the last rib to create a retroperitoneal space.

Incision: A 45° incision (approximately 4–6 cm) was made to the caudoventral direction from the junction of the ribs and the spinous processes of the vertebrae.

After skin incision, the muscular layers of the lateral abdominal wall were dilacerated. This includes the thoracolumbar fascia, the external oblique, the internal oblique, and the transversus abdominis muscles.

The retroperitoneal space was penetrated dorsolaterally to avoid tearing the parietal peritoneum. The kidney was visualized, along with the ureter, renal vein, and renal artery (Figure S1a), before isolating the renal artery (Figure S1b). A useful landmark was the renal lymph node, as the renal artery was located right next to this lymph node. A surgical loop (B1095633 BBRAUN) was placed around the previously isolated renal artery, and the two ends of the surgical loop were inserted into a plastic infusion tubing that extended from the animal's flanks. The muscular planes were then closed, taking care to leave the tube through which the lace passed. The skin was sutured, and a watertight dressing was applied. Animals were turned over, and the same procedure was performed on the other kidney. Pigs were then placed supine to catheterize the ureters for urine collection. This position also enabled renal ischemia to be performed on both kidneys at the same time.

#### 2.6.3 Ureters catheterization

The pigs were placed in the dorsal recumbent position, and the operative area was disinfected with antiseptic solutions (povidone‐iodine or chlorhexidine). A laparotomy was performed along the midline, with an incision of the abdominal wall from the pubis to the umbilicus. The bladder was visualized, emptied with an 18G needle, and then retracted to visualize the openings of the ureters. Once the ureters were identified, silicone Foley catheters (CH8) were inserted into each ureter. The catheters were secured in place by carefully ligating around the ureter with absorbable sutures (2/0 metric 3). The balloon of the Foley catheters was inflated with sterile saline to ensure the integrity of the system and prevent any urinary reflux. The muscle layers and skin were sutured, allowing the catheters to exit through a discreet opening to reduce the risk of tension on the tubes. The catheters were connected to separate sterile containers (one for each kidney), enabling continuous monitoring of diuresis for each kidney.

This procedure was applied for both “IR” group and “Sham” group.

### 2.7 Ischemia induction and reperfusion

Prior to clamping the renal arteries, a 30‐min period was conducted to assess baseline diuresis (T0). Heparin was administered intravenously at a dose of 50 UI/kg to reduce the risk of thrombosis in renal blood vessels.

Ischemia was performed on both kidneys at the same time by pushing the plastic tube while pulling the surgical loop. The renal artery was thus compressed by the plastic tube, and blood no longer flowed through it. A hemostatic clamp held the system under tension, enabling ischemia to be performed for 90 min.

The success of the ischemia was confirmed by the cessation of urine production. Thereafter, the loops were released, and the kidneys were reperfused. Reperfusion lasted 8 h, and the pigs were monitored throughout the procedure (time periods: from T1h30 to T9h30; see Figure 1).

The Sham pigs did not experience a period of ischemia. For pigs in this group, surgical loops were removed immediately after the 30‐min T0 period and monitored for 8 h of reperfusion (see Figure 1).

### 2.8 Anesthesia monitoring

A pressure sensor was connected to heparinized physiological solution to ensure catheter permeability. Oxygen saturation (SpO_2_), expired CO_2_ (EtCO_2_), heart rate (HR), rectal temperature, and mean arterial pressure (MAP) were recorded every 30 min during blood and urine sampling. The monitoring of these parameters was performed using the Monitor CARESCAPE B650 (GE Healthcare, Little Chalmont, United Kingdom).

### 2.9 Blood sample and analysis

Arterial blood samples (9 mL) were collected every 30 min from T0 in both “IR” and “Sham” groups, resulting in 20 and 17 samples, respectively (Figure 1). Samples were collected in lithium‐heparin tubes for blood gas analysis which was performed using the Stat Profile Prime® Vet (Nova Medical, Waltham, United States), according to the manufacturer's instructions. Blood potassium, sodium, and hemoglobin levels were measured using the direct ion‐selective electrode method or colorimetric method. After centrifugation, plasma was separated and used to measure creatinine, urea, total protein, aspartate aminotransferase (ASAT), and alanine aminotransferase (ALAT) levels with the Konelab 30i analyser (Thermo Scientific, Cergy‐Pontoise, France) following the manufacturer's protocol. The plasma samples were kept at 4°C until analyzed with the Konelab 30i measurements and then stored at −80°C.

### 2.10 Urine sample and analysis

Urine was collected directly from the ureters for each kidney independently. The following results presented urinary parameters for each kidney separately, which were then combined for total analysis. The number of urines samples varied, as there were instances when no urine output was observed, and therefore no samples could be collected. The volume of urine output was measured, and the samples were stored at 4°C until urinary creatinine and protein levels were analyzed using the Konelab 30i. After these dosages, the urine samples were stored at −80°C for subsequent measurement of Neutrophil Gelatinase Associated Lipocalin (NGAL). NGAL was measured using the enzyme‐linked immunosorbent assay (ELISA) method. Fractional excretion of sodium (FENa) was also considered and calculated using the following formula:

$$\text{FENa}=\frac{\text{plasma creatinine}\times \text{urinary sodium}}{\text{urinary creatinine}\times \text{plasma sodium}}$$

The glomerular filtration rate (GFR; mL/min/kg) was calculated using the following formula:

$$\mathrm{GFR}=\frac{\text{urinary creatinine}\times \text{urine output}}{\text{plasma creatinine}\times \text{body weight}}$$

Enzyme‐linked immunosorbent assay (ELISA).

NGAL was quantified in urine using a commercially available porcine‐specific ELISA kit (Kit‐044; Bioporto diagnostics A/S, Copenhagen, Denmark) according to the manufacturer's instructions.

### 2.11 Acute kidney injury definition

KDIGO guidelines classify the severity of acute kidney injury (AKI) based on two criteria: plasma creatinine concentration and diuresis. However, due to time limitations in our experiment, we were unable to include the diuresis parameter as outlined in the guidelines. Instead, we applied a modified version of the score, referred to as ‘modified KDIGO’ or “mKDIGO”, which only considers plasma creatinine levels to assess the severity of AKI. This modified score was described by Gardner et al. in 2022.

The mKDIGO stages were defined as follows:

- Stage 1: the creatinine value at the end of the experiment was between 1.5 and 1.9 times the basal value.
- Stage 2: the creatinine value at the end of the experiment was between 2.0 and 2.9 times the basal value.
- Stage 3: Creatinine value at end of experiment greater than three times basal value

If the pigs did not exhibit an increase in creatinine greater than 1.5 times the baseline value, we concluded that they were not experiencing AKI.

### 2.12 Euthanasia and autopsy

At the end of the study, the pigs were euthanized intravenously using T61 (200 mg of embutramide, 26 mg of mebezonium, and 4.39 g of tetracaine; 0.1 mL/kg, Intervet 49,071 Beaucouze, France). Euthanasia was confirmed by monitoring the absence of pulse waves, a decrease in expired CO_2_, and the onset of apnea. After confirmation of euthanasia, the retroperitoneal spaces were accessed to collect kidney tissue samples. Transverse sections measuring 5 mm after trimming by pathologist were taken from the cortical, medullary, and corticomedullary junction areas. The sections were then fixed in 4% paraformaldehyde, paraffin‐embedded and preserved for histological analysis.

### 2.13 Histological analysis

After fixation and paraffin embedding, the tissues were stained with hematoxylin–eosin following standard procedures. Histological analysis was conducted by an experienced pathologist who was external to the team and blinded to the study protocol. Renal lesions were evaluated and scored based on the loss of proximal tubule brush border, microvacuolisation, presence of hyaline droplets, and presence of blebs. The scoring system used was as follows: 0 = absent, 1 = mild, 2 = moderate, 3 = marked, and 4 = severe (except for the loss of the brush border, which was scored as No = absent and Yes = present).

### 2.14 Statistical analysis

The distribution of quantitative variables was assessed using graphical representations. Since these variables did not follow a normal distribution, they are presented as medians and interquartile ranges (1st to 3rd quartile).

Linear mixed models were employed to examine the associations between parameters related to AKI (plasma creatinine, urea, and potassium concentrations) and both time and group (“IR” or “Sham”). In these models, plasma creatinine, urea, and potassium concentrations were treated as response variables, and Time was included as an explanatory variable (fixed effect), and the interaction between group and time was considered as a fixed effect. “Pig” was considered as a random effect. Residual's random distribution and homoscedasticity were checked by plotting residuals against fitted values as well as by creating histograms and QQ plots of the residuals. Results were exposed as estimates (regression coefficients) and their 95% confidence intervals (95% CI). To estimate the components of variance and covariance, REML (restricted maximum likelihood) was considered. Detailed models can be accessible in Figures S1–S11. Given that the variables did not follow a normal distribution, others quantitative parameters (ASAT to ALAT ratio, protein to creatinine ratio in urine (=uPCR), urinary NGAL, GFR, quantity of noradrenaline received) were compared using nonparametric tests. Comparisons between “Sham” and “IR” groups across time were conducted using the Kruskal–Wallis test. For paired data (GFR, uPCR, and diuresis) between the right and the left kidneys the Wilcoxon test was used. Fisher's exact test was applied to compare the proportion of pigs receiving noradrenaline, mKDIGO classification, and histologic scoring. The total amount of noradrenaline received was compared using the Mann–Whitney test.

Results were considered statistically significant when the *p*‐value was less than 0.05. Statistical analyses were performed using R version R 4.1.2 software (R Foundation for Statistical Computing, Vienna, Austria). The following R packages were utilized: “ggplot2” (Wickham, 2009), “ggpubr,” “ggstatsplot,” “Lme4” (Bates et al., 2015), “Lmertest” (Kuznetsova et al., 2017), and “cowplot”.

## 3 RESULTS

### 3.1 Missing data

Missing data for urinary analysis was due to anuria. No urinary data were available for GFR, uPCR, and NGAL at T0 (for IR2, IR4, and Sham1) and at T2h30 (for IR2 and IR4). Some data was missing from the analysis of diuresis, GFR, and uPCR from each kidney. Specifically, no urinary data was available for the right kidney in IR2 and IR4 because of anuria. Plasma data and MAP were also missing at T6h30, T7h, and T7h30 for Sham1 (a new catheter was inserted due to clotting of the femoral catheter). Missing data were excluded from the statistical analyses.

### 3.2 Glomerular filtration rate and diuresis

**FIGURE 2:**
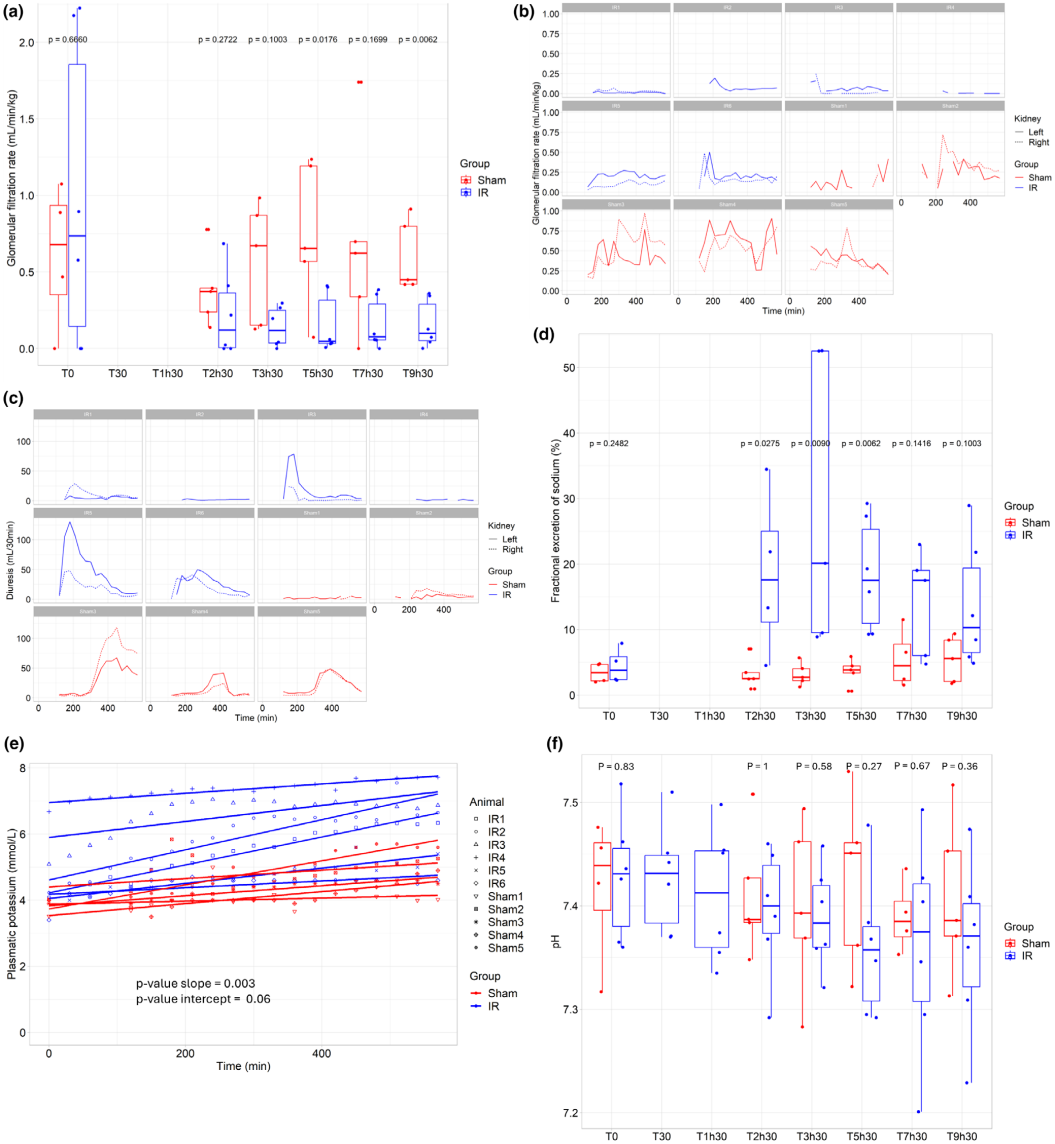
Evolution of renal function. (a) Glomerular filtration rate for both group (“IR” group (*N* = 6) and “Sham” group (*N* = 5)). At each timepoint, an analysis using Kruskall–Wallis test was performed. (b) Comparison of glomerular filtration rate for right and left kidney from each pig. (c) Comparison of diuresis for right and left kidney from each pig. (d) Fractional excretion of sodium for both group (“IR” group (*N* = 6) and “Sham” group (*N* = 5)). At each timepoint, an analysis using Kruskall–Wallis test was performed. (e) Plasma potassium in “IR” pigs and “Sham” pigs. Significancy was appreciated by linear mixed model (“IR” group (*N* = 6) and “Sham” group (*N* = 5)). (f) Potential hydrogen for “IR” pigs and “Sham” pigs (“IR” group (*N* = 6) and “Sham” group (*N* = 5)). At each timepoint, an analysis using Kruskall–Wallis test was performed.

The GFR was compared between groups at T0, T2h30, T3h30, T5h30, T7h30, and T9h30 in order to obtain a representative kinetic. At T0 (*n* = 4 for both group), there was no statistically significant difference between groups (*p* = 0.67): “Sham” = 0.66 mL/kg/min (0.47–0.89) and “IR” = 0.74 mL/kg/min (0.14–1.85) (Figure 2a). Glomerular filtration rate was significantly higher in the “Sham group” as compared to the “IR group” at T5h30 and T9h30 (*p* = 0.02 and *p* = 0.01, respectively).

Details on individual GFR and diuresis values are provided in Figure S2a,b. Concerning the comparison between kidneys per pig, the mean of GFR values across the experiment was significantly higher in the right kidney as compared to the left kidney for IR1 (*p* = 0.001). Conversely, GFR the left kidney as compared to the right kidney for IR3, IR5, and IR6 (*p* = 0.0002, *p* < 0.0001, and *p* = 0.001, respectively). Comparisons of GFR and diuresis between both kidneys are illustrated in Figure 2b.

Details on individual diuresis values are available in Figure S2b. Statistical analysis reveals no significant difference between group (Figure S2c). Diuresis mean values were significantly higher for the right kidney as compared to the left kidney for IR1 and Sham1 (respectively, *p* = 0.001 and *p* = 0.0086) and significantly higher for the left kidney as compared to the right kidney for IR3 and IR5 (respectively, *p* = 0.0001 and *p* = 0.02). Comparisons between kidneys for diuresis is illustrated in Figure 2c.

### 3.3 Fractional excretion of sodium

Fractional excretion of sodium (FENa) was not significantly different between the groups at T0 (*p* = 0.25) (Figure 2d). From T2h30 to T5h30, FENa was significantly higher in “IR” group as compared to “Sham” group (*p* = 0.03 at T2h30, *p* = 0.01 at T3h30, and *p* = 0.01 at T5h30).

### 3.4 Plasma potassium concentration

Plasma potassium increased significantly faster in “IR” group compared to “Sham” group (*p* = 0.003) (Figure 2e): the mean increase was 0.002 mmol/L/min (95% CI = [0.001; 0.002]) in “Sham group” and 0.003 mmol/L/min (95% CI = [0.002; 0.004] in “IR” group). Evolution of plasma potassium concentration over time and details of linear mixed model are available in Figure S2d,e. No significant difference was observed between the groups at T0 (*p* = 0.06).

### 3.5 Potential hydrogen and blood acidification

Potential hydrogen (pH) was not significantly different between the groups at T0 (*p* = 0.83) (Figure 2f). No significant difference was observed across time between “IR” group and “Sham” group (*p* = 0.27 at T5h30).

### 3.6 Plasma creatinine concentration and mKDIGO

**FIGURE 3:**
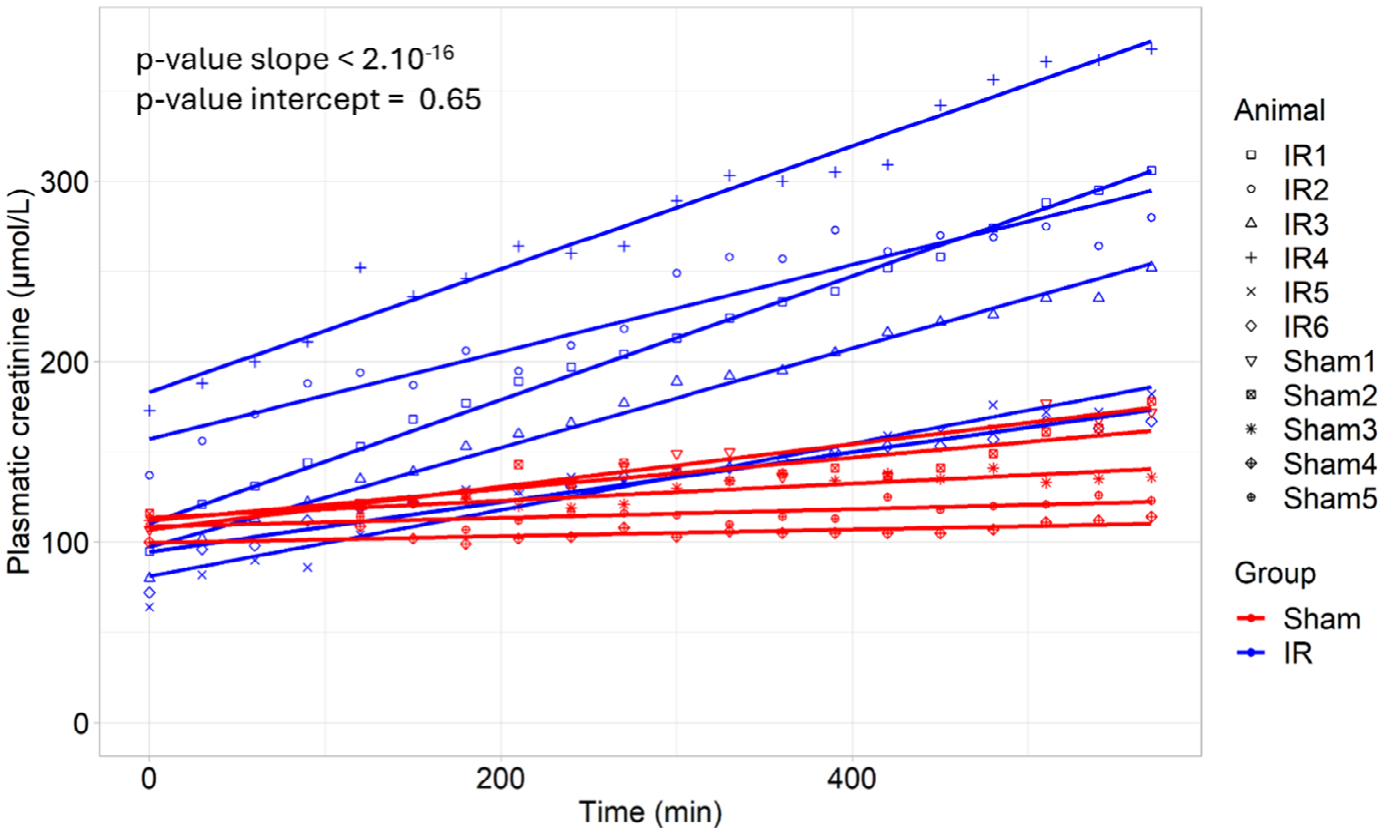
AKI severity determined with plasma creatinine. Plasma creatinine in “IR” group (*N* = 6) and “Sham” group (*N* = 5). Significancy was appreciated by linear mixed model.

Plasma creatinine concentration increased significantly faster in the “IR” group as compared to the “Sham” group (*p* < 0.0001): with a mean rate of 0.253 μmol/L/min (95% CI = [0.211; 0.294]) and 0.059 μmol/L/min (95% CI = [0.039; 0.076]), respectively (Figure 3). The evolution of the plasma creatinine concentration over time and details of the linear mixed model can be found in Figure S3a,b. No significant difference was observed between both group at T0 (*p* = 0.65).

Regarding the mKDIGO scores, in the “Sham group”: three pigs (3/5, 60%) did not present AKI, two pigs (2/5, 40%) had stage 1 AKI. In contrast, all pigs in the “IR group” developed AKI: four pigs (4/6, 67%) had a stage 2 AKI, and two pigs (2/6, 33%) had a stage 3 AKI (Table 1). The presence of AKI was higher in the “IR group” (100%) compared to the “Sham group” (40%). The difference was borderline significant (*p* = 0.06). Significantly more pigs experienced stage 2‐AKI or stage 3—AKI in the “IR group” (*p* = 0.002).

**TABLE 1:** AKI severity determined with plasma creatinine.

Animal	Group	Increase of plasma creatinine	mKDIGO classification
Sham1	Sham	1.61	Stage 1
Sham2	Sham	1.53	Stage 1
Sham3	Sham	1.21	Stage 0
Sham4	Sham	1.14	Stage 0
Sham5	Sham	1.14	Stage 0
IR1	IR	3.22	Stage 3
IR2	IR	2.31	Stage 2
IR3	IR	3.15	Stage 3
IR4	IR	2.16	Stage 2
IR5	IR	2.84	Stage 2
IR6	IR	2.32	Stage 2

*Note*: AKI severity for each pig according to mKDIGO. Increase of plasma creatinine was estimated by dividing value at T9h30 by T0.

### 3.7 Plasma urea concentration

**FIGURE 4:**
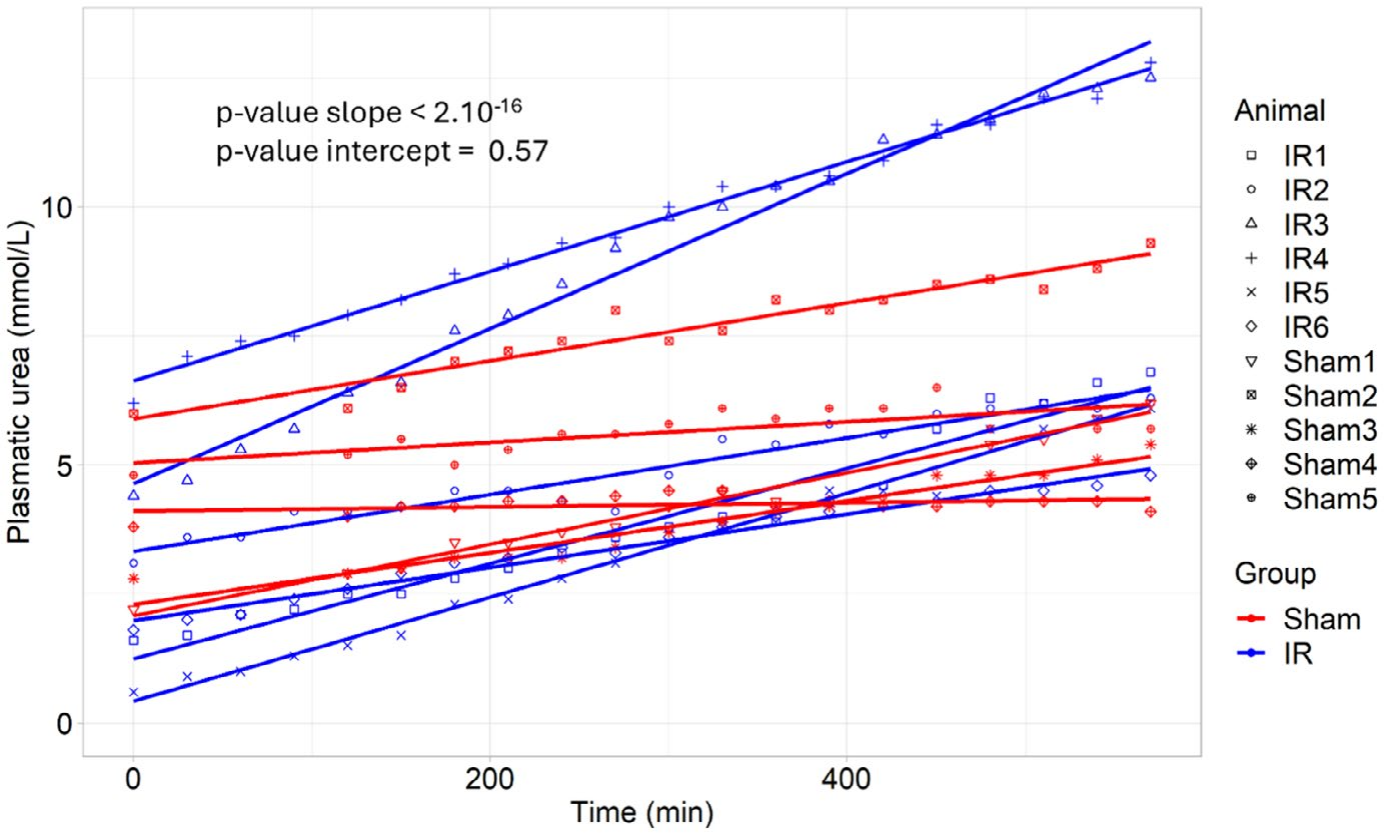
Plasma urea. Plasma urea in “IR” group and “Sham” group. Significancy was appreciated by linear mixed model (“IR” group (*N* = 6) and “Sham” group (*N* = 5)).

Plasma urea concentration increased significantly faster in “IR” group compared to “Sham” group (*p* < 0.0001) (Figure 4). The mean increase was 0.004 mmol/L/min (95% CI = [0.003; 0.005]) in the “Sham group” and 0.009 mmol/L/min (95% CI = [0.008; 0.011] in “IR” group). The evolution of plasma urea concentration over time and details of linear mixed model are available in Figure S4a,b. No significant difference was observed between both groups at T0 (*p* = 0.57).

### 3.8 ASAT/ALAT ratio

**FIGURE 5:**
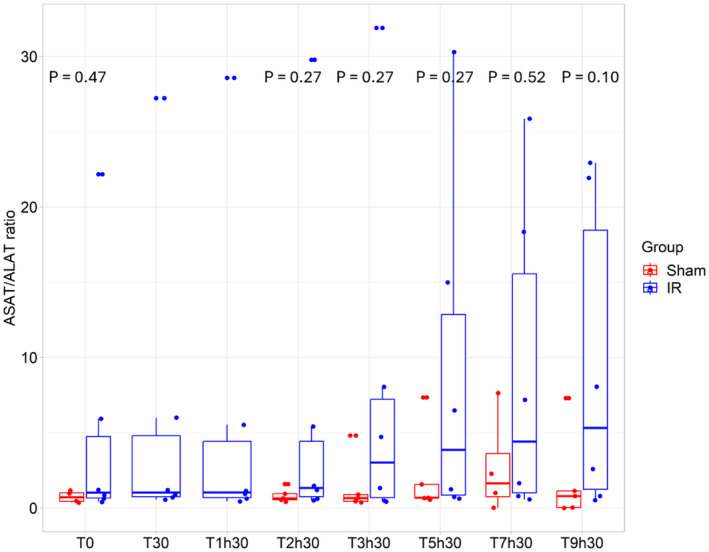
Evolution of ASAT/ALAT ratio. ASAT/ALAT ratio for both group (“IR” group (*N* = 6) and “Sham” group (*N* = 5)). At each timepoint, an analysis using Kruskall–Wallis test was performed.

No significant difference was observed between the groups at T0 (*p* = 0.47) (“Sham” = 0.72 (0.44–1.01) and “IR” = 0.83 (0.67–1.11)) (Figure 5). Although the ASAT/ALAT ratio increased over time in the “IR group”, it did not reach statistical significance (*p* = 0.1 at the end of the procedure, T9h30) (Figure 5).

### 3.9 Blood protein and hemoglobin

Plasma hemoglobin and plasma proteins remained within physiological range for both groups (Figure S6a,b).

### 3.10 Urine protein to creatinine ratio (uPCR)

**FIGURE 6:**
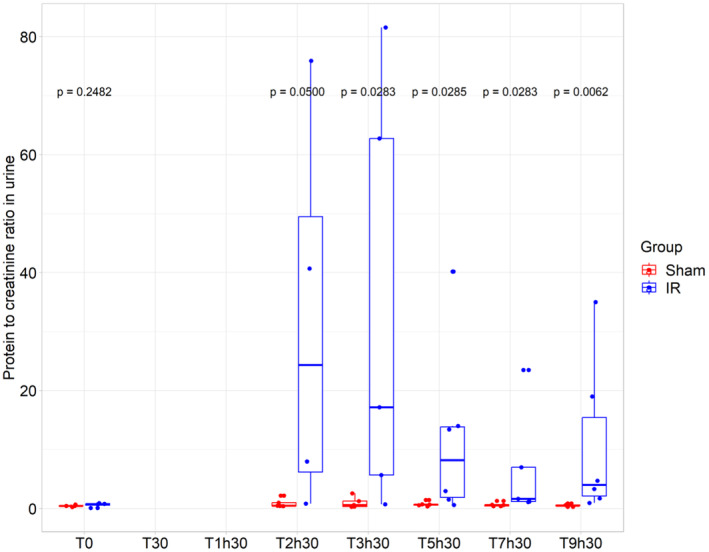
Evolution of protein to creatinine ratio in urine. Protein to creatinine ratio in urine for both group (“IR” group (*N* = 6) and “Sham” group (*N* = 5)). At each timepoint, an analysis using Kruskall–Wallis test was performed.

At T0, no statistically significant difference was observed between the groups (*p* = 0.25): “Sham” = 0.45 mL/kg/min (0.39–0.51) and “IR” = 0.87 mL/kg/min (0.78–0.94) (Figure 6). However, the uPCR was significantly higher in “IR group” as compared to “Sham group” from T2h30 to T9h30 (*p* = 0.05 at T2h30, *p* = 0.03 at T3h30, *p* = 0.03 at T5h30, *p* = 0.03 at T7h30, and *p* = 0.01 at T9h30) (Figure 6). Detailed uPCR data for each pig is available in Figure S7.

### 3.11 NGAL

**FIGURE 7:**
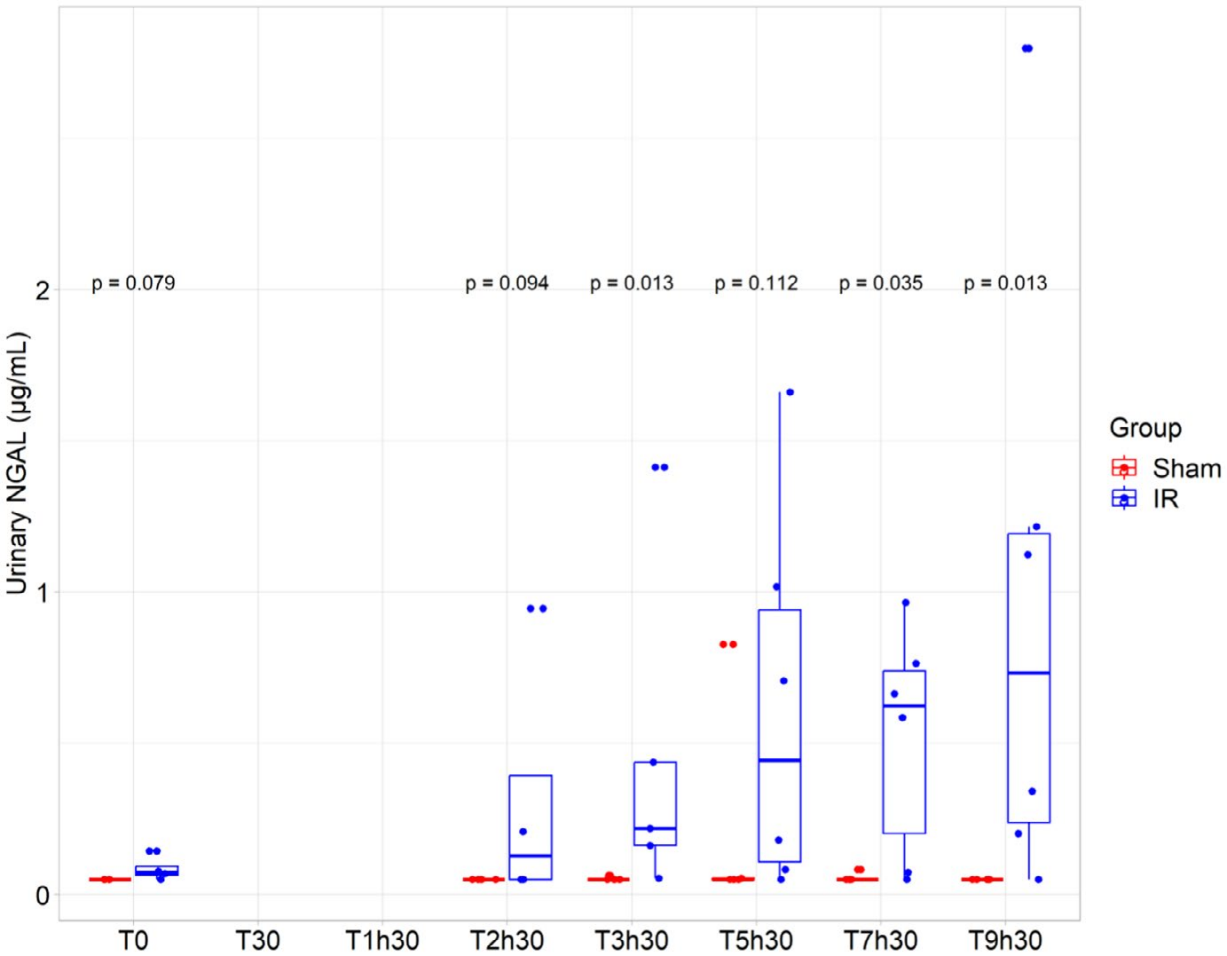
Evolution of urinary NGAL concentration. Urinary NGAL for both group (“IR” group (*N* = 6) and “Sham” group (*N* = 5)). At each timepoint, an analysis using Kruskall–Wallis test was performed.

At T0, no significant difference was found between the groups (*p* = 0.08): “Sham” = 0.05 nmol/L (0.05–0.05) and “IR” = 0.14 nmol/L (0.08–0.17) (Figure 7). Urinary NGAL levels were significantly higher in the “IR group” compared to the “Sham group” at T3h30, T7h30, and T9h30 (*p* = 0.01 at T3h30, *p* = 0.04 at T7h30, and *p* = 0.01 at T9h30) (Figure 7). The evolution of urinary NGAL concentration over time is provided in Figure S8.

### 3.12 Histological analysis

**FIGURE 8:**
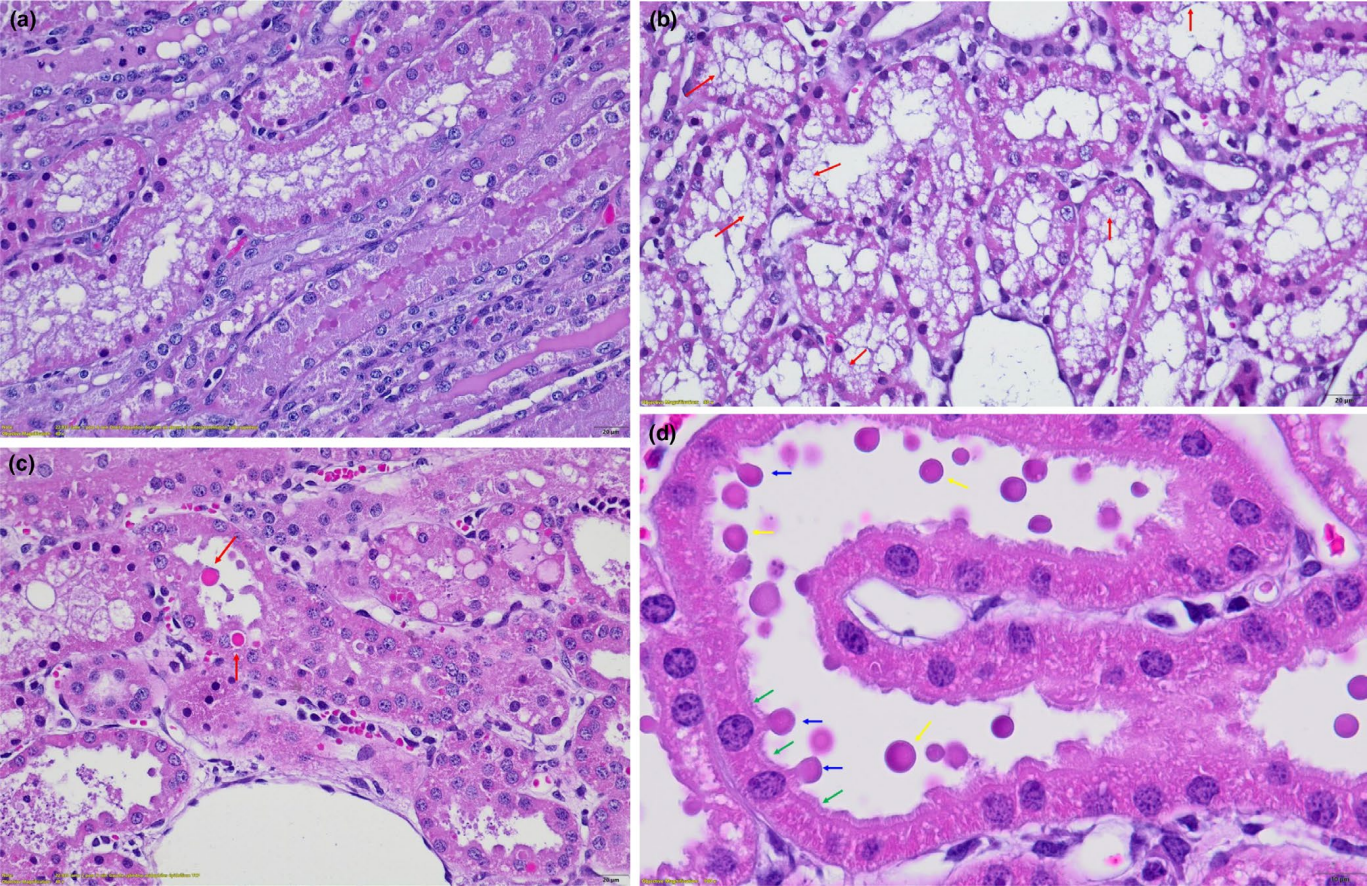
Histological description of each parameter. (a) Absence of tubular brush border. (b) Presence of microvacuolisation (revealed by red arrow). (c) Presence of hyaline droplets (revealed by red arrow). (d) Integrity of tubular brush border and tubular injury. Blue arrow designed blebs, yellow arrow designed amphophile vesicles and green arrow designed integer brush border.

There was no significant difference between the two groups in terms of brush border loss (*p* = 0.330) with 90% brush border loss in the “Sham” group and 69% in the “IR” group. Microvacuolisation was significantly higher in the “IR group” as compared to the “Sham group” (*p* = 0.004). The presence of hyalin droplets was also significantly higher in the “IR group” than in the “Sham group” (*p* = 0.004). Additionally, the occurrence of blebs was significantly greater in the “IR group” compared to the “Sham group” (*p* = 0.008). Detailed scoring and frequencies are provided in Table 2. Examples of brush border loss, microvacuolisation, and the presence of hyaline droplets and blebs are shown in Figure 8a–d.

**TABLE 2:** Histological scoring.

	Loss of brush border		
	Yes	No	
IR group	9 (69%)	4 (31%)	Fisher test: *p* = 0.33
Sham group	9 (90%)	1 (10%)	

	Tubular microvacuolisation					
	0	1	2	3	4	
IR group	0 (0%)	2 (15.3%)	2 (15.3%)	2 (15.3%)	7 (55%)	Fisher test: *p*‐value = 0.004
Sham group	5 (50%)	1 (10%)	2 (20%)	2 (20%)	0 (0%)	

	Presence of hyalin droplets					
	0	1	2	3	4	
IR group	3 (23%)	5 (38.5%)	5 (38.5%)	0 (0%)	0 (0%)	Fisher test: *p*‐value = 0.0004
Sham group	10 (100%)	0 (0%)	0 (0%)	0 (0%)	0 (0%)	

	Presence of blebs					
	0	1	2	3	4	
IR group	5 (38.5%)	2 (15.3%)	4 (31%)	2 (15.3%)	0 (0%)	Fisher test: *p*‐value = 0.008
Sham group	10 (100%)	0 (0%)	0 (0%)	0 (0%)	0 (0%)	

*Note*: Loss of brush border was qualified by “yes” or “no”. Other markers were qualified by a scoring (0, 1, 2, 3, and 4) which respectively involved “absence – mild – moderate – marked – severe”. An analysis of both right and left kidneys was done. Data were expressed as frequency of apparition (“IR” group (*N* = 6) and “Sham” group (*N* = 5)) and compared using Fisher exact test.

### 3.13 Arterial blood pressure

Mean arterial pressure of each pig was recorded and is available in Figure S9a. Comparison between groups across time is available in Figure S9b. At T0, no significant difference was observed (*p* = 0.1). No significant difference was observed across time between both groups (Figure S9b).

No significant difference was found between the “IR group” and the “Sham group” regarding the number of pigs that received noradrenaline (*p* = 0.55; Figure S9c). The total amount of noradrenaline administered was also not significantly different between the group (*p* = 0.93). Detailed data on noradrenaline usage is available in Figure S9d.

There was no association between noradrenaline infusion and plasma creatinine or urinary NGAL (details are presented in Figure S9e–h).

### 3.14 Glucose infusion

There was no association between glucose infusion and urinary NGAL (Figure S10a,b).

In the Sham group, plasma creatinine concentration increased significantly faster in pigs receiving glucose (*p* = 0.0006). The mean rate of increase was 0.036 μmol/L/min (95% CI = [0.019; 0.058]) for pigs not receiving glucose and 0.072 μmol/L/min (95% CI = [0.036; 0.110]) for pigs receiving glucose (Figure S10c).

In the IR group, plasma creatinine concentration increased significantly faster in pigs receiving glucose (*p* < 0.0001). The mean rate of increase was 0.227 μmol/L/min (95% CI = [0.208; 0.245]) for pigs not receiving glucose and 0.307 μmol/L/min (95% CI = [0.258; 0.359]) for pigs receiving glucose (Figure S10d).

### 3.15 Rectal temperature

Rectal temperature was compared between groups over time (Figure S11). No significant difference was observed at T0 (*p* = 0.27). No significant difference was observed between the two groups over time (Figure S11). None of the rectal temperatures recorded exceeded 39°C.

## 4 DISCUSSION

This study aimed to develop a porcine acute kidney injury (AKI) model using total intravenous anesthesia (TIVA). AKI was induced via ischemia–reperfusion through bilateral renal artery clamping. The protocol successfully generated moderate to severe AKI (stage 2–3), evidenced by elevated plasma creatinine and urea, reduced diuresis and glomerular filtration rate (GFR), and histological lesions consistent with IR injury. Stable plasma protein, hemoglobin levels, and increased FENa ruled out significant pre‐renal involvement. Increased uPCR and NGAL confirmed glomerular and tubular injury.

To prevent malignant hyperthermia (MH) associated with halogenated gases like sevoflurane, a TIVA protocol combining ketamine, medetomidine, and diazepam was employed. This approach avoided MH, improved hemodynamic stability crucial for AKI models, and reduced environmental impact by eliminating greenhouse gas use (Varughese & Ahmed, 2021). To the authors' knowledge, this is the first porcine AKI model utilizing TIVA. The surgical protocol requires technical expertise but avoids specialized equipment like balloons or contrast agents.

A 90‐min ischemia period was chosen based on literature data. Indeed, the aim of the study was to induce moderate‐to‐severe AKI. A period of ischemia that is too short does not induce sufficient lesions, whereas a period that is too long induces irreversible lesions. Gardner et al. proposed a mild‐to‐moderate model using 40 min of ischemia (Gardner et al., 2014). In the study of Laven et al., after contralateral nephrectomy, an ischemia period of 90 min was associated with moderate‐to‐severe AKI (Laven et al., 2004). Horikawa et al. showed that an ischemia period greater than 90 min (120 or 180 min) was associated with severe AKI (Horikawa et al., 2022).

GFR estimated by creatinine clearance is a key indicator of renal function and is commonly used to diagnose AKI. During renal ischemia–reperfusion (IR), cells experience hypoxia. Tubular cells, known for their sensitivity to hypoxia, exhibit decrease sodium reabsorption (Bonventre & Yang, 2011; Scholz et al., 2021). Following the activation of mediators such as adenosine, vasoconstriction occurs, leading to GFR reduction. Additionally, endothelial dysfunction during ischemia can cause excessive vasoconstriction, further reducing renal blood flow and GFR. In our model, the GFR was globally higher for “Sham group” as compared to “IR group”.

This finding aligns with other studies observing similar trends (Sølling et al., 2011). Humphreys et al. reported a reduction in renal function after 75 min of ischemia, which is consistent with our results (Humphreys et al., 2009). Haase et al. measured a GFR of around 0.25 mL/min/kg after 45 min of ischemia (Haase et al., 2009). The short ischemia may explain the higher GFR compared to our study.

Oliguria is a common feature of AKI induced by IR. Hypoxia‐induced endothelial damage leads to vasoconstriction, reducing GFR and eventually decreasing urine output. In this study, diuresis did not differ between the “Sham group” and “IR group”. However, with the exception of pig “IR2” and “IR4”, the other “IR” pigs exhibited a similar diuresis profile: an initial increase at the onset of reperfusion followed by a rapid decline. Interestingly, “IR2” and “IR4” developed continuous oliguria without any transient increase in diuresis at the beginning of reperfusion. A possible explanation for these two low diuresis levels could be related to the urine collection method. In this model, we catheterized the ureters, but it cannot be excluded that some of the urine produced leaked into the abdomen. Some studies have pointed out that measuring urine output can be challenging and may result in inaccurate assessments of diuresis (Allen et al., 2020; Gardner et al., 2022). In the Sham group, two pigs (“Sham 1 and 2”) exhibited poor diuresis, which appeared to be linked to low mean arterial pressure (MAP), around 50 mmHg. Many studies have shown that low MAP (below 55 mmHg) may be associated with the development of AKI (Lehman et al., 2010; Salmasi et al., 2017; Walsh et al., 2013). These two pigs spent a significant amount of time with MAP below 55 mmHg, explaining their poor diuresis.

Acute kidney injury (AKI) is commonly linked to acute tubular necrosis (ATN), particularly following ischemia–reperfusion (IR) injury. Normally, sodium is reabsorbed along the renal tubules, but during intrinsic AKI, such as ATN, this reabsorption is impaired, leading to increased fractional excretion of sodium (FENa). In this study, FENa increased significantly in the “IR” group, along with plasma creatinine and urinary NGAL, indicating that the AKI was intrinsic and likely associated with ATN.

In hypoxic conditions, kidney cells switch from aerobic metabolism to anaerobic metabolism (Blantz et al., 2007; Pefanis et al., 2023) leading to acidification of the blood pH (Alsaaty & Janabi, 2024). There was no significant difference in blood pH between the two groups, potentially due to adjustments in mechanical ventilation. The lack of significance could also stem from the small sample size and low statistical power, as some pigs in the IR group had lower pH values than those in the Sham group.

To compensate for this acidosis, kidney cells exchange intracellular potassium for excess blood protons. Eventually, due to glomerular and tubular damage, this excess of potassium accumulates in the blood. Like other publications, we observed an increase of plasma potassium in “IR group” as compared to “Sham group” (Bagshaw et al., 2008; Owji et al., 2018; Shum et al., 2016).

The severity of AKI is typically defined by urine output and plasma creatinine levels as per KDIGO guidelines. According to Gardner et al., only plasma creatinine levels were used to classify AKI severity (mKDIGO) as the classic KDIGO score requires extended monitoring of diuresis, which was not feasible here (Gardner et al., 2022). While creatinine is sometimes considered a late biomarker of AKI (Mishra et al., 2003; Waikar et al., 2009), our results showed an early increase in this parameter, starting at the onset of ischemia. Malagrino et al. observed a similar early rise in plasma creatinine following unilateral ischemia for 2 h using a balloon, and Gardner et al. reported an early increase following 40 min of bilateral arterial occlusion. They also showed an early increase in plasma creatinine from induction of ischemia to 8 h of reperfusion. After this time, the increase slowed down (Gardner et al., 2014; Malagrino et al., 2014).

Some studies have also acknowledged that AKI may be accompanied by other dysfunction, such as liver damage (Gardner et al., 2016; Lane et al., 2013). Our model showed an increase in plasma ASAT, a marker of liver damage. The ASAT‐to‐ALAT ratio, (De Ritis ratio), has previously been shown to increase following AKI, as observed in our study (Pilarczyk et al., 2020). Interestingly, Gultekin et al. suggested that this ratio correlates with AKI severity, especially after cardiopulmonary bypass or cardiac surgery (Gultekin et al., 2021).

Under normal conditions, there is no protein in the urine. However, in AKI, glomerular filtration is impaired, and tubular cell damage prevents proper reabsorption, resulting in protein leakage into the urine. Compared to Malagrino's study, the uPCR peak in our model was more pronounced, consistent with a more severe injury model (Malagrino et al., 2014). Although glomerular injury can be observed in AKI, it is less relevant than tubular injury. Most AKI could be attributed to acute tubular necrosis.

Neutrophil Gelatinase Associated Lipocalin (NGAL) is a reliable marker of tubular injury, fastly detectable and specific (Schrezenmeier et al., 2017; Silberstein et al., 2013). Numerous studies in human and preclinical models have confirmed the effectiveness of NGAL as a biomarker of AKI, particularly following ischemia–reperfusion (IR) injury which is commonly observed after cardiac surgery (Bennett et al., 2008; Elitok et al., 2022; Gagneux‐Brunon et al., 2012; Gardner et al., 2014; Haase et al., 2009; Malagrino et al., 2014; Shang & Wang, 2017; Silberstein et al., 2013). NGAL has been shown to increase early after IR injury (Mishra et al., 2003; Supavekin et al., 2003). In experimental research, urinary NGAL is typically measured whereas plasma NGAL is more used in clinical studies (Gagneux‐Brunon et al., 2012). However, both are now often used in experimental settings due to their comparable concentrations in urine and blood under physiological conditions (Virzì et al., 2013). As Silberstein's study suggested, urinary NGAL might be more sensitive than plasma NGAL, as it is delivered directly from injured tubules (Silberstein et al., 2013). For this reason, we only measured urinary NGAL in our study. Our model showed an increase in urinary NGAL in the “IR” group compared to the “Sham” group. This rise in NGAL likely began during ischemia (though we could not observe it due to the lack of diuresis) and continued during the reperfusion as demonstrated in other studies where NGAL was measured in blood (Gardner et al., 2014; Silberstein et al., 2013). Interestingly, Malagrino et al. reported variations in serum NGAL during ischemia and reperfusion; although, unlike other studies, these changes did not provide more valuable information than traditional creatinine measurements (Malagrino et al., 2014).

As previously reported, IR leading to AKI is characterized by a hypoxic period leading to various metabolic changes (Pefanis et al., 2023). Hypoxia reduces ATP levels by shifting aerobic metabolism to anaerobic metabolism, resulting in ATPase inhibition (Blantz et al., 2007). Tubular reabsorption is dependent on the by the proper functioning of these pumps (Ostermann & Liu, 2017) and in hypoxic conditions, reabsorption is impaired. It is therefore well established that tubular cells are highly sensitive to hypoxia during IR (Bonventre & Yang, 2011; Makris & Spanou, 2016). A key marker of tubular injury in humans and animal models is brush border loss. In this study, no significant difference was observed between the Sham and IR groups. Moreover, the proportion was higher in the Sham group. This result is surprising. It is possible that parameters such as the delay between euthanasia and kidney sampling influenced the disappearance of the brush border. Although the time between euthanasia and tissue collection was minimized, it may have been sufficient to induce brush border loss. Euthanasia stops blood flow, and as brush border loss is an early indicator of tubular injury, this could explain its occurrence even in the Sham group (Basile et al., 2012; Molitoris, 1991). The loss of the brush border was observed in animals that developed hypoglycemia and could be linked to hypoglycemia, potentially explaining the concurrent rise in creatinine levels.

In line with other studies (Fogo et al., 2016; Gerosa, 2015; Owji et al., 2018), we also considered tubular microvacuolisation as another marker of tubular injury. Gerosa et al., observed these changes in pigs following hypoxia‐reperfusion episodes (Gerosa, 2015). Microvacuolisation was significantly increased in the “IR” group, confirming a tubular damage. Additionally, Barros et al., described plasma membrane blebs as another marker of tubular injury. These are bubble‐like protrusions that appear after hypoxia (Barros et al., 2003). ATP depletion leads to destabilization of actin filaments between the cytoskeleton and the plasma membrane. Our model showed a significant presence of these blebs in the “IR” group, suggesting severe injury induced by our protocol. As previously reported uPCR increases after AKI, and due to impaired protein reabsorption resulting from tubular damage, eosinophilic vesicles (hyaline droplets containing proteins) are observed in human biopsies (Sato et al., 2005). Even when tubular cells are injured, they can still partially perform endocytosis, leading to the internalization of proteins into intracellular vesicles. This finding was replicated in our study. Although we did not quantify tubular dilatation due to technical limitations, it is a useful marker of injury that should have been considered (Brunswig‐Spickenheier et al., 2010; Gardner et al., 2014; Horikawa et al., 2022; Malagrino et al., 2014). Finally, inflammatory cell infiltration of the interstitium is a common marker used to assess tubular injury in many studies and it remains an important marker to consider (Baldwin et al., 2004; Brunswig‐Spickenheier et al., 2010; Gardner et al., 2014; Horikawa et al., 2022).

The anesthetic protocol used in a preclinical model of AKI can impact the model's reliability by inducing cardiocirculatory dysfunction and other complications. Acute kidney injury can have a prerenal origin, as seen in case of dehydration or hemodynamic failure (Sun et al., 2015; Walsh et al., 2013) leading to poor kidney perfusion, and resulting in AKI. In our model, hemoglobin and plasma proteins remained within the physiological range in our model. The absence of hemoconcentration suggests that dehydration may not be the cause of this AKI. We also performed fluid infusion to prevent dehydration and managed MAP to reduce kidneys hypoperfusion. However, two “Sham” pigs that developed stage 1 AKI experienced hypotension periods likely leading to renal hypoperfusion though the lack of NGAL increase supports this. This hypoperfusion may have contributed to renal damage.

The anesthetic protocol included glucose and noradrenaline supplementation for hypoglycemia or hypotension. While noradrenaline use was not linked to AKI progression, glucose infusion was associated with more severe AKI, as evidenced by higher plasma creatinine levels. This suggests that hypoglycemia or glucose infusion may contribute to AKI progression. Hypoglycemia might also result from AKI, as brush border injury impairs glucose reabsorption by proximal tubular cells (Malagrino et al., 2014; Wen et al., 2021). Additionally, AKI disrupts renal cortex gluconeogenesis, which accounts for 30% of total gluconeogenesis (Legouis et al., 2020; Serraino et al., 2021). However, hypoglycemia also occurred in the Sham group, making AKI‐induced hypoglycemia unlikely in this study. Total intravenous anesthesia may also prevent the occurrence of MH, which can affect the stability of pigs and compromise the reliability of a model. In this study, no pigs exhibited clinical signs of MH, as indicated by the absence of a rectal temperature greater than 39°C.

This study demonstrated variability between pigs, particularly in their response to IR. Differences in GFR were observed between groups, but as the pigs were of similar age, this is unlikely to be age‐related. A sex effect could explain some of the variability as females are more resistant to ischemic injury due to sex hormones (Liang & Liu, 2023; Nemours et al., 2022). However, the pigs in this study were 3 months old and probably prepubertal (Dhondt et al., 2020; Giraud et al., 2011), making hormone‐related effects unlikely and we did not find any effect of sex on renal parameters in our study. Variability in anesthetic response was also observed. Cytochromes involved in ketamine metabolism, such as CYP2C, differ by sex, with males having higher activity (Helke et al., 2016; Skaanild & Friis, 2008). Females may experience ketamine accumulation, which can lead to hypoglycemia (Parker et al., 2023; Sharif & Abouazra, 2009). In this study, females might exhibit more severe hypoglycemia, associated with tubular injury as indicated by elevated plasma creatinine. Genetic variability cannot be excluded, but is unlikely as the pigs were from the same lineage.

Compared to AKI observed after cardiac surgery in humans, the progression of parameters measured in this study aligns closely with human data. For instance, the urinary NGAL levels recorded in this study are similar to those seen in humans post‐cardiac surgery (Moriyama et al., 2016). While baseline biochemical parameters such as plasma creatinine are comparable between humans and pigs (Giraud et al., 2011), their dynamics during AKI appear to differ (Dhondt et al., 2020). Plasma creatinine, a known late marker of AKI in humans (Abbas et al., 2023; Mishra et al., 2003; Slocum et al., 2012), showed an early increase during the ischemic phase in this study. This early elevation suggests that the AKI induced here is of moderate to severe intensity, whereas many cardiac surgery‐related AKIs in humans are less severe, as defined by plasma creatinine increases (Howitt et al., 2018; Machado et al., 2014; Wong et al., 2015).

Histological markers of AKI observed in this porcine model appear consistent with those reported in human studies (Kudose et al., 2018). However, further research is necessary to confirm these findings, as histological data in humans are challenging to obtain due to the invasive nature of biopsies (Thiele et al., 2015).

The TIVA protocol offers the advantage of hemodynamic stabilization in pigs while preventing malignant hyperthermia (Lehman et al., 2010; Sun et al., 2015; Walsh et al., 2013). However, the anesthetic protocol has its limitations. Indeed, this protocol is rarely used in humans, which reduces the possibility of comparing our results to those observed during cardiac surgery. Furthermore, since the protocol used is not well‐documented, it is associated with unexpected effects, such as hypoglycemia potentially linked to the administration of high doses of ketamine.

### 4.1 Our study had some limitations

The small number of animals included limits the statistical power of some analyses performed. This limited sample size is based on the power calculation presented earlier and is in line with the principle of reducing the number of animals used in biomedical research (3Rs rule). The small sample size may have limited the study, given the observed biological variability. Moreover, the study included both males and females, which increases genetic variability. We made this choice because AKI is not a complication specifically associated with either sex in humans. However, it is possible that a sex effect may have been present.

Anesthesia was shorter in the Sham group, which may have affected the comparisons through its effect on the development of AKI. Indeed, it is highly likely that biomarkers such as creatinine would have been higher if the anesthesia duration had exceeded 90 min in the Sham group. Based on the results of the linear model, creatinine levels in the Sham group at the end of the study would be expected to increase by an average of 5.31 μmol/L. Thus, the effect appears to be of minor significance. Furthermore, rather than comparing final values, we chose to use models and focus on the kinetics of increase (the slope of the lines). This parameter is not affected by the total duration of anesthesia, as the increase in creatinine levels over time is linear. Strict control of TIVA parameters is necessary to maintain MAP, glycemia and vital signs within physiological ranges.

Missing urine data from one Sham and two IR pigs may have affected the results and were excluded from statistical analysis. This missing data were related to anuria following renal dysfunction rather than technical problems. Finally, the study lacked long‐term follow‐up, which made it impossible to tell whether or not the AKI was reversible and in the event that it was not reversible, to assess the progression of renal injury or the transition from AKI to chronic kidney disease.

## 5 CONCLUSION

In summary, our study introduces a new preclinical porcine model of surgically induced AKI with IR using TIVA, eliminating the need for specialized equipment. According to the mKDIGO classification, this model induces moderate‐to‐severe AKI, closely mimicking AKI related to bypass surgery in humans. Given the severity of the model, it could meet the requirements for implementing and studying extracorporeal renal replacement therapy. It could also be used for extended follow‐up to investigate fibrosis development, typically observable after several days of reperfusion.

The advantage of setting up an AKI model is that it can be used to test strategies for preventing the onset of ischaemic AKI, as is the case, for example, when bypass surgery is performed in humans. Moreover, the severity of the AKI model enables the evaluation of interventions such as extracorporeal purification. Additionally, it is conceivable that the animals could be allowed to recover instead of being sacrificed at the end of the study, enabling the monitoring of experimental AKI progression over time.

## FUNDING INFORMATION

The authors have reported financial support for this study: BPI (public investment bank) and VetAgro Sup.

## CONFLICT OF INTEREST STATEMENT

The authors declare no conflict of interest.

## Supporting information


Figure S1.

## Data Availability

The data supporting the results of this study are available on request from the corresponding author.

## References

[phy270203-bib-0001] Abbas, Q. , Laghari, P. , Jurair, H. , Nafis, J. , Saeed, B. , Qazi, M. F. , Saleem, A. , Khan, A. H. H. , & Haque, A. (2023). Neutrophil gelatinase‐associated lipocalin as a predictor of acute kidney injury in children with shock: A prospective study. Cureus, 15, e34407. 10.7759/cureus.34407 36874735 PMC9977468

[phy270203-bib-0002] Allen, J. C. , Gardner, D. S. , Skinner, H. , Harvey, D. , Sharman, A. , & Devonald, M. A. J. (2020). Definition of hourly urine output influences reported incidence and staging of acute kidney injury. BMC Nephrology, 21, 19. 10.1186/s12882-019-1678-2 31941447 PMC6964092

[phy270203-bib-0003] Alsaaty, E. H. , & Janabi, A. M. (2024). Moexipril improves renal ischemia/reperfusion injury in adult male rats. Journal of Contemporary Medical Sciences, 10, 25–30. 10.22317/jcms.v10i1.1477

[phy270203-bib-0004] Bagshaw, S. M. , George, C. , Bellomo, R. , & the ANZICS Database Management Committee . (2008). Early acute kidney injury and sepsis: A multicentre evaluation. Critical Care, 12, R47. 10.1186/cc6863 18402655 PMC2447598

[phy270203-bib-0005] Baldwin, D. D. , Maynes, L. J. , Berger, K. A. , Desai, P. J. , Zuppan, C. W. , Zimmerman, G. J. , Winkielman, A. M. , Sterling, T. H. , Tsai, C. K. , & Ruckle, H. C. (2004). Laparoscopic warm renal ischemia in the solitary porcine kidney model. Urology, 64, 592–597. 10.1016/j.urology.2004.04.019 15351615

[phy270203-bib-0006] Barros, L. F. , Kanaseki, T. , Sabirov, R. , Morishima, S. , Castro, J. , Bittner, C. X. , Maeno, E. , Ando‐Akatsuka, Y. , & Okada, Y. (2003). Apoptotic and necrotic blebs in epithelial cells display similar neck diameters but different kinase dependency. Cell Death and Differentiation, 10, 687–697. 10.1038/sj.cdd.4401236 12761577

[phy270203-bib-0007] Basile, D. P. , Anderson, M. D. , & Sutton, T. A. (2012). Pathophysiology of acute kidney injury. Comprehensive Physiology, 2, 1303–1353. 10.1002/cphy.c110041 23798302 PMC3919808

[phy270203-bib-0008] Bates, D. , Mächler, M. , Bolker, B. , & Walker, S. (2015). Fitting linear mixed‐effects models using lme4. Journal of Statistical Software, 67, 1–48. 10.18637/jss.v067.i01

[phy270203-bib-0009] Bechara, G. R. , Damasceno‐Ferreira, J. A. , Abreu, L. A. D. S. , Costa, W. S. , Sampaio, F. J. B. , Pereira‐Sampaio, M. A. , & Souza, D. B. D. (2016). Glomerular loss after arteriovenous and arterial clamping for renal warm ischemia in a swine model. Acta Cirúrgica Brasileira, 31, 753–758. 10.1590/s0102-865020160110000008 27982263

[phy270203-bib-0010] Bennett, M. , Dent, C. L. , Ma, Q. , Dastrala, S. , Grenier, F. , Workman, R. , Syed, H. , Ali, S. , Barasch, J. , & Devarajan, P. (2008). Urine NGAL predicts severity of acute kidney injury after cardiac surgery: A prospective study. Clinical Journal of the American Society of Nephrology, 3, 665–673. 10.2215/CJN.04010907 18337554 PMC2386703

[phy270203-bib-0011] Blantz, R. C. , Deng, A. , Miracle, C. M. , & Thomson, S. C. (2007). Regulation of kidney function and metabolism: A question of supply and demand. Transactions of the American Clinical and Climatological Association, 118, 23–43. 18528487 PMC1863590

[phy270203-bib-0012] Bonventre, J. V. , & Yang, L. (2011). Cellular pathophysiology of ischemic acute kidney injury. The Journal of Clinical Investigation, 121, 4210–4221. 10.1172/JCI45161 22045571 PMC3204829

[phy270203-bib-0013] Brunswig‐Spickenheier, B. , Boche, J. , Westenfelder, C. , Peimann, F. , Gruber, A. D. , Jaquet, K. , Krause, K. , Zustin, J. , Zander, A. R. , & Lange, C. (2010). Limited immune‐modulating activity of porcine mesenchymal stromal cells abolishes their protective efficacy in acute kidney injury. Stem Cells and Development, 19, 719–729. 10.1089/scd.2009.0494 20143956

[phy270203-bib-0014] Dhondt, L. , Croubels, S. , De Paepe, P. , Wallis, S. C. , Pandey, S. , Roberts, J. A. , Lipman, J. , De Cock, P. , & Devreese, M. (2020). Conventional pig as animal model for human renal drug excretion processes: Unravelling the porcine renal function by use of a cocktail of exogenous markers. Frontiers in Pharmacology, 11, 883. 10.3389/fphar.2020.00883 32595506 PMC7303324

[phy270203-bib-0015] Ducheyron, D. , Terzi, N. , & Charbonneau, P. (2008). Les nouveaux marqueurs biologiques de l'insuffisance rénale aiguë. Réanimation, 17, 775–782. 10.1016/j.reaurg.2008.09.011

[phy270203-bib-0016] Ebert, T. J. , Harkin, C. P. , & Muzi, M. (1995). Cardiovascular responses to sevoflurane: A review. Anesthesia and Analgesia, 81, 11S–22S. 10.1097/00000539-199512001-00003 7486143

[phy270203-bib-0017] Elitok, S. , Devarajan, P. , Bellomo, R. , Isermann, B. , Haase, M. , & Haase‐Fielitz, A. (2022). NGAL/hepcidin‐25 ratio and AKI subtypes in patients following cardiac surgery: A prospective observational study. Journal of Nephrology, 35, 597–605. 10.1007/s40620-021-01063-5 34028701 PMC8926978

[phy270203-bib-0018] Fogo, A. B. , Lusco, M. A. , Najafian, B. , & Alpers, C. E. (2016). AJKD atlas of renal pathology: Ischemic acute tubular injury. American Journal of Kidney Diseases, 67, e25. 10.1053/j.ajkd.2016.03.003 27091020

[phy270203-bib-0019] Fu, Y. , Tang, C. , Cai, J. , Chen, G. , Zhang, D. , & Dong, Z. (2018). Rodent models of AKI‐CKD transition. American Journal of Physiology. Renal Physiology, 315, F1098–F1106. 10.1152/ajprenal.00199.2018 29949392 PMC6230729

[phy270203-bib-0020] Gagneux‐Brunon, A. , Delanaye, P. , Legrand, D. , Cavalier, E. , & Mariat, C. (2012). NGAL, biomarqueur de lésion rénale: point d’étape en 2012. Néphrologie Thérapeutique, 8, 508–515. 10.1016/j.nephro.2012.03.006 22541989

[phy270203-bib-0021] Gardner, D. S. , Allen, J. C. , Goodson, D. , Harvey, D. , Sharman, A. , Skinner, H. , Szafranek, A. , Young, J. S. , Bailey, E. H. , & Devonald, M. A. J. (2022). Urinary trace elements are biomarkers for early detection of acute kidney injury. Kidney International Reports, 7, 1524–1538. 10.1016/j.ekir.2022.04.085 35812272 PMC9263416

[phy270203-bib-0022] Gardner, D. S. , De Brot, S. , Dunford, L. J. , Grau‐Roma, L. , Welham, S. J. M. , Fallman, R. , O'Sullivan, S. E. , Oh, W. , & Devonald, M. A. J. (2016). Remote effects of acute kidney injury in a porcine model. American Journal of Physiology. Renal Physiology, 310, F259–F271. 10.1152/ajprenal.00389.2015 26608790

[phy270203-bib-0023] Gardner, D. S. , Welham, S. J. M. , Dunford, L. J. , McCulloch, T. A. , Hodi, Z. , Sleeman, P. , O'Sullivan, S. , & Devonald, M. A. J. (2014). Remote conditioning or erythropoietin before surgery primes kidneys to clear ischemia‐reperfusion‐damaged cells: A renoprotective mechanism? American Journal of Physiology. Renal Physiology, 306, F873–F884. 10.1152/ajprenal.00576.2013 24523383 PMC3989632

[phy270203-bib-0024] Gerosa, C. (2015). Histopathology of renal asphyxia in newborn piglets: Individual susceptibility to tubular changes. World Journal of Nephrology, 4, 313–318. 10.5527/wjn.v4.i2.313 25949946 PMC4419142

[phy270203-bib-0025] Giraud, S. , Favreau, F. , Chatauret, N. , Thuillier, R. , Maiga, S. , & Hauet, T. (2011). Contribution of large pig for renal ischemia‐reperfusion and transplantation studies: The preclinical model. Journal of Biomedicine & Biotechnology, 2011, 1–14. 10.1155/2011/532127 PMC3051176 21403881

[phy270203-bib-0026] Gultekin, Y. , Bolat, A. , Hatice, K. , & Tekeli Kunt, A. (2021). Does aspartate aminotransferase to alanine aminotransferase ratio predict acute kidney injury after cardiac surgery? Heart surg. Forum, 24, E506–E511. 10.1532/hsf.3849 34173741

[phy270203-bib-0027] Haase, M. , Bellomo, R. , Devarajan, P. , Schlattmann, P. , & Haase‐Fielitz, A. (2009). Accuracy of neutrophil gelatinase‐associated lipocalin (NGAL) in diagnosis and prognosis in acute kidney injury: A systematic review and meta‐analysis. American Journal of Kidney Diseases, 54, 1012–1024. 10.1053/j.ajkd.2009.07.020 19850388

[phy270203-bib-0028] Helke, K. L. , Nelson, K. N. , Sargeant, A. M. , Jacob, B. , McKeag, S. , Haruna, J. , Vemireddi, V. , Greeley, M. , Brocksmith, D. , Navratil, N. , Stricker‐Krongrad, A. , & Hollinger, C. (2016). Pigs in toxicology: Breed differences in metabolism and background findings. Toxicologic Pathology, 44, 575–590. 10.1177/0192623316639389 27044377

[phy270203-bib-0029] Hesketh, E. E. , Czopek, A. , Clay, M. , Borthwick, G. , Ferenbach, D. , Kluth, D. , & Hughes, J. (2014). Renal Ischaemia reperfusion injury: A mouse model of injury and regeneration. Journal of Visualized Experiments, 88, 51816. 10.3791/51816 PMC4188040 24961244

[phy270203-bib-0030] Hobson, C. E. , Yavas, S. , Segal, M. S. , Schold, J. D. , Tribble, C. G. , Layon, A. J. , & Bihorac, A. (2009). Acute kidney injury is associated with increased long‐term mortality after cardiothoracic surgery. Circulation, 119, 2444–2453. 10.1161/CIRCULATIONAHA.108.800011 19398670

[phy270203-bib-0031] Horikawa, T. , Maehata, J. , Hashimoto, F. , Ikuhara, T. , Araki, H. , Umeno, H. , Sano, Y. , Ishikawa, C. , Takagi, H. , Watanabe, A. , & Koizumi, T. (2022). Constructing a continuous hemodiafiltration‐type circulatory model of acute kidney injury in pigs. Therapeutic Apheresis and Dialysis, 26, 507–514. 10.1111/1744-9987.13826 35247221 PMC9310588

[phy270203-bib-0032] Howitt, S. H. , Grant, S. W. , Caiado, C. , Carlson, E. , Kwon, D. , Dimarakis, I. , Malagon, I. , & McCollum, C. (2018). The KDIGO acute kidney injury guidelines for cardiac surgery patients in critical care: A validation study. BMC Nephrology, 19, 149. 10.1186/s12882-018-0946-x 29940876 PMC6020229

[phy270203-bib-0033] Huang, J. , Bayliss, G. , & Zhuang, S. (2021). Porcine models of acute kidney injury. American Journal of Physiology. Renal Physiology, 320, F1030–F1044. 10.1152/ajprenal.00022.2021 33900853 PMC8285645

[phy270203-bib-0034] Huang, J. , Cao, H. , Cui, B. , Ma, X. , Gao, L. , Yu, C. , Shen, F. , Yang, X. , Liu, N. , Qiu, A. , Cai, G. , & Zhuang, S. (2022). Mesenchymal stem cells‐derived exosomes ameliorate ischemia/reperfusion induced acute kidney injury in a porcine model. Frontiers in Cell and Development Biology, 10, 899869. 10.3389/fcell.2022.899869 PMC9171021 35686052

[phy270203-bib-0035] Huang, Q. , Niu, Z. , Tan, J. , Yang, J. , Liu, Y. , Ma, H. , Lee, V. W. S. , Sun, S. , Song, X. , Guo, M. , Wang, Y. , & Cao, Q. (2015). IL‐25 elicits innate lymphoid cells and multipotent progenitor type 2 cells that reduce renal ischemic/reperfusion injury. Journal of the American Society of Nephrology, 26, 2199–2211. 10.1681/ASN.2014050479 25556172 PMC4552110

[phy270203-bib-0036] Humphreys, M. R. , Castle, E. P. , Lohse, C. M. , Sebo, T. J. , Leslie, K. O. , & Andrews, P. E. (2009). Renal ischemia time in laparoscopic surgery: An experimental study in a porcine model. International Journal of Urology, 16, 105–109. 10.1111/j.1442-2042.2008.02173.x 19120531

[phy270203-bib-0037] Khwaja, A. (2012). KDIGO clinical practice guidelines for acute kidney injury. Nephron. Clinical Practice, 120, c179–c184. 10.1159/000339789 22890468

[phy270203-bib-0038] Kudose, S. , Hoshi, M. , Jain, S. , & Gaut, J. P. (2018). Renal histopathologic findings associated with severity of clinical acute kidney injury. The American Journal of Surgical Pathology, 42, 625–635. 10.1097/PAS.0000000000001028 29537990

[phy270203-bib-0039] Kuznetsova, A. , Brockhoff, P. B. , & Christensen, R. H. B. (2017). lmerTest package: Tests in linear mixed effects models. Journal of Statistical Software, 82, 1–26. 10.18637/jss.v082.i13

[phy270203-bib-0040] Lane, K. , Dixon, J. J. , MacPhee, I. A. M. , & Philips, B. J. (2013). Renohepatic crosstalk: Does acute kidney injury cause liver dysfunction? Nephrology, Dialysis, Transplantation, 28, 1634–1647. 10.1093/ndt/gft091 23685679

[phy270203-bib-0041] Laven, B. A. , Orvieto, M. A. , Chuang, M. S. , Ritch, C. R. , Murray, P. , Harland, R. C. , Inman, S. R. , Brendler, C. B. , & Shalhav, A. L. (2004). Renal TOLERANCE to prolonged warm ischemia time in a laparoscopic VERSUS open surgery porcine model. The Journal of Urology, 172, 2471–2474. 10.1097/01.ju.0000138158.16968.8d 15538293

[phy270203-bib-0042] Legouis, D. , Ricksten, S.‐E. , Faivre, A. , Verissimo, T. , Gariani, K. , Verney, C. , Galichon, P. , Berchtold, L. , Feraille, E. , Fernandez, M. , Placier, S. , Koppitch, K. , Hertig, A. , Martin, P.‐Y. , Naesens, M. , Pugin, J. , McMahon, A. P. , Cippà, P. E. , & De Seigneux, S. (2020). Altered proximal tubular cell glucose metabolism during acute kidney injury is associated with mortality. Nature Metabolism, 2, 732–743. 10.1038/s42255-020-0238-1 32694833

[phy270203-bib-0043] Lehman, L.‐W. , Saeed, M. , Moody, G. , & Mark, R. (2010). Hypotension as a risk factor for acute kidney injury in ICU patients. Computers in Cardiology, 37, 1095–1098. PMC3120106 22158679

[phy270203-bib-0044] Li, X. , Liu, M. , Bedja, D. , Thoburn, C. , Gabrielson, K. , Racusen, L. , & Rabb, H. (2012). Acute renal venous obstruction is more detrimental to the kidney than arterial occlusion: Implication for murine models of acute kidney injury. American Journal of Physiology. Renal Physiology, 302, F519–F525. 10.1152/ajprenal.00011.2011 22114209 PMC3353642

[phy270203-bib-0045] Liang, J. , & Liu, Y. (2023). Animal models of kidney disease: Challenges and perspectives. Kidney360, 4, 1479–1493. 10.34067/KID.0000000000000227 37526653 PMC10617803

[phy270203-bib-0046] Liu, D. , Shang, H. , & Liu, Y. (2016). Stanniocalcin‐1 protects a mouse model from renal ischemia‐reperfusion injury by affecting ROS‐mediated multiple signaling pathways. International Journal of Molecular Sciences, 17, 1051. 10.3390/ijms17071051 27420048 PMC4964427

[phy270203-bib-0047] Machado, M. N. , Nakazone, M. A. , & Maia, L. N. (2014). Acute kidney injury based on KDIGO (kidney disease improving global outcomes) criteria in patients with elevated baseline serum creatinine undergoing cardiac surgery. Revista Brasileira de Cirurgia Cardiovascular, 29, 299–307. 10.5935/1678-9741.20140049 25372901 PMC4412317

[phy270203-bib-0048] Makris, K. , & Spanou, L. (2016). Acute kidney injury: Definition, pathophysiology and clinical phenotypes. Clinical Biochemist Reviews, 37, 85–98. 28303073 PMC5198510

[phy270203-bib-0049] Malagrino, P. A. , Venturini, G. , Yogi, P. S. , Dariolli, R. , Padilha, K. , Kiers, B. , Gois, T. C. , Da Motta‐Leal‐Filho, J. M. , Takimura, C. K. , Girardi, A. C. C. , Carnevale, F. C. , Zeri, A. C. M. , Malheiros, D. M. A. C. , Krieger, J. E. , & Pereira, A. C. (2014). Catheter‐based induction of renal ischemia/reperfusion in swine: Description of an experimental model. Physiological Reports, 2, e12150. 10.14814/phy2.12150 25263203 PMC4270221

[phy270203-bib-0050] Mishra, J. , Ma, Q. , Prada, A. , Mitsnefes, M. , Zahedi, K. , Yang, J. , Barasch, J. , & Devarajan, P. (2003). Identification of neutrophil gelatinase‐associated lipocalin as a novel early urinary biomarker for ischemic renal injury. Journal of the American Society of Nephrology, 14, 2534–2543. 10.1097/01.ASN.0000088027.54400.C6 14514731

[phy270203-bib-0051] Mitty, H. A. , Shapiro, R. S. , Parsons, R. B. , & Silberzweig, J. E. (1996). Renovascular hypertension. Radiologic Clinics of North America, 34, 1017–1036. 8784394

[phy270203-bib-0052] Molitoris, B. A. (1991). Ischemia‐induced loss of epithelial polarity: Potential role of the actin cytoskeleton. The American Journal of Physiology, 260, F769–F778. 10.1152/ajprenal.1991.260.6.F769 2058700

[phy270203-bib-0053] Moriyama, T. , Hagihara, S. , Shiramomo, T. , Nagaoka, M. , Iwakawa, S. , & Kanmura, Y. (2016). Comparison of three early biomarkers for acute kidney injury after cardiac surgery under cardiopulmonary bypass. Journal of Intensive Care, 4, 41. 10.1186/s40560-016-0164-1 27330813 PMC4915135

[phy270203-bib-0054] Musk, G. C. (2015). Anaesthetising pigs. The Veterinary Record, 177, 96–97. 10.1136/vr.h3880 26206969

[phy270203-bib-0055] Nemours, S. , Castro, L. , Ribatallada‐Soriano, D. , Semidey, M. E. , Aranda, M. , Ferrer, M. , Sanchez, A. , Morote, J. , Cantero‐Recasens, G. , & Meseguer, A. (2022). Temporal and sex‐dependent gene expression patterns in a renal ischemia–reperfusion injury and recovery pig model. Scientific Reports, 12, 6926. 10.1038/s41598-022-10352-3 35484379 PMC9051203

[phy270203-bib-0056] Orvieto, M. A. , Zorn, K. C. , Mendiola, F. , Lyon, M. B. , Mikhail, A. A. , Gofrit, O. N. , & Shalhav, A. L. (2007). Recovery of renal function after complete renal hilar Versus artery alone clamping during open and laparoscopic surgery. The Journal of Urology, 177, 2371–2374. 10.1016/j.juro.2007.01.115 17509361

[phy270203-bib-0057] Ostermann, M. , & Liu, K. (2017). Pathophysiology of AKI. Best Practice & Research. Clinical Anaesthesiology, 31, 305–314. 10.1016/j.bpa.2017.09.001 29248138

[phy270203-bib-0058] Owji, S. M. , Nikeghbal, E. , & Moosavi, S. M. (2018). Comparison of ischaemia–reperfusion‐induced acute kidney injury by clamping renal arteries, veins or pedicles in anaesthetized rats. Experimental Physiology, 103, 1390–1402. 10.1113/EP087140 30091805

[phy270203-bib-0059] Packialakshmi, B. , Stewart, I. J. , Burmeister, D. M. , Chung, K. K. , & Zhou, X. (2020). Large animal models for translational research in acute kidney injury. Renal Failure, 42, 1042–1058. 10.1080/0886022X.2020.1830108 33043785 PMC7586719

[phy270203-bib-0060] Parekh, D. J. , Weinberg, J. M. , Ercole, B. , Torkko, K. C. , Hilton, W. , Bennett, M. , Devarajan, P. , & Venkatachalam, M. A. (2013). Tolerance of the human kidney to isolated controlled ischemia. Journal of the American Society of Nephrology, 24, 506–517. 10.1681/ASN.2012080786 23411786 PMC3582204

[phy270203-bib-0061] Park, Y. , Hirose, R. , Dang, K. , Xu, F. , Behrends, M. , Tan, V. , Roberts, J. P. , & Niemann, C. U. (2008). Increased severity of renal ischemia‐reperfusion injury with venous clamping compared to arterial clamping in a rat model. Surgery, 143, 243–251. 10.1016/j.surg.2007.07.041 18242341

[phy270203-bib-0062] Parker, L. A. , Krebs, K. , Pan, P. L. , Varner, K. M. , & Hoddinott, K. L. (2023). Treatment and outcome following substantial ketamine overdose in a dog. The Canadian Veterinary Journal, 64, 235–238. 36874544 PMC9979721

[phy270203-bib-0063] Pefanis, A. , Bongoni, A. K. , McRae, J. L. , Salvaris, E. J. , Fisicaro, N. , Murphy, J. M. , Ierino, F. L. , & Cowan, P. J. (2023). Dynamics of necroptosis in kidney ischemia‐reperfusion injury. Frontiers in Immunology, 14, 1251452. 10.3389/fimmu.2023.1251452 38022500 PMC10652410

[phy270203-bib-0064] Pehböck, D. , Dietrich, H. , Klima, G. , Paal, P. , Lindner, K. H. , & Wenzel, V. (2015). Anesthesia in swine: Optimizing a laboratory model to optimize translational research. Anaesthesist, 64, 65–70. 10.1007/s00101-014-2371-2 25384955

[phy270203-bib-0065] Pereira‐Sampaio, M. A. , Favorito, L. A. , & Sampaio, F. J. B. (2004). Pig kidney: Anatomical relationships between the intrarenal arteries and the kidney collecting system. Applied study for urological research and surgical training. The Journal of Urology, 172, 2077–2081. 10.1097/01.ju.0000138085.19352.b5 15540793

[phy270203-bib-0066] Pilarczyk, K. , Carstens, H. , Papathanasiou, M. , Luedike, P. , Koch, A. , Jakob, H. , Kamler, M. , & Pizanis, N. (2020). Prediction of acute kidney injury after left ventricular assist device implantation: Evaluation of clinical risk scores. Artificial Organs, 44, 162–173. 10.1111/aor.13548 31361341

[phy270203-bib-0067] Ronco, C. , Bellomo, R. , & Kellum, J. A. (2019). Acute kidney injury. The Lancet, 394, 1949–1964. 10.1016/S0140-6736(19)32563-2 31777389

[phy270203-bib-0068] Salmasi, V. , Maheshwari, K. , Yang, D. , Mascha, E. J. , Singh, A. , Sessler, D. I. , & Kurz, A. (2017). Relationship between intraoperative hypotension, defined by either reduction from baseline or absolute thresholds, and acute kidney and myocardial injury after noncardiac surgery: A retrospective cohort analysis. Anesthesiology, 126, 47–65. 10.1097/ALN.0000000000001432 27792044

[phy270203-bib-0069] Sato, S. , Kitamura, H. , Ghazizadeh, M. , Adachi, A. , Sasaki, Y. , Ishizaki, M. , Inoue, K. , Wakamatsu, K. , & Sugisaki, Y. (2005). Occurrence of hyaline droplets in renal biopsy specimens: An ultrastructural study. Medical Molecular Morphology, 38, 63–71. 10.1007/s00795-004-0272-1 16158182

[phy270203-bib-0070] Scholz, H. , Boivin, F. J. , Schmidt‐Ott, K. M. , Bachmann, S. , Eckardt, K.‐U. , Scholl, U. I. , & Persson, P. B. (2021). Kidney physiology and susceptibility to acute kidney injury: Implications for renoprotection. Nature Reviews. Nephrology, 17, 335–349. 10.1038/s41581-021-00394-7 33547418

[phy270203-bib-0071] Schrezenmeier, E. V. , Barasch, J. , Budde, K. , Westhoff, T. , & Schmidt‐Ott, K. M. (2017). Biomarkers in acute kidney injury – pathophysiological basis and clinical performance. Acta Physiologica, 219, 556–574. 10.1111/apha.12764 PMC5575831 27474473

[phy270203-bib-0072] Serraino, G. F. , Provenzano, M. , Jiritano, F. , Michael, A. , Ielapi, N. , Mastroroberto, P. , Andreucci, M. , & Serra, R. (2021). Risk factors for acute kidney injury and mortality in high risk patients undergoing cardiac surgery. PLoS One, 16, e0252209. 10.1371/journal.pone.0252209 34019579 PMC8139497

[phy270203-bib-0073] Shang, W. , & Wang, Z. (2017). The update of NGAL in acute kidney injury. Current Protein & Peptide Science, 18, 1211–1217. 10.2174/1389203717666160909125004 27634444

[phy270203-bib-0074] Sharif, S. I. , & Abouazra, H. A. (2009). Effect of intravenous ketamine administration on blood glucose levels in conscious rabbits. American Journal of Pharmacology and Toxicology, 4, 38–45. 10.3844/ajptsp.2009.38.45

[phy270203-bib-0075] Shum, H.‐P. , Kong, H. H.‐Y. , Chan, K.‐C. , Yan, W.‐W. , & Chan, T. M. (2016). Septic acute kidney injury in critically ill patients – A single‐center study on its incidence, clinical characteristics, and outcome predictors. Renal Failure, 38, 706–716. 10.3109/0886022X.2016.1157749 26981621

[phy270203-bib-0076] Siew, E. D. , & Davenport, A. (2015). The growth of acute kidney injury: A rising tide or just closer attention to detail? Kidney International, 87, 46–61. 10.1038/ki.2014.293 25229340 PMC4281297

[phy270203-bib-0077] Silberstein, J. L. , Sprenkle, P. C. , Su, D. , Power, N. E. , Tarin, T. V. , Ezell, P. , Sjoberg, D. D. , Feifer, A. , Fleisher, M. , Russo, P. , & Touijer, K. A. (2013). Neutrophil gelatinase‐associated lipocalin (NGAL) levels in response to unilateral renal ischaemia in a novel pilot two‐kidney porcine model: NGAL response to ischaemia in two‐kidney porcine model. BJU International, 112, 517–525. 10.1111/bju.12066 23510358 PMC4235973

[phy270203-bib-0078] Singbartl, K. , & Kellum, J. A. (2012). AKI in the ICU: Definition, epidemiology, risk stratification, and outcomes. Kidney International, 81, 819–825. 10.1038/ki.2011.339 21975865

[phy270203-bib-0079] Skaanild, M. T. , & Friis, C. (2008). Analyses of CYP2C in porcine microsomes. Basic & Clinical Pharmacology & Toxicology, 103, 487–492. 10.1111/j.1742-7843.2008.00323.x 18803635

[phy270203-bib-0080] Slocum, J. L. , Heung, M. , & Pennathur, S. (2012). Marking renal injury: Can we move beyond serum creatinine? Translational Research, 159, 277–289. 10.1016/j.trsl.2012.01.014 22424431 PMC3308350

[phy270203-bib-0081] Sølling, C. , Christensen, A. T. , Krag, S. , Frøkiaer, J. , Wogensen, L. , Krog, J. , & Tønnesen, E. K. (2011). Erythropoietin administration is associated with short‐term improvement in glomerular filtration rate after ischemia‐reperfusion injury: EPO on renal ischemia‐reperfusion. Acta Anaesthesiologica Scandinavica, 55, 185–195. 10.1111/j.1399-6576.2010.02369.x 21226860

[phy270203-bib-0082] Sun, L. Y. , Wijeysundera, D. N. , Tait, G. A. , & Beattie, W. S. (2015). Association of Intraoperative Hypotension with acute kidney injury after elective noncardiac surgery. Anesthesiology, 123, 515–523. 10.1097/ALN.0000000000000765 26181335

[phy270203-bib-0083] Supavekin, S. , Zhang, W. , Kucherlapati, R. , Kaskel, F. J. , Moore, L. C. , & Devarajan, P. (2003). Differential gene expression following early renal ischemia/reperfusion. Kidney International, 63, 1714–1724. 10.1046/j.1523-1755.2003.00928.x 12675847

[phy270203-bib-0084] Thadhani, R. , Pascual, M. , & Bonventre, J. V. (1996). Acute renal failure. The New England Journal of Medicine, 334, 1448–1460. 10.1056/NEJM199605303342207 8618585

[phy270203-bib-0085] Thiele, R. H. , Isbell, J. M. , & Rosner, M. H. (2015). AKI associated with cardiac surgery. Clinical Journal of the American Society of Nephrology, 10, 500–514. 10.2215/CJN.07830814 25376763 PMC4348689

[phy270203-bib-0086] Varughese, S. , & Ahmed, R. (2021). Environmental and occupational considerations of anesthesia: A narrative review and update. Anesthesia and Analgesia, 133, 826–835. 10.1213/ANE.0000000000005504 33857027 PMC8415729

[phy270203-bib-0087] Virzì, G. M. , Clementi, A. , De Cal, M. , Cruz, D. N. , & Ronco, C. (2013). Genomics and biological activity of neutrophil gelatinase‐associated lipocalin in several clinical settings. Blood Purification, 35, 139–143. 10.1159/000346100 23343559

[phy270203-bib-0088] Waikar, S. S. , Betensky, R. A. , & Bonventre, J. V. (2009). Creatinine as the gold standard for kidney injury biomarker studies? Nephrology, Dialysis, Transplantation, 24, 3263–3265. 10.1093/ndt/gfp428 19736243

[phy270203-bib-0089] Walsh, M. , Devereaux, P. J. , Garg, A. X. , Kurz, A. , Turan, A. , Rodseth, R. N. , Cywinski, J. , Thabane, L. , & Sessler, D. I. (2013). Relationship between intraoperative mean arterial pressure and clinical outcomes after noncardiac surgery. Anesthesiology, 119, 507–515. 10.1097/ALN.0b013e3182a10e26 23835589

[phy270203-bib-0090] Wang, S. , Wang, J.‐Y. , Wang, T. , Hang, C.‐C. , Shao, R. , & Li, C.‐S. (2017). A novel porcine model of septic shock induced by acute respiratory distress syndrome due to methicillin‐resistant Staphylococcus aureus. Chinese Medical Journal, 130, 1226–1235. 10.4103/0366-6999.205854 28485324 PMC5443030

[phy270203-bib-0091] Wei, Q. , & Dong, Z. (2012). Mouse model of ischemic acute kidney injury: Technical notes and tricks. American Journal of Physiology. Renal Physiology, 303, F1487–F1494. 10.1152/ajprenal.00352.2012 22993069 PMC3532486

[phy270203-bib-0092] Wen, L. , Li, Y. , Li, S. , Hu, X. , Wei, Q. , & Dong, Z. (2021). Glucose metabolism in acute kidney injury and kidney repair. Frontiers in Medicine, 8, 744122. 10.3389/fmed.2021.744122 34912819 PMC8666949

[phy270203-bib-0093] Wickham, H. (2009). ggplot2: Elegant Graphics for Data Analysis. Springer. 10.1007/978-0-387-98141-3

[phy270203-bib-0094] Wong, B. , St. Onge, J. , Korkola, S. , & Prasad, B. (2015). Validating a scoring tool to predict acute kidney injury (AKI) following cardiac surgery. Canadian Journal of Kidney Health and Disease, 2, 37. 10.1186/s40697-015-0037-x 25780626 PMC4349478

