# Supplementary material for: Temporary bilateral clamping of renal arteries induces ischemia–reperfusion: A new pig model of acute kidney injury using total intravenous anesthesia
Source: Physiol Rep. 2025 Feb 2;13(3):e70203. doi: 10.14814/phy2.70203 (PMC11788332; doi:10.14814/phy2.70203)
Supplement: Supplementary file 1 — Figure S1. [file PHY2-13-e70203-s001.docx]

**Supplementary data file**

**Temporary bilateral clamping of renal arteries induces ischemia-reperfusion: a new pig model of acute kidney injury using total intravenous anesthesia**

Axel Guilpin^1,2^, Mathieu Magnin^2,3^, Axel Aigle^1^, Jean-Yves Ayoub^2^, Timothée Schuhler^2^, Romain Lac^2^, Thierry Marchal^4^, Thomas Brichart^1^, Abdessalem Hammed^2^, Vanessa Louzier^2,3*^

^1^ MexBrain, 13 Avenue Albert Einstein, 69100 Villeurbanne, France

^2^ Université de Lyon, UR APCSe Agressions Pulmonaires et Circulatoires dans le Sepsis, VetAgro Sup, 1 avenue Bourgelat F-69280 Marcy l’Etoile, France

^3^ Université de Lyon, VetAgro Sup, Unité de Physiologie, Pharmacodynamie et Thérapeutique, 1 avenue Bourgelat F-69280 Marcy l’Etoile, France

^4^ Université de Lyon, VetAgro Sup, Interactions Cells Environment, UPSP 2016.A104, 69280 Marcy l’Etoile, France

**Figure S1a – Surgery procedure: dissection of muscles to access to kidney**

**
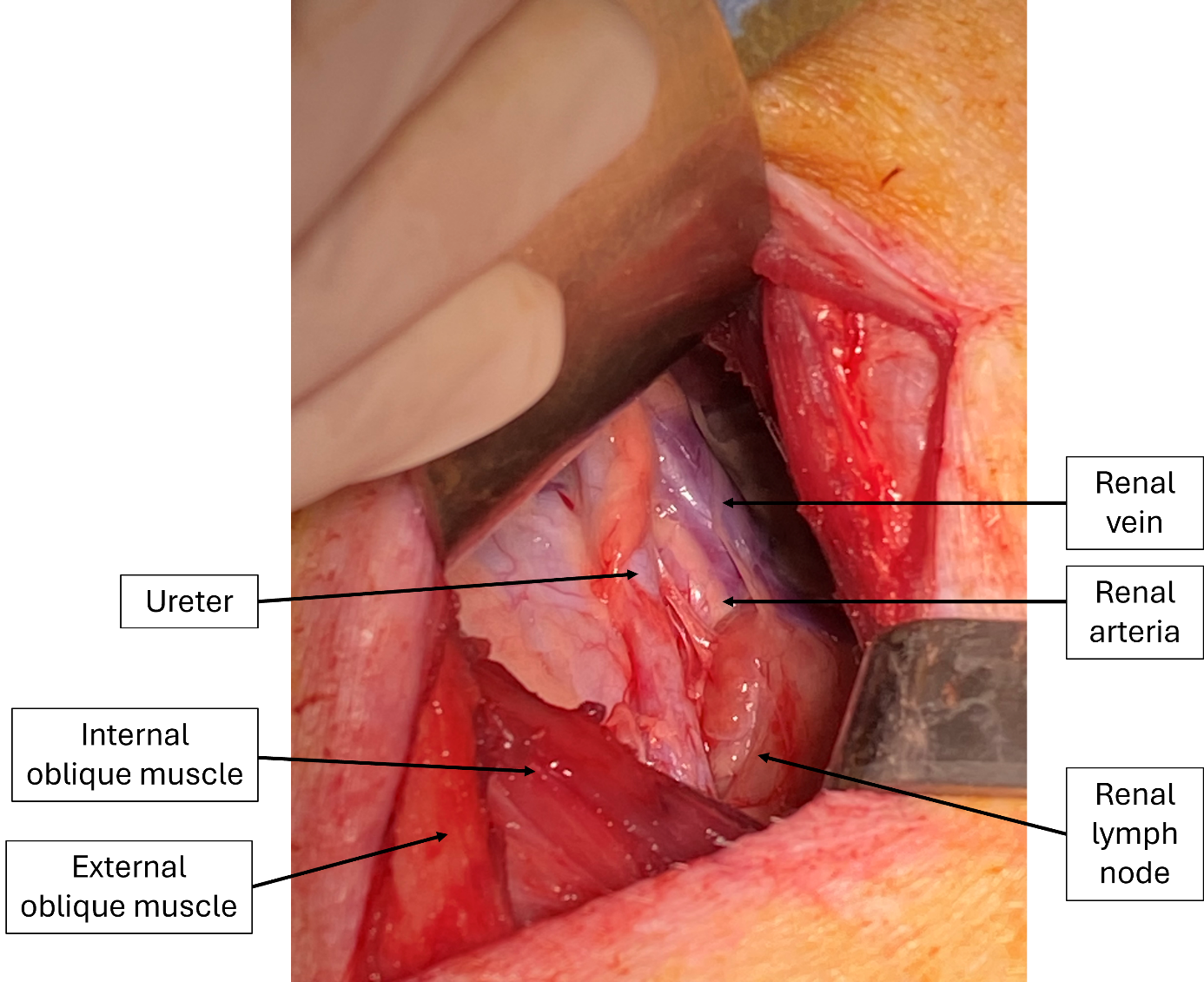
**

**Figure S1b – Surgery procedure: isolation of kidney arteria**

**
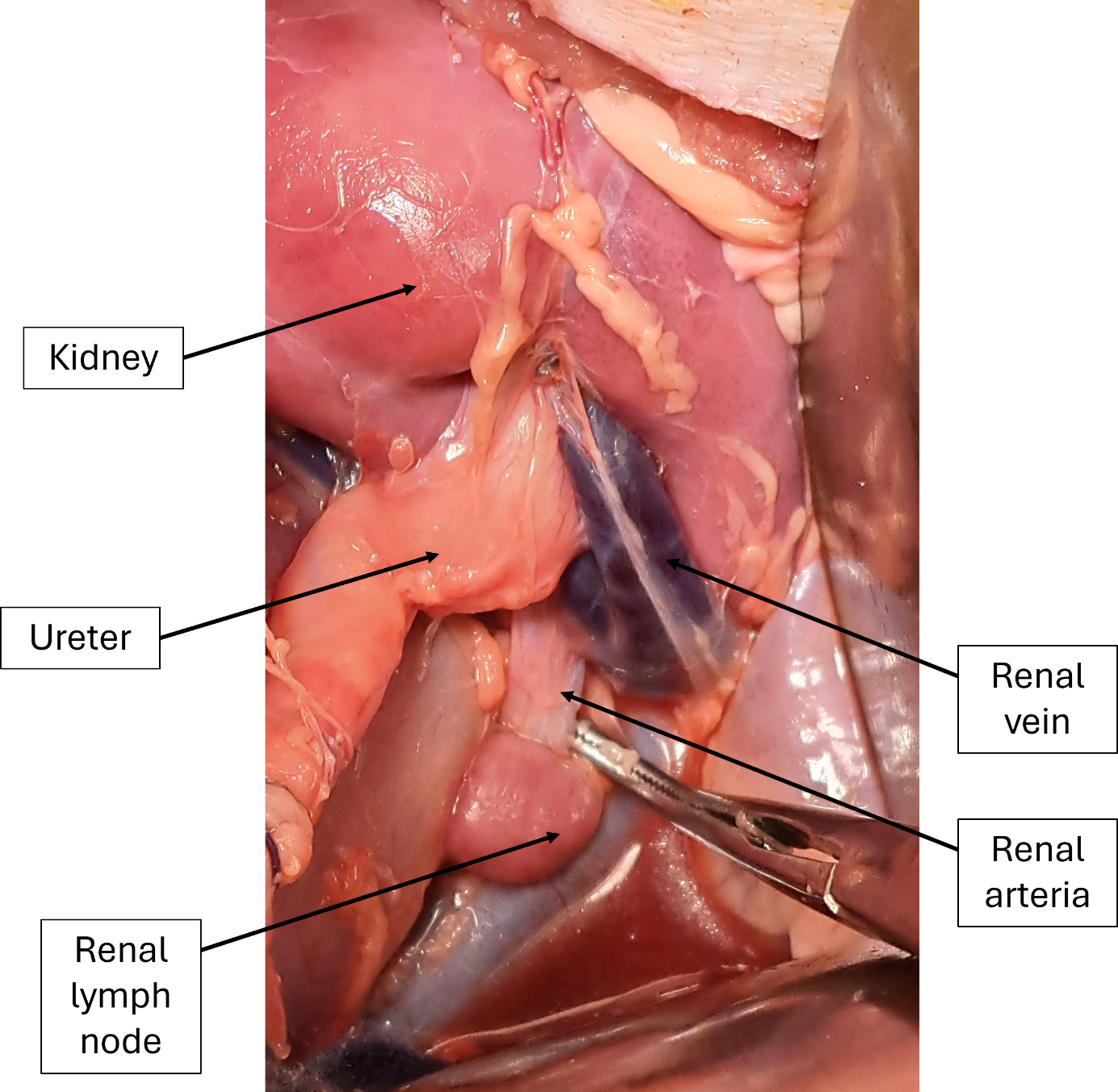
**

**Figure S2a – Evolution of glomerular filtration rate**

**
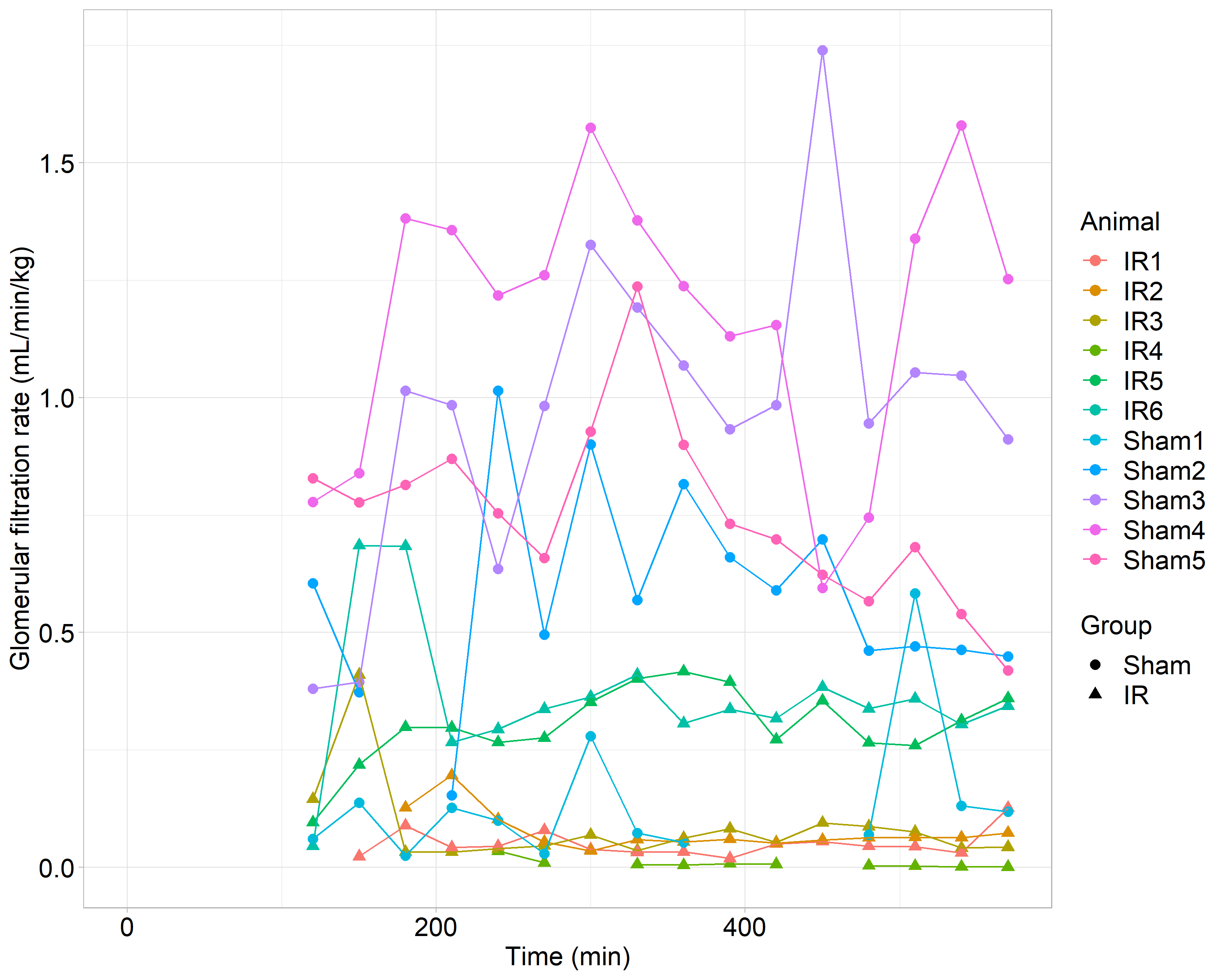
**

**Figure S2b – Evolution of diuresis**

**
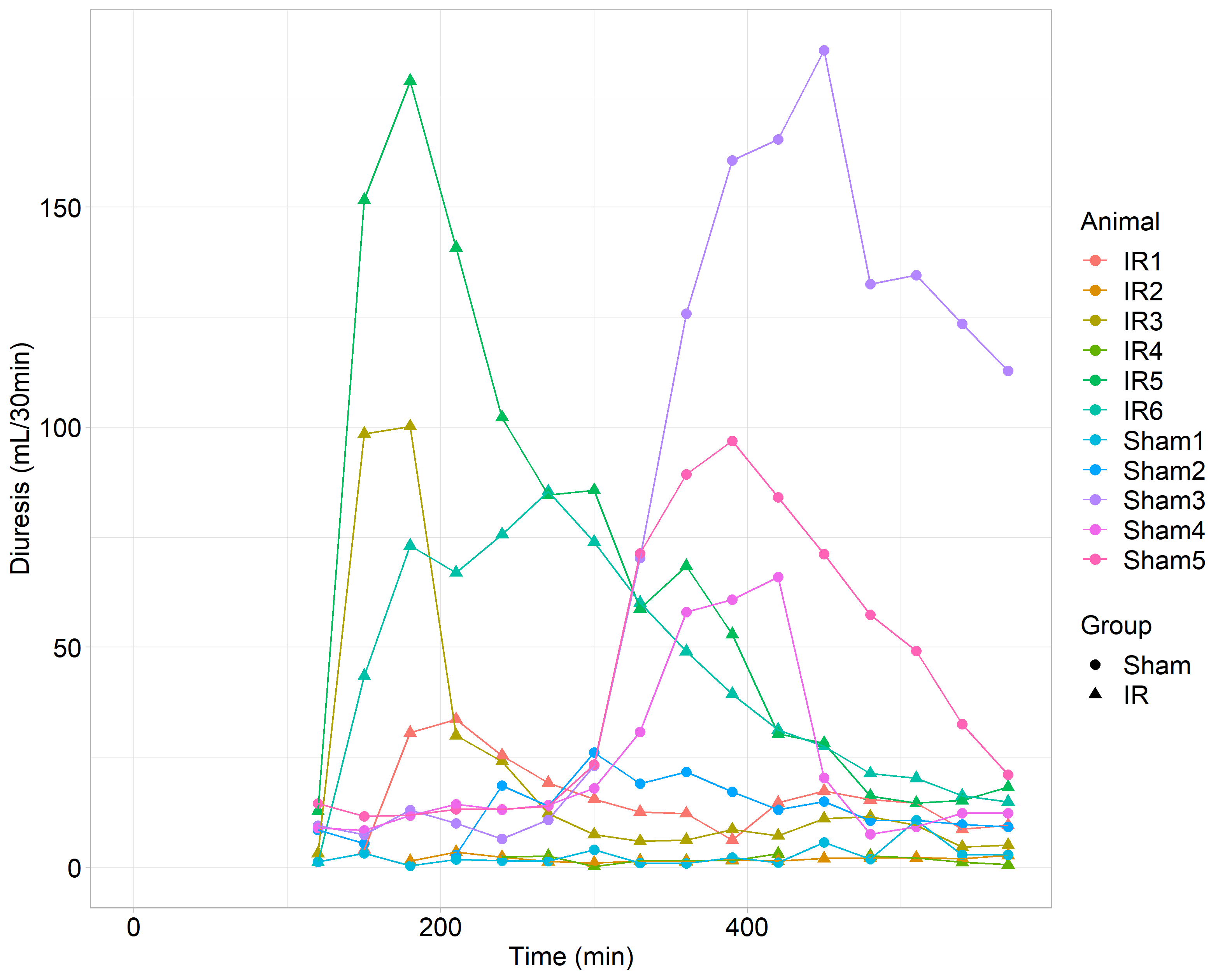
**

**Figure S2c – Diuresis** **for both group (“IR” group (N = 6) and “Sham” group (N = 5)). At each timepoint, an analysis using Kruskall-Wallis test was performed.**

**
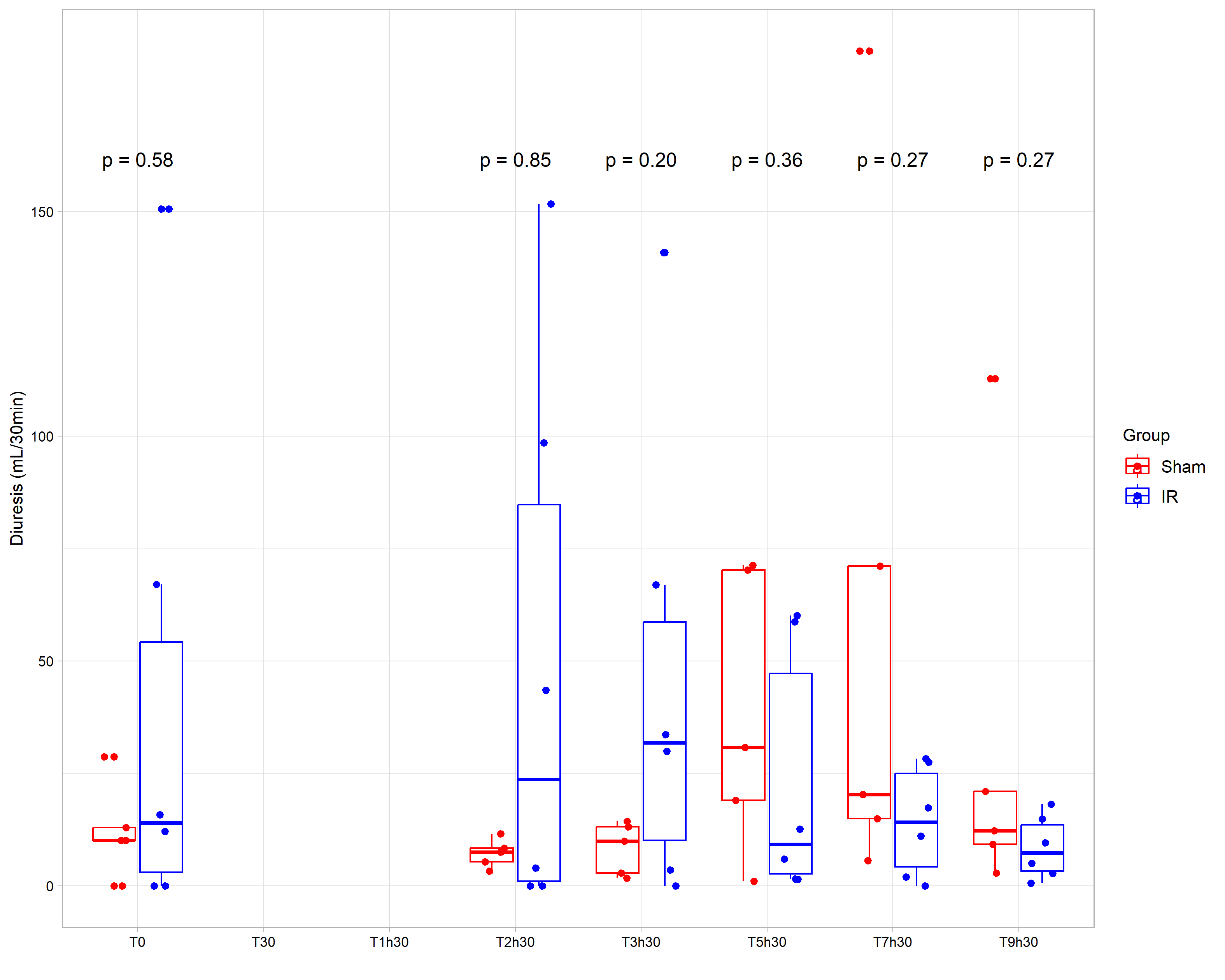
**

**Figure S2d – Evolution of plasma potassium**

**
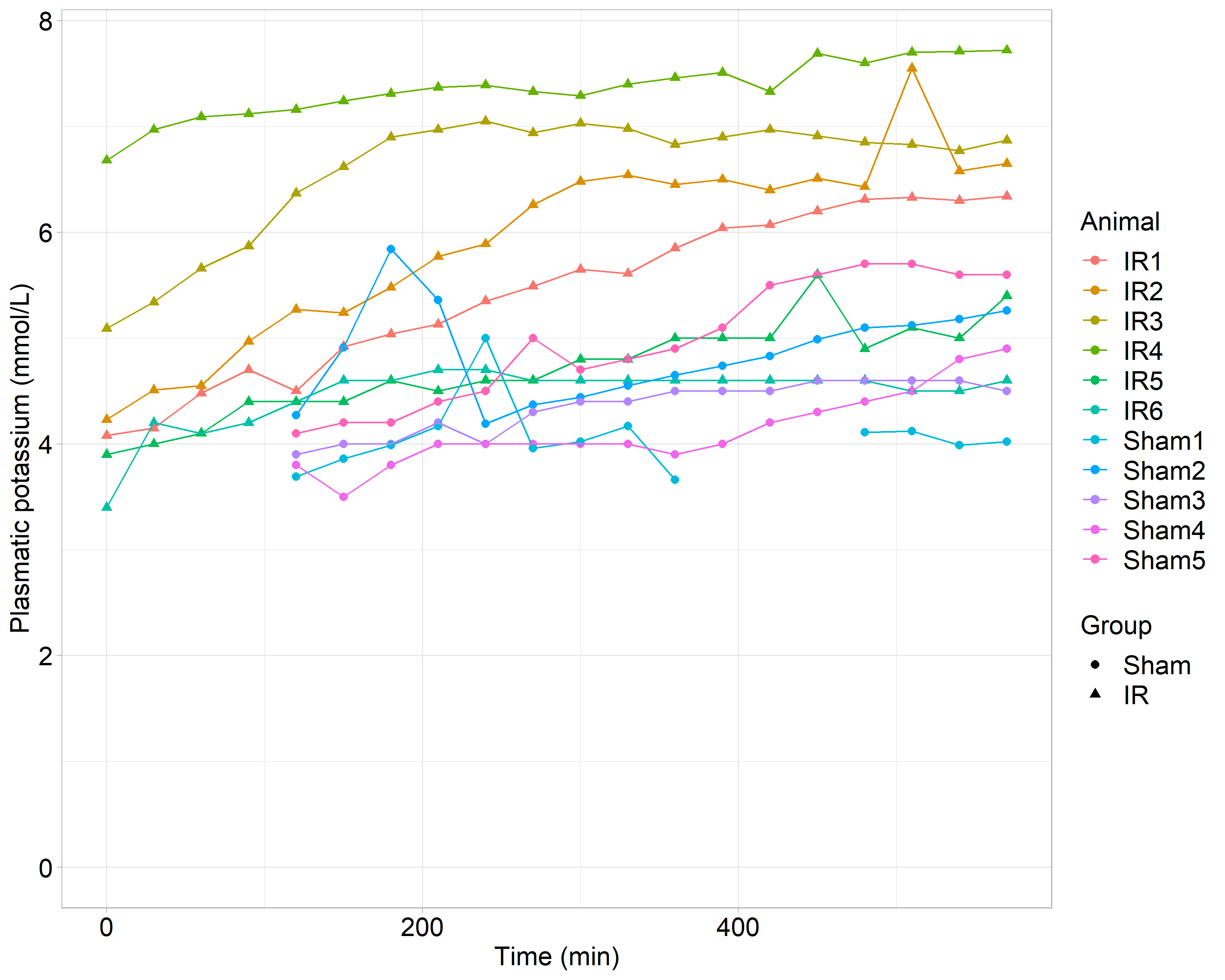
**

**Figure S2e – Comparison of plasma potassium’s evolution during reperfusion: details of linear mixed model**

The table below shows the results of the models. Models are expressed as:

Plasma potassium ~ Group + Time + Group:Time + (1|Animal)`

Time, Group and interaction between group and time were assimilated as a fixed effect. Animal was assimilated as a random effect.

| **Response variable** | **Group** | **Estimate** | **CI95%** | **P** |
| --- | --- | --- | --- | --- |
| Plasma potassium | Sham | + 0.002 mmol/L | 0.0013 mmol/L, 0.0022 mmol/L | < 0.0001 |
|  | IR | + 0.003 mmol/L | 0.0016 mmol/L, 0.0037 mmol/L | 0.003 |

**Figure S3a – Evolution of plasma creatinine**

**
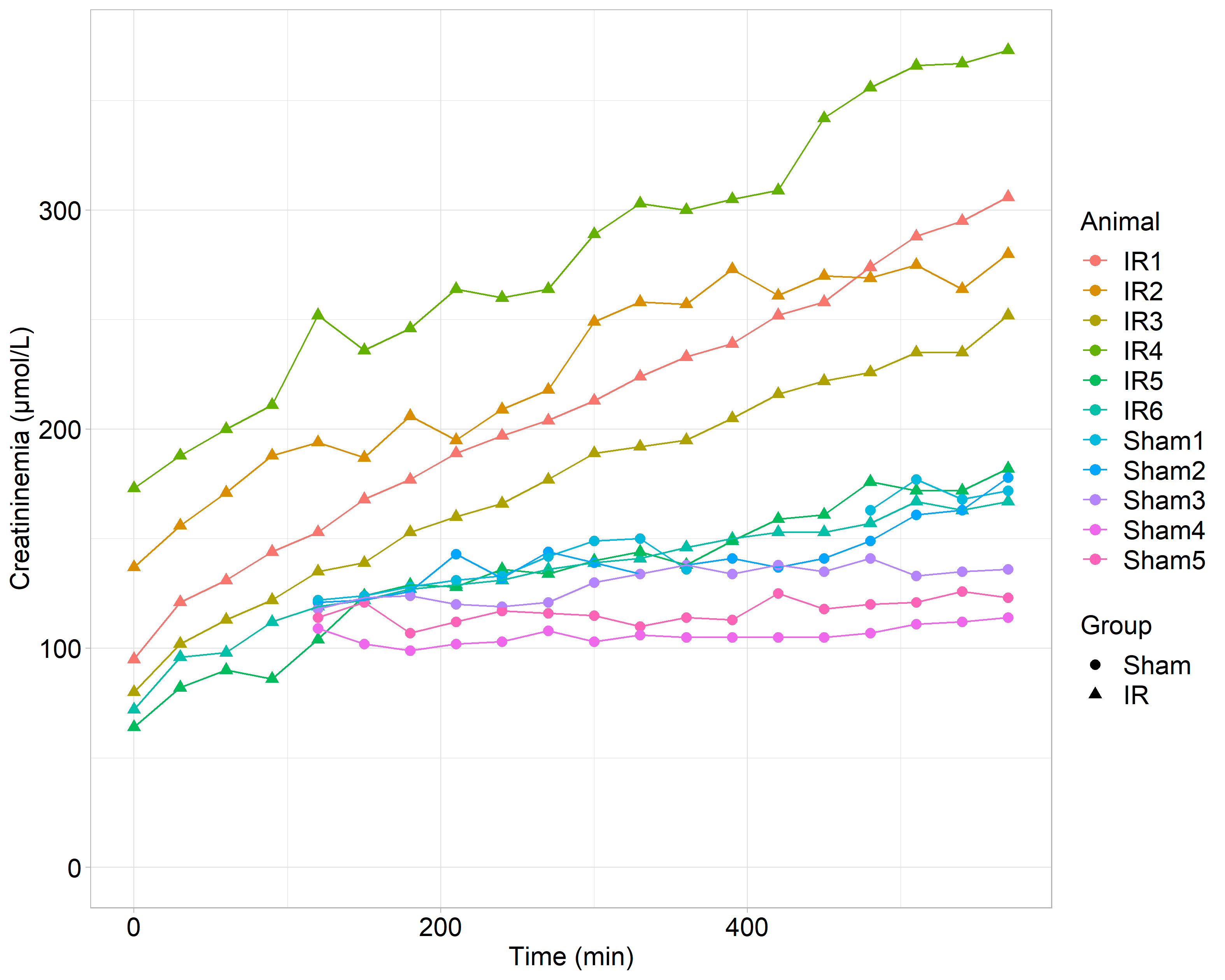
**

**Figure S3b – Comparison of plasma creatinine’s evolution during reperfusion: details of linear mixed model**

The table below shows the results of the models. Models are expressed as:

Plasma creatinine ~ Group + Time + Group:Time + (1|Animal)`

Time, Group and interaction between group and time were assimilated as a fixed effect. Animal was assimilated as a random effect.

| **Response variable** | **Group** | **Estimate** | **CI95%** | **P** |
| --- | --- | --- | --- | --- |
| Plasma creatinine | Sham | + 0.059 µmol/L | 0.039 µmol/L ; 0.076 µmol/L | < 0.0001 |
|  | IR | + 0.253 µmol/L | 0.211 µmol/L ; 0.294 µmol/L | < 0.0001 |

**Figure S4a – Evolution of plasma urea**

**
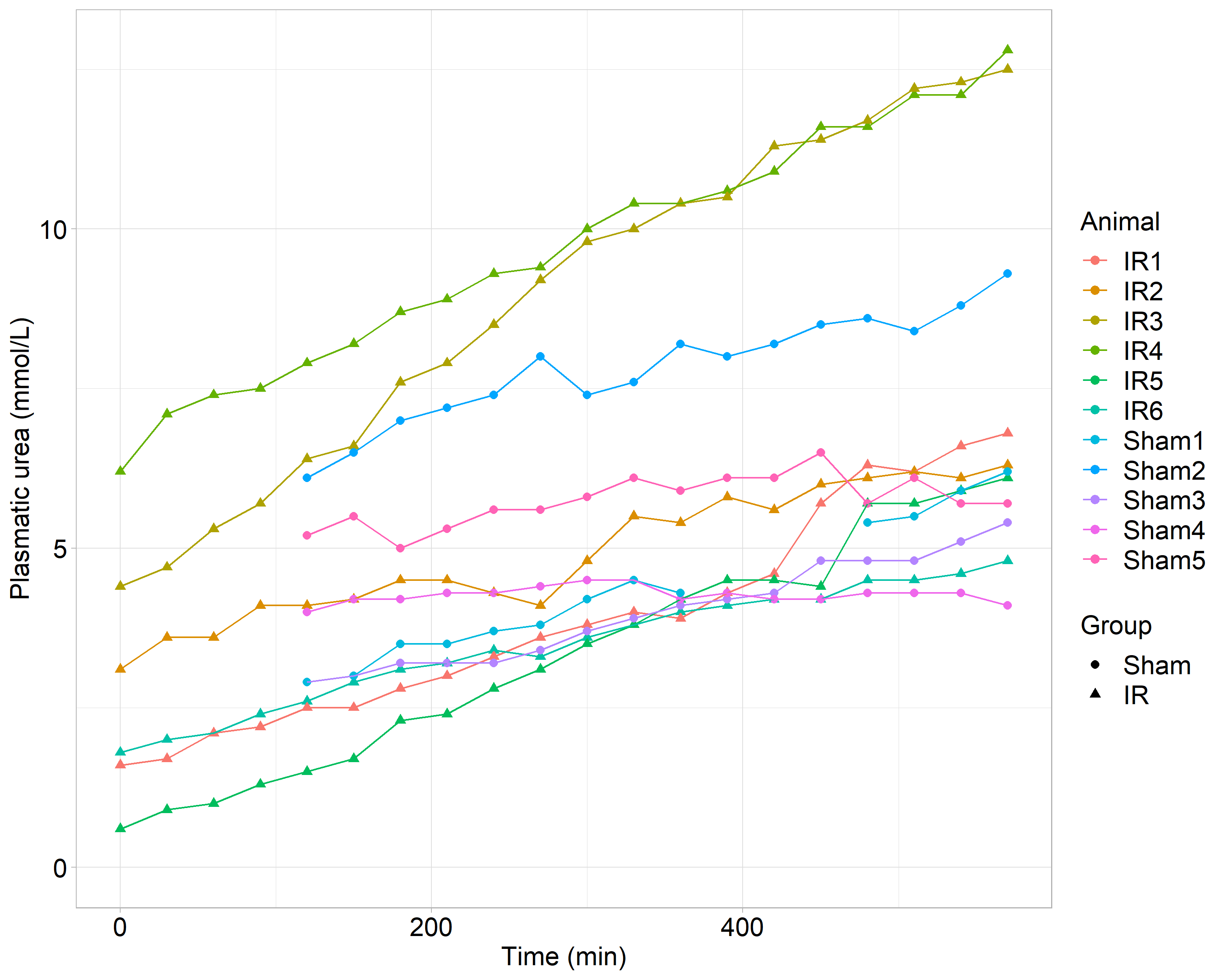
**

**Figure S4b – Comparison of plasma urea’s evolution during reperfusion: details of linear mixed model**

The table below shows the results of the models. Models are expressed as:

Plasma urea ~ Group + Time + Group:Time + (1|Animal)`

Time, Group and interaction between group and time were assimilated as a fixed effect. Animal was assimilated as a random effect.

| **Response variable** | **Group** | **Estimate** | **CI95%** | **P** |
| --- | --- | --- | --- | --- |
| Plasma urea | Sham | + 0.004 mmol/L | 0.003 mmol/L, 0.005 mmol/L | < 0.0001 |
|  | IR | + 0.009 mmol/L | 0.008 mmol/L, 0.011 mmol/L | < 0.0001 |

**Figure S5a – Evolution of aspartate aminotransferase (ASAT)**

**
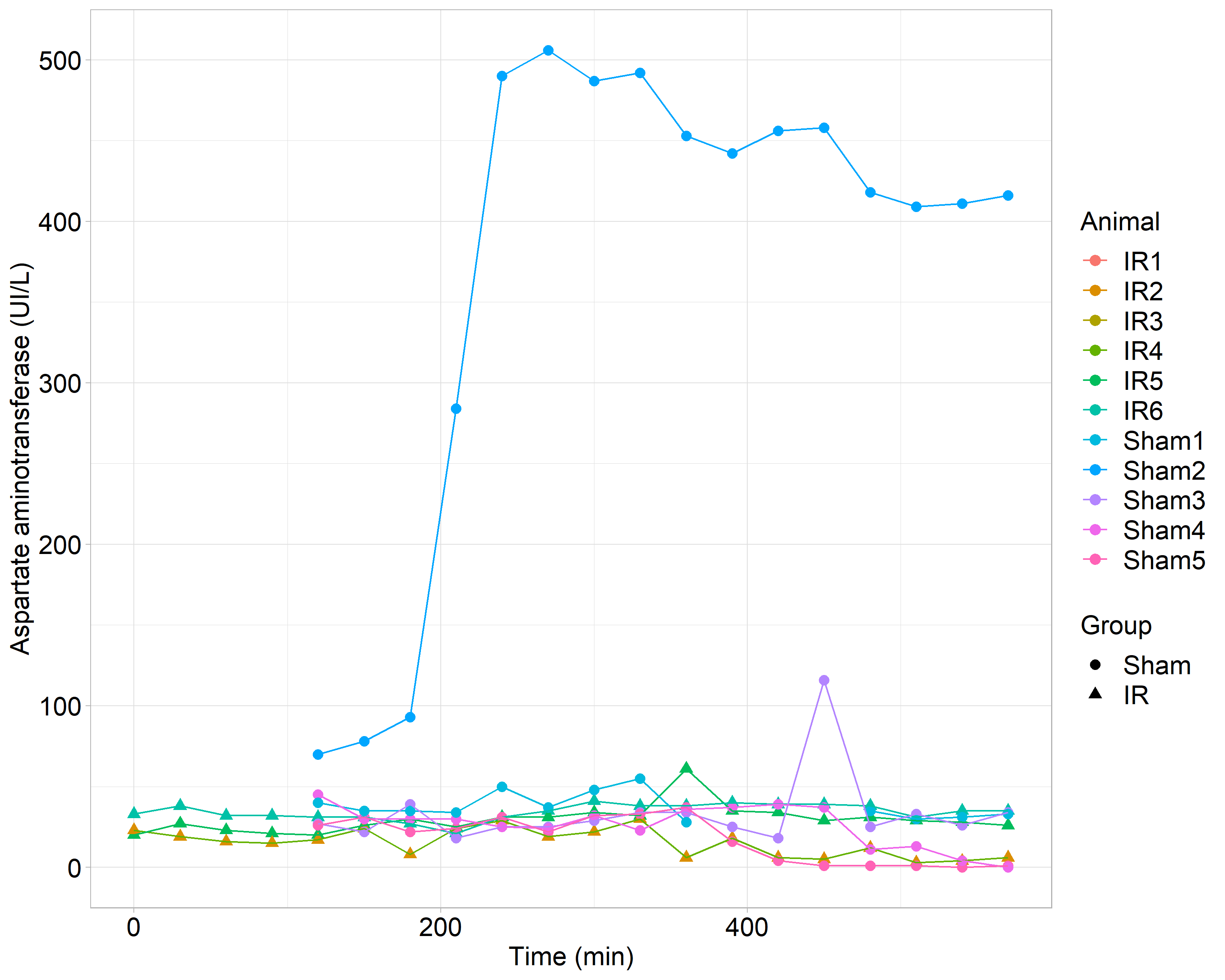
**

**Figure S5b – Evolution of alanine aminotransferase (ALAT)**

**
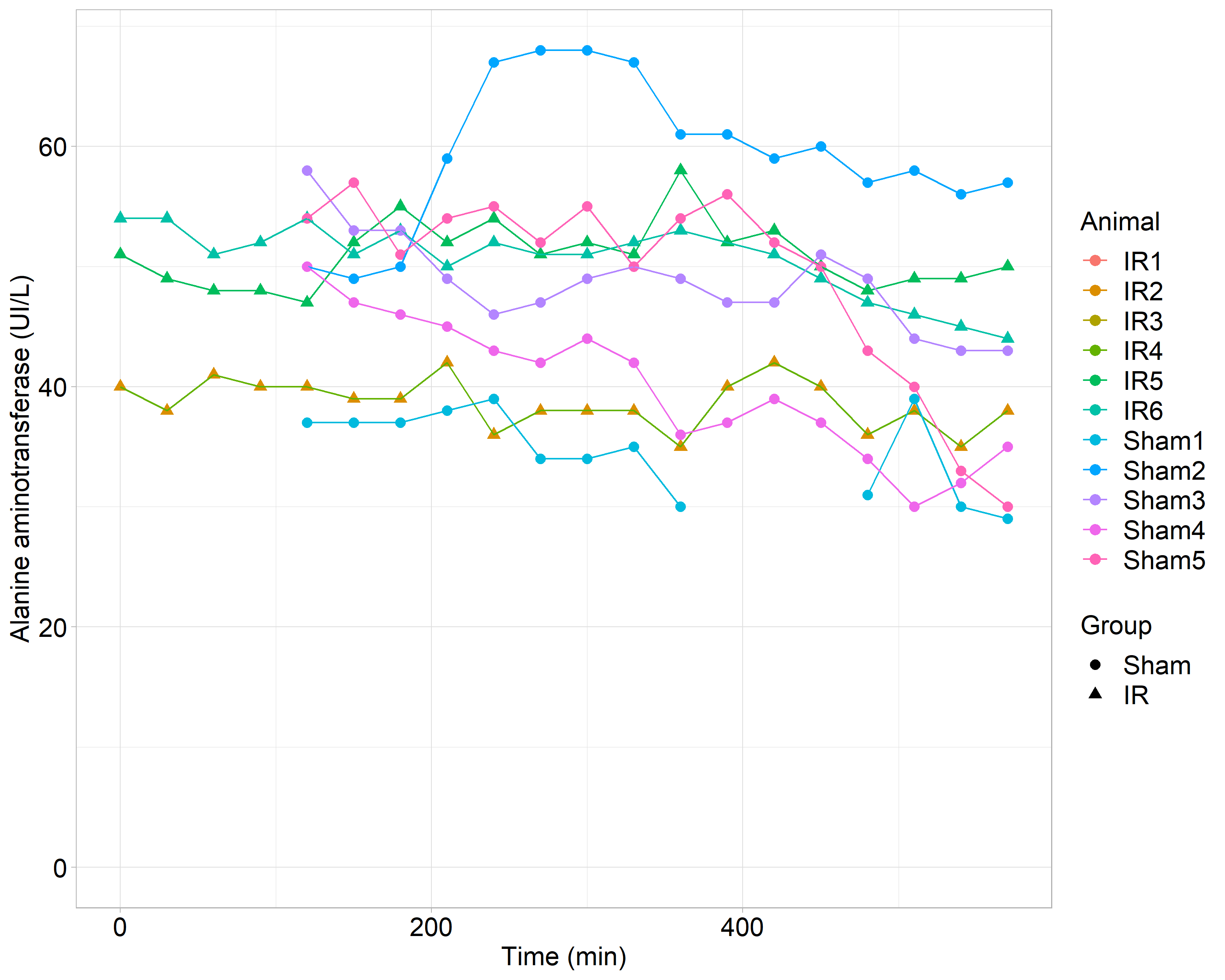
**

**Figure S6a – Evolution of plasma hemoglobin**

**
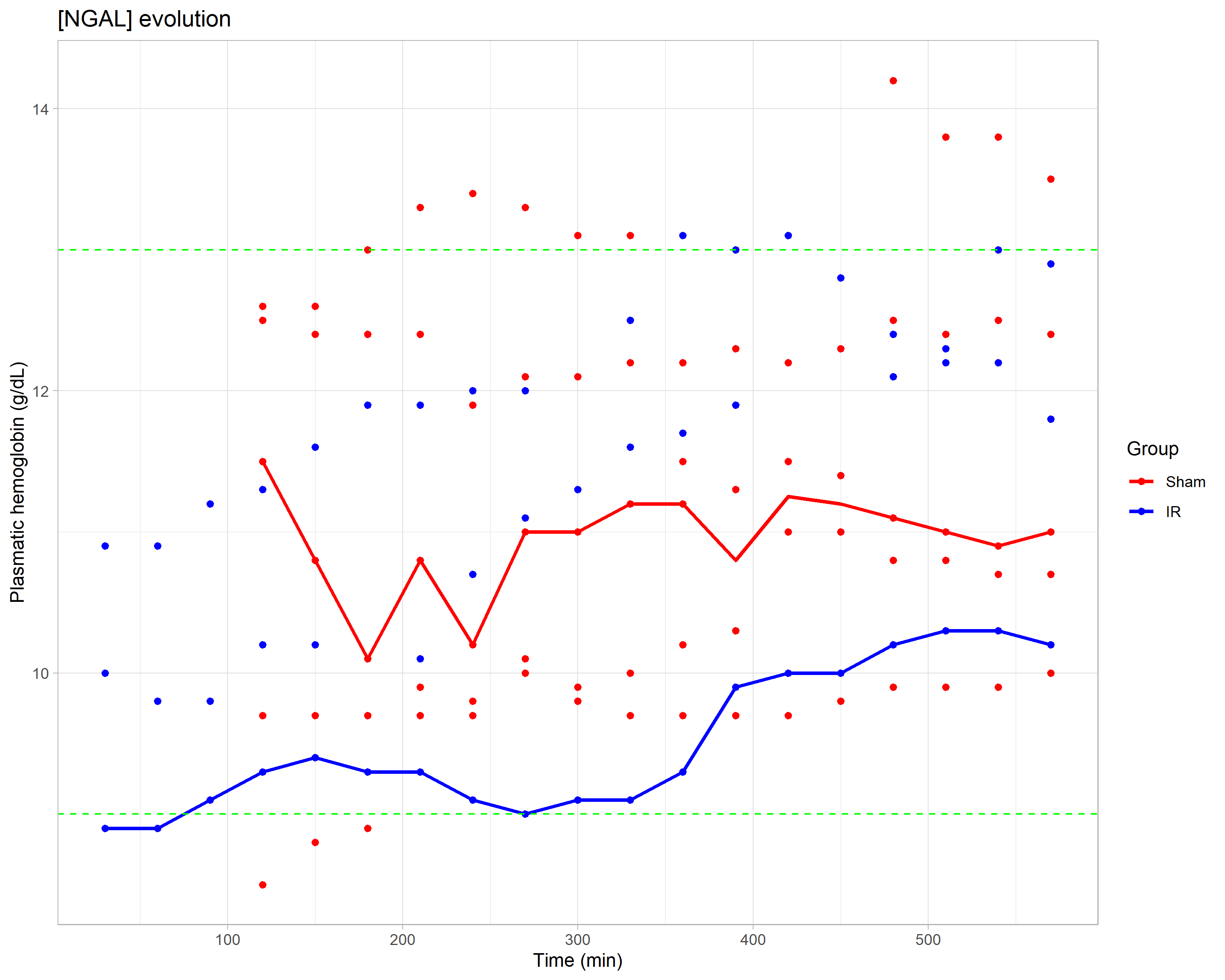
**

Green dashed line represents physiological range of pig’s plasma hemoglobin

**Figure S6b – Evolution of total plasma protein**

**
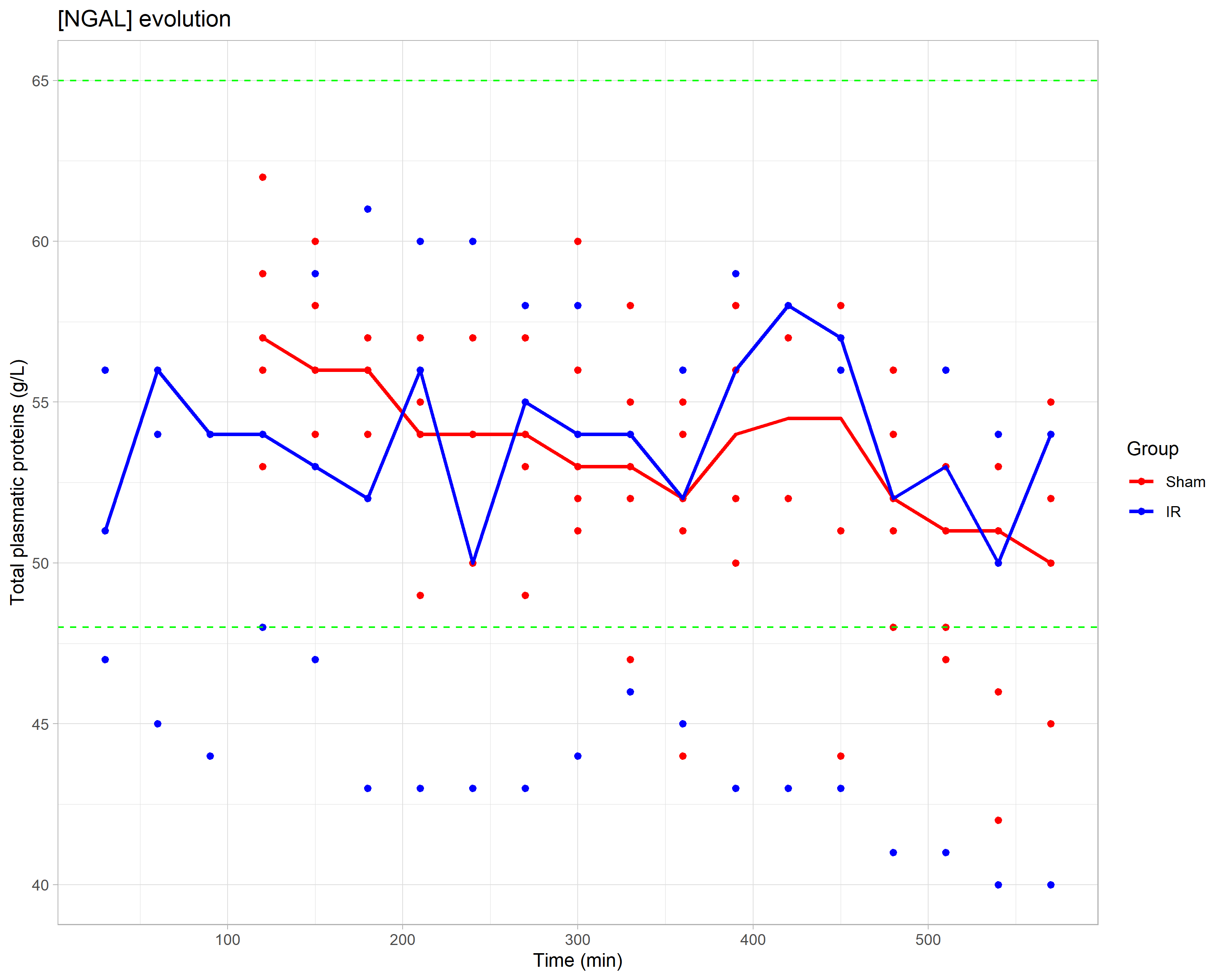
**

Green dashed line represents physiological range of pig’s total plasma proteins

**Figure S7 – Evolution of protein to creatinine ratio in urine**

**
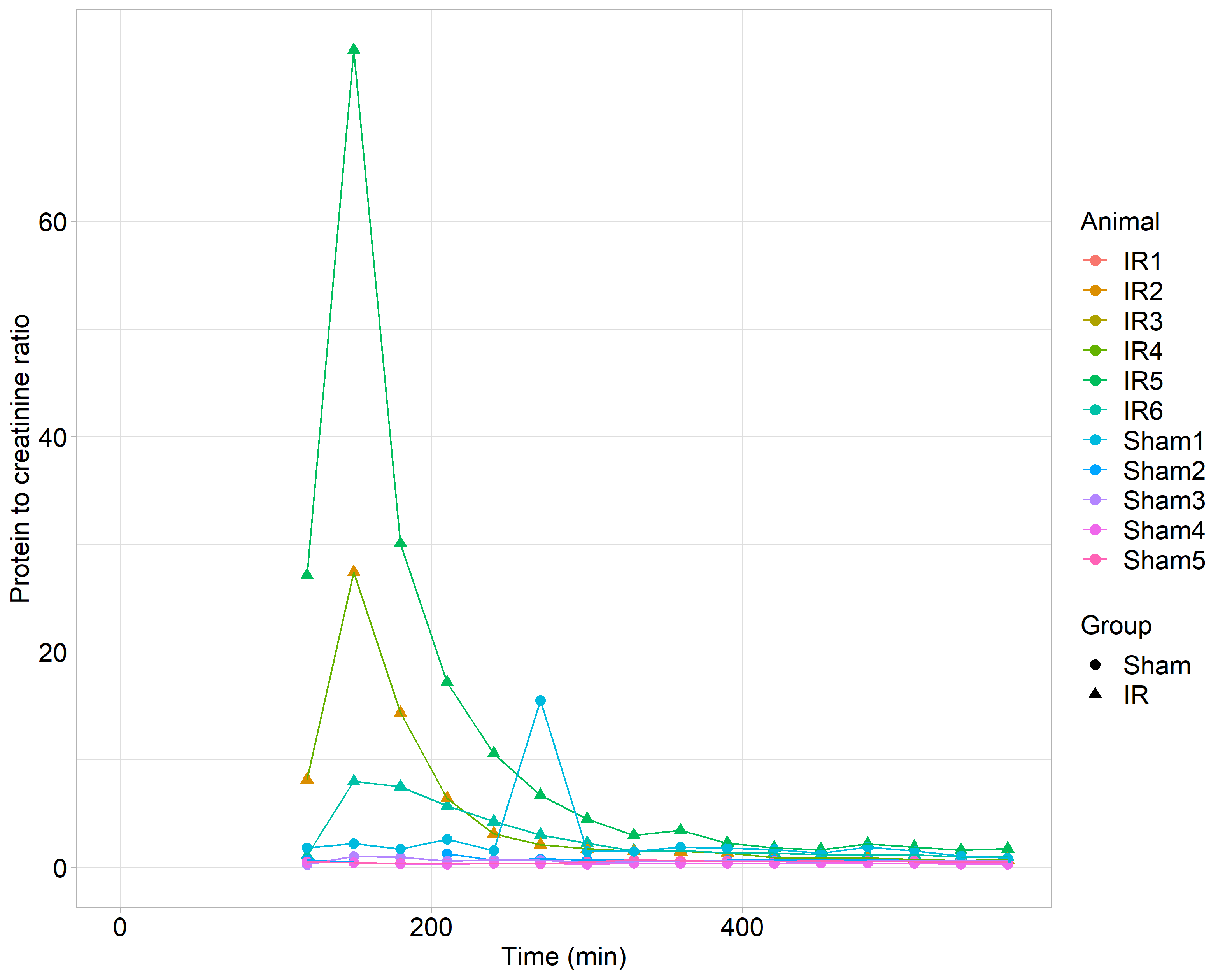
**

**Figure S8 – Evolution of urinary NGAL**

**
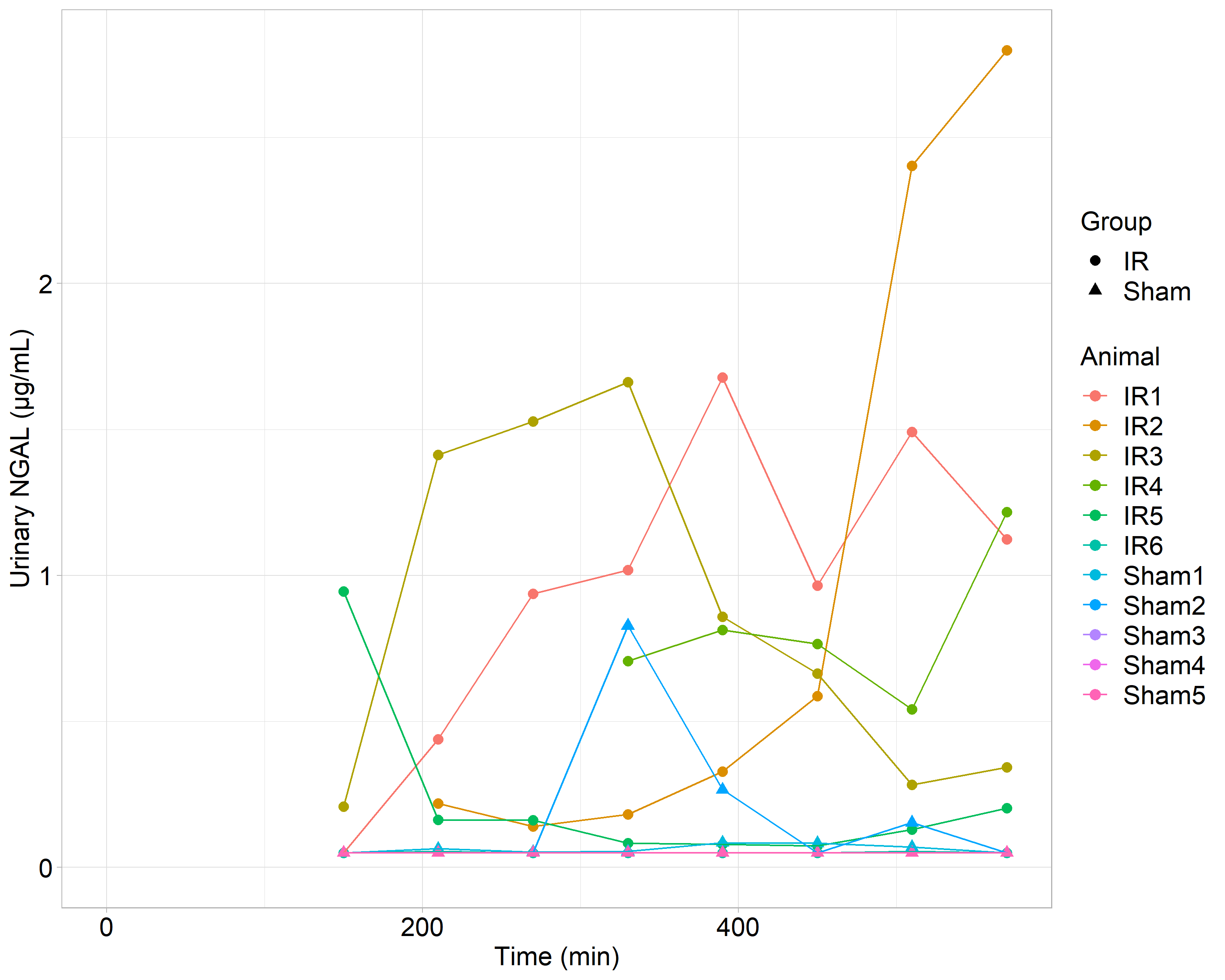
**

NGAL: neutrophil gelatinase associated lipocalin

**Figure S9a – Evolution of mean arterial pressure**

**
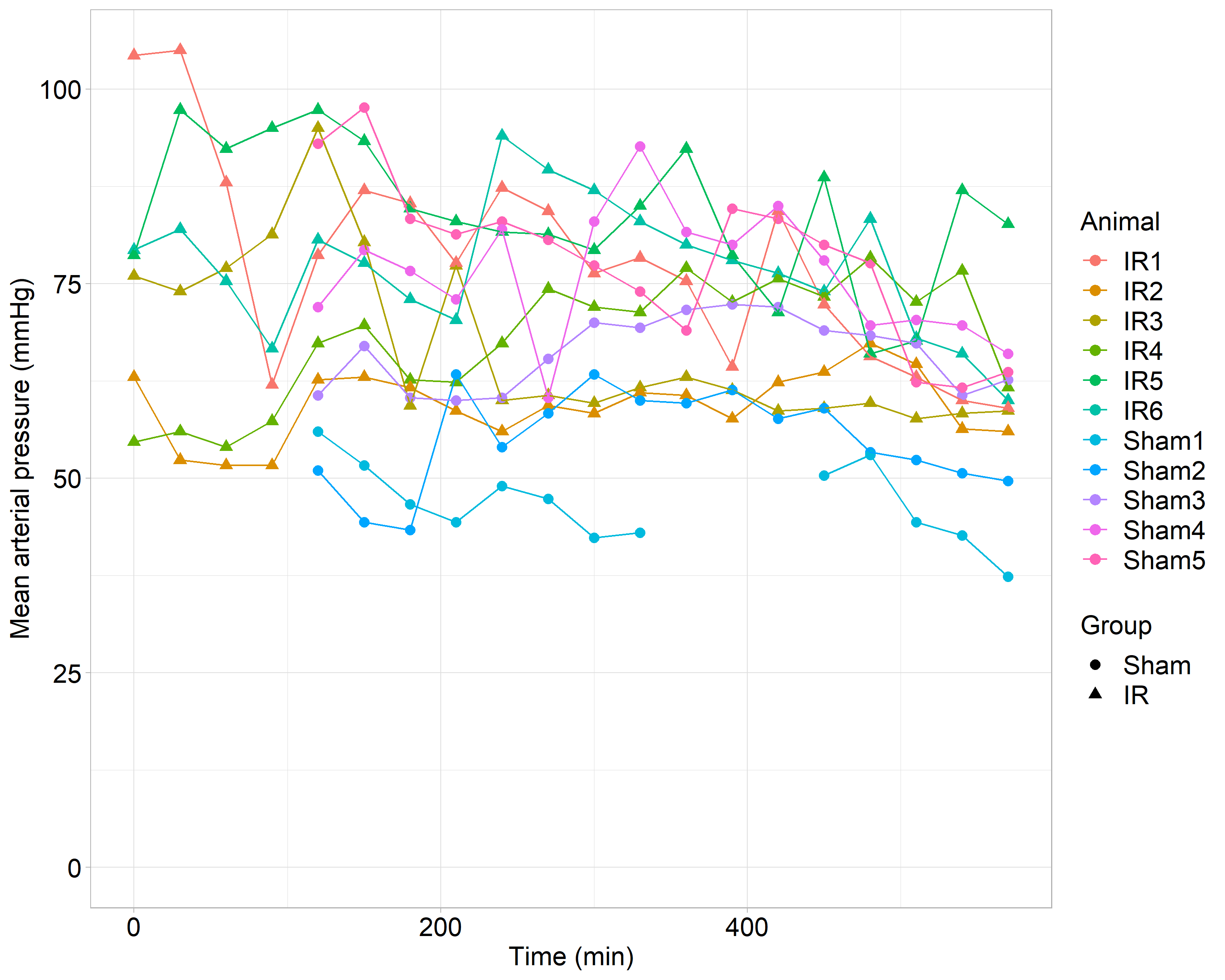
**

**Figure S9b – Mean arterial pressure for both group (“IR” group (N = 6) and “Sham” group (N = 5)). At each timepoint, an analysis using Kruskall-Wallis test was performed.**

**
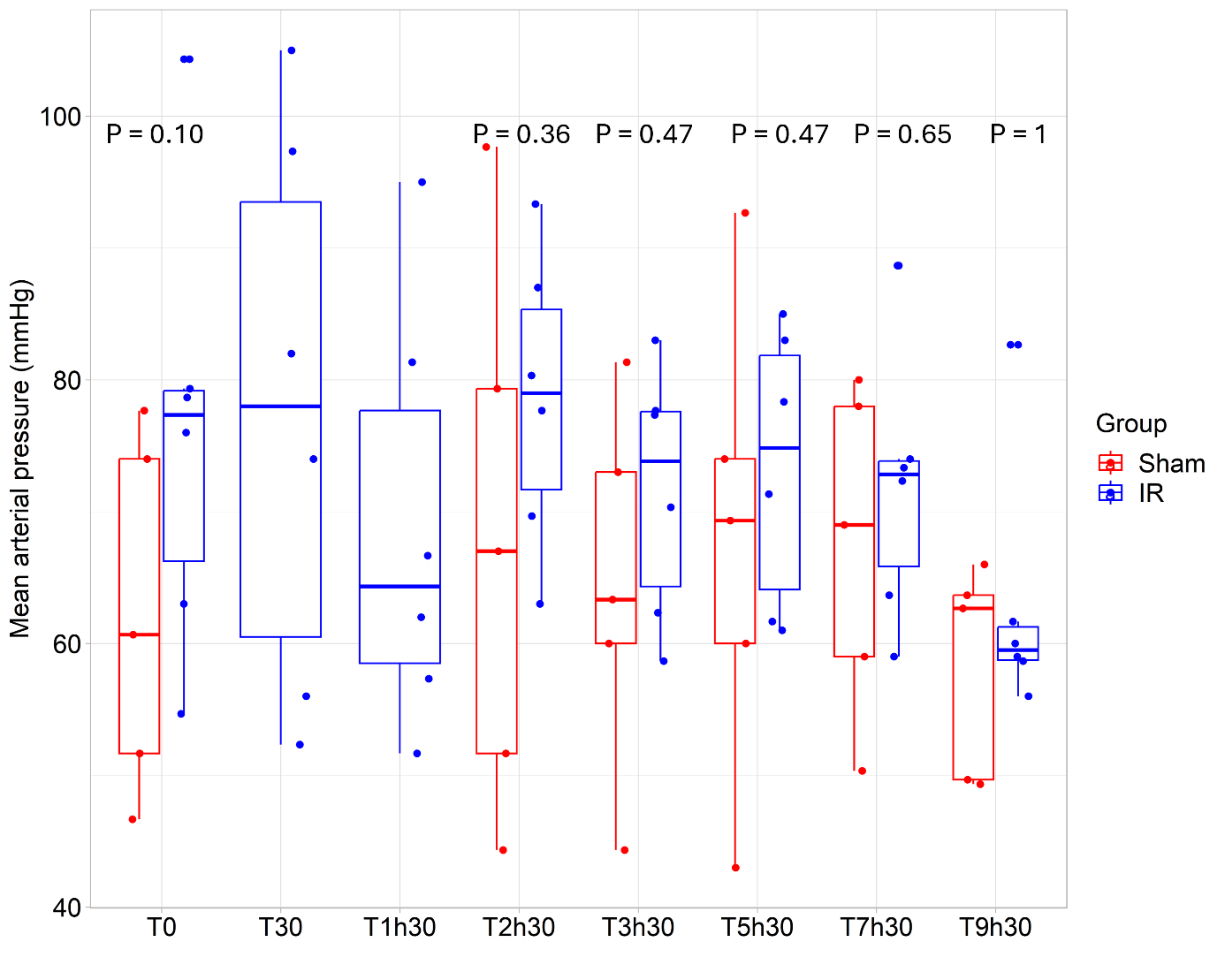
**

**Figure S9c – Comparison of pigs number which received noradrenaline**

**
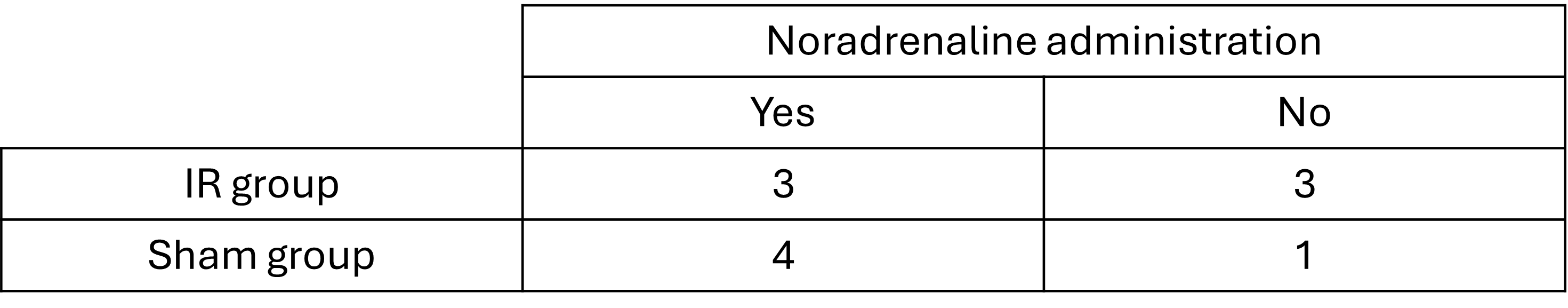
**

**Fisher test: p-value = 0.55**

**Figure S9d – Quantity of noradrenaline received for “IR” pigs and “Sham” pigs**

**
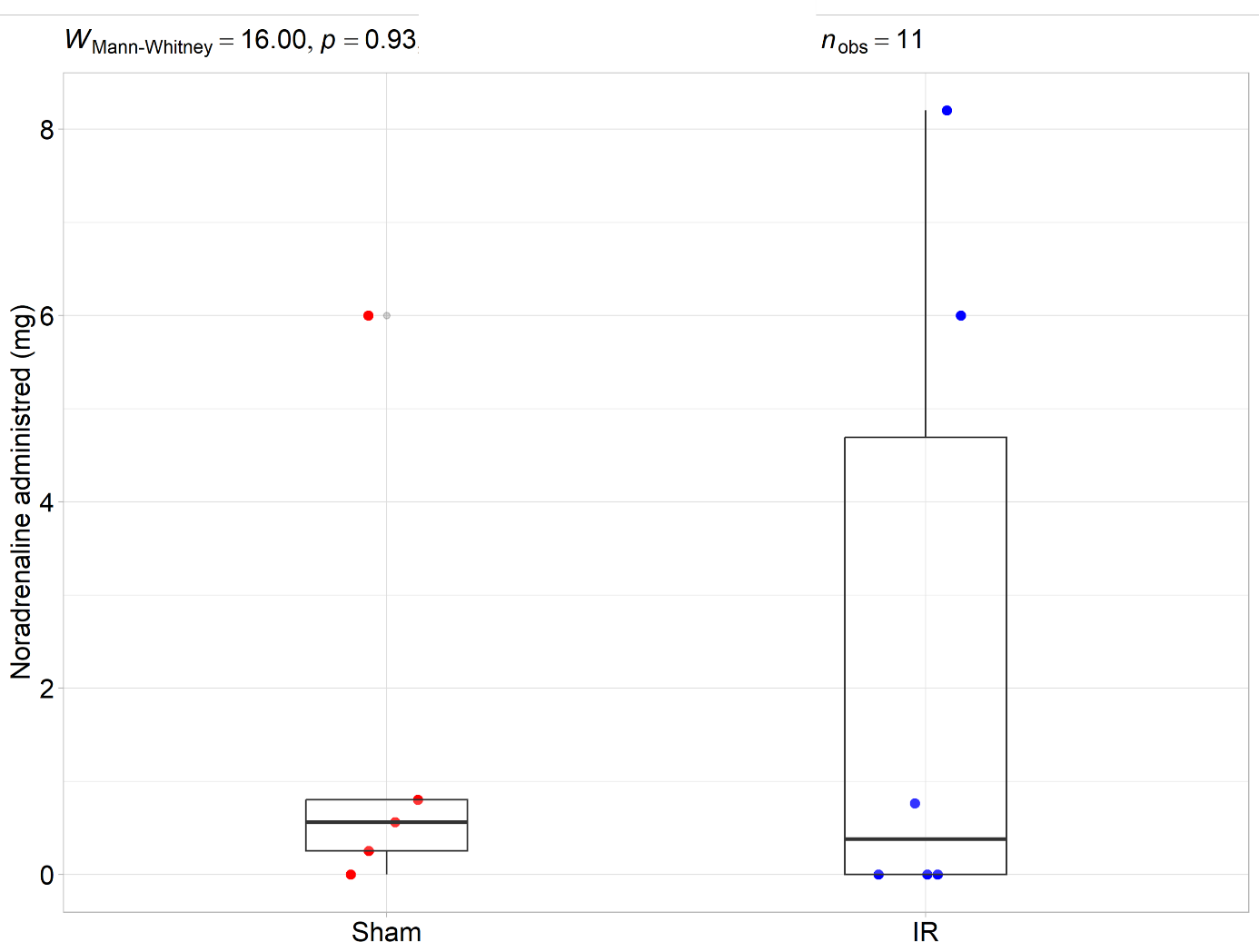
**

**Figure S9e – Plasma creatinine in the Sham group (N = 5). Plasma creatinine was compared between pigs receiving noradrenaline and pigs not receiving noradrenaline. Significance was assessed by linear mixed model.**

**
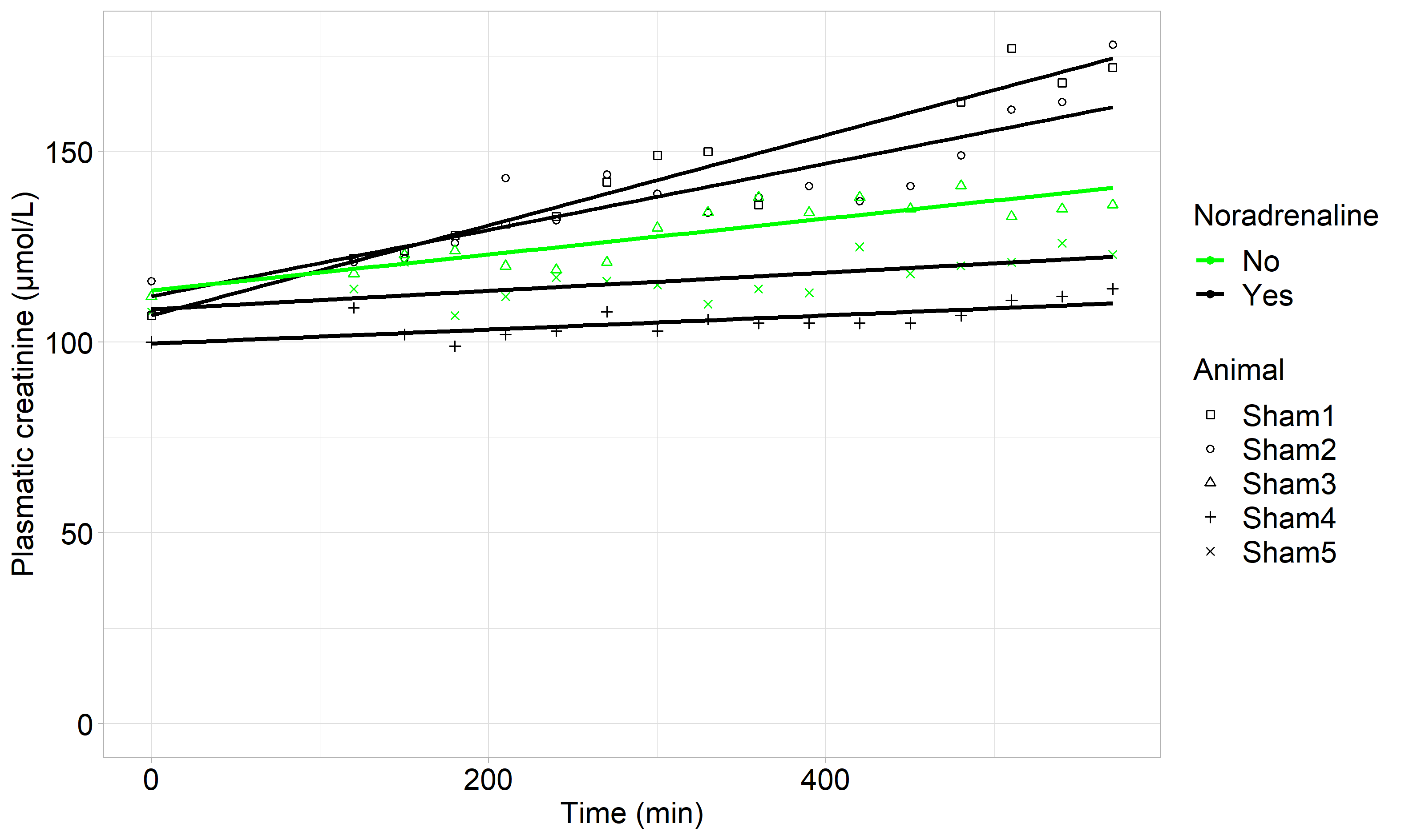
**

The table below shows the results of the models in Sham group. Models are expressed as:

Plasma creatinine ~ Noradrenaline + Time + Noradrenaline:Time + (1|Animal)`

Time, Noradrenaline and interaction between group and time were assimilated as a fixed effect. Animals were assimilated as a random effect.

| **Response variable** | **Noradrenaline** | **Estimate** | **CI95%** | **P** |
| --- | --- | --- | --- | --- |
| Plasma creatinine | No | + 0.047 µmol/L | 0.023 µmol/L, 0.072 µmol/L | < 0.0001 |
|  | Yes | + 0.060 µmol/L | 0.009 µmol/L, 0.112 µmol/L | 0.334 |

**Figure S9f – Plasma creatinine in the IR group (N = 6). Plasma creatinine was compared between pigs receiving noradrenaline and pigs not receiving noradrenaline. Significance was assessed by linear mixed model.**

**
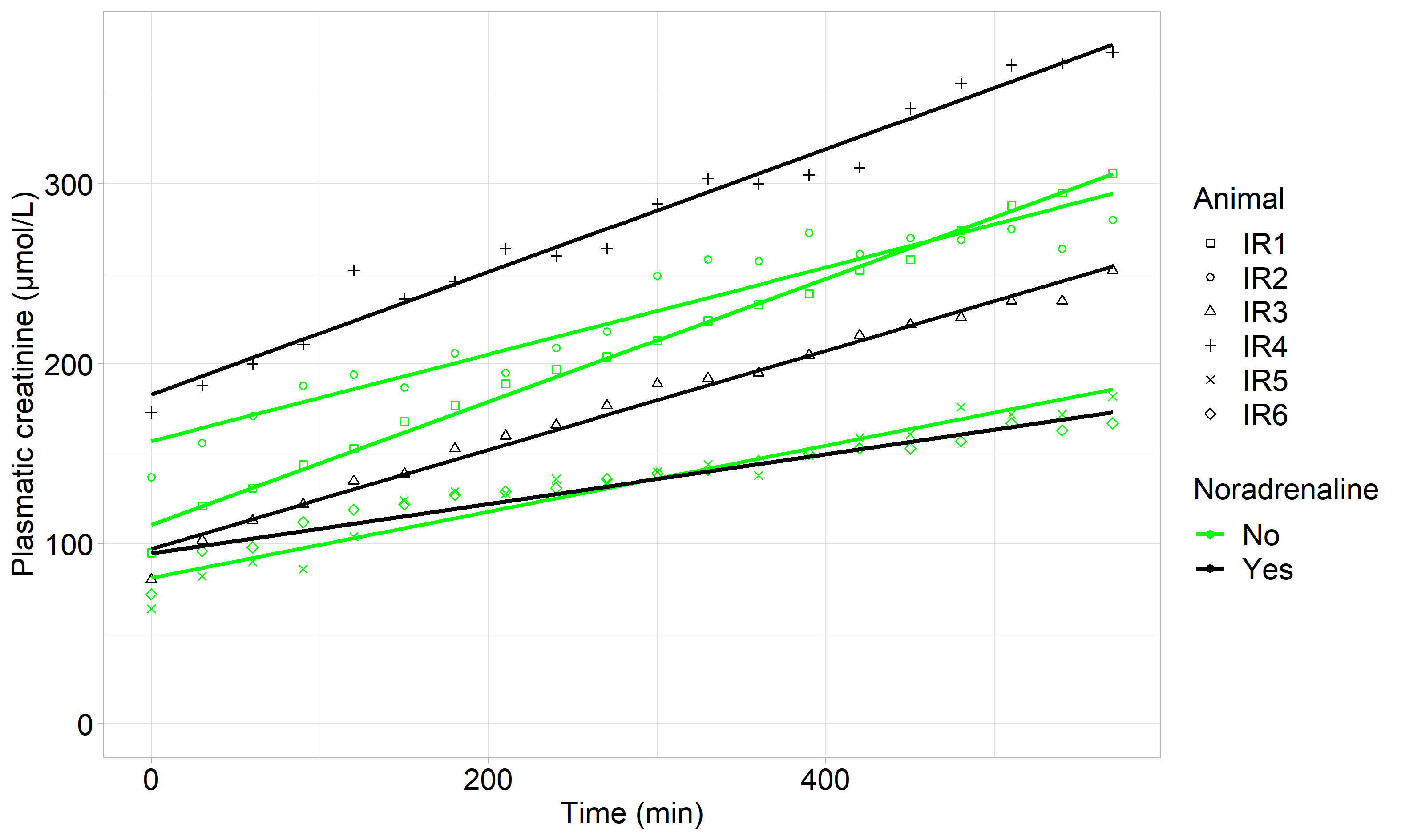
**

The table below shows the results of the models in IR group. Models are expressed as:

Plasma creatinine ~ Noradrenaline + Time + Noradrenaline:Time + (1|Animal)`

Time, Noradrenaline and interaction between group and time were assimilated as a fixed effect. Animals were assimilated as a random effect.

| **Response variable** | **Noradrenaline** | **Estimate** | **CI95%** | **P** |
| --- | --- | --- | --- | --- |
| Plasma creatinine | No | + 0.256 µmol/L | 0.232 µmol/L, 0.279 µmol/L | < 0.0001 |
|  | Yes | + 0.252 µmol/L | 0.195 µmol/L, 0.307 µmol/L | 0.79 |

**Figure S9g – Urinary NGAL in the sham group (N = 5). Urinary NGAL was compared between pigs receiving noradrenaline and pigs not receiving noradrenaline. At each timepoint, an analysis using Kruskall-Wallis test was performed**

**
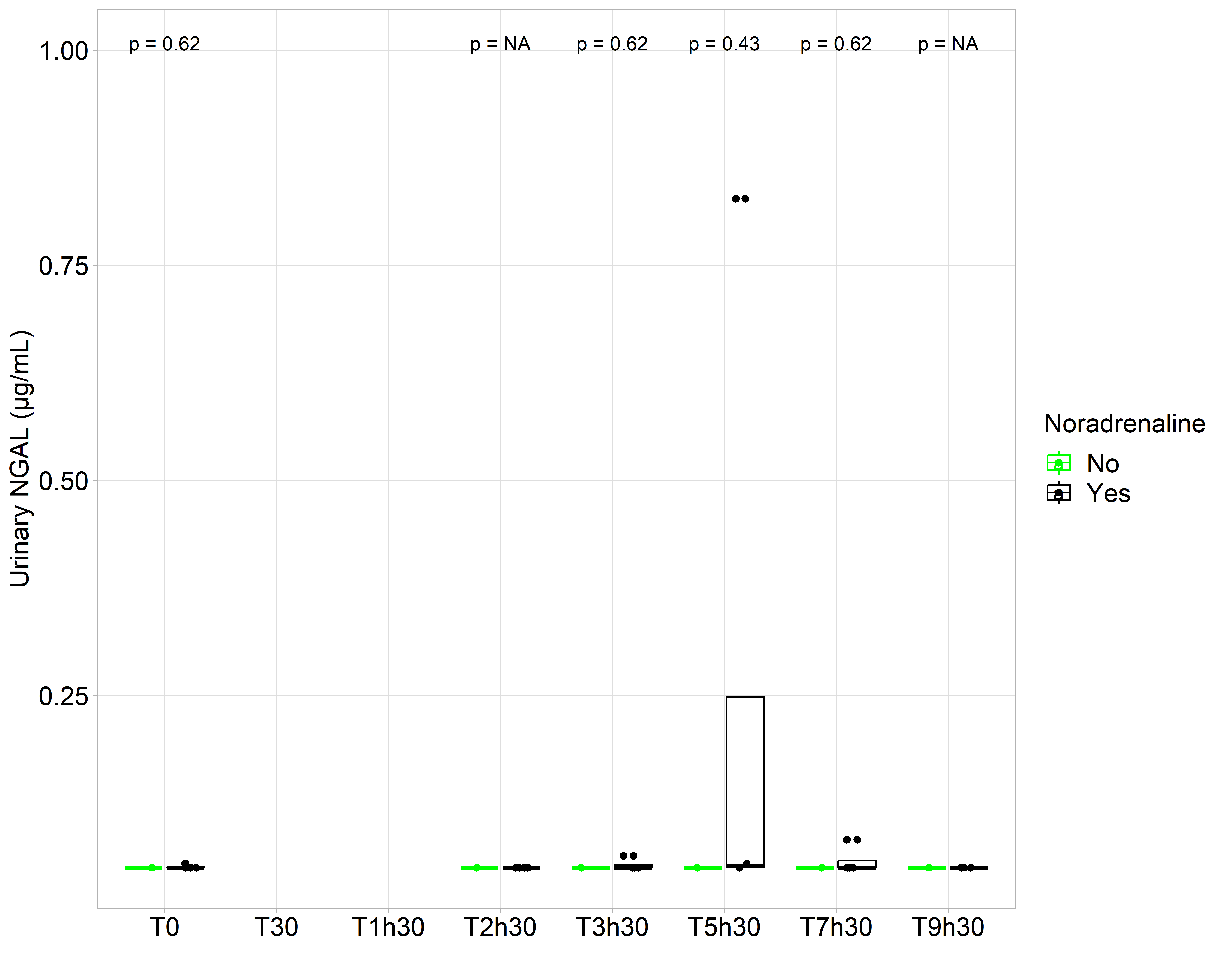
**

**Figure S9h – Urinary NGAL in the IR group (N = 6). Urinary NGAL was compared between pigs receiving noradrenaline and pigs not receiving noradrenaline. At each timepoint, an analysis using Kruskall-Wallis test was performed**

**
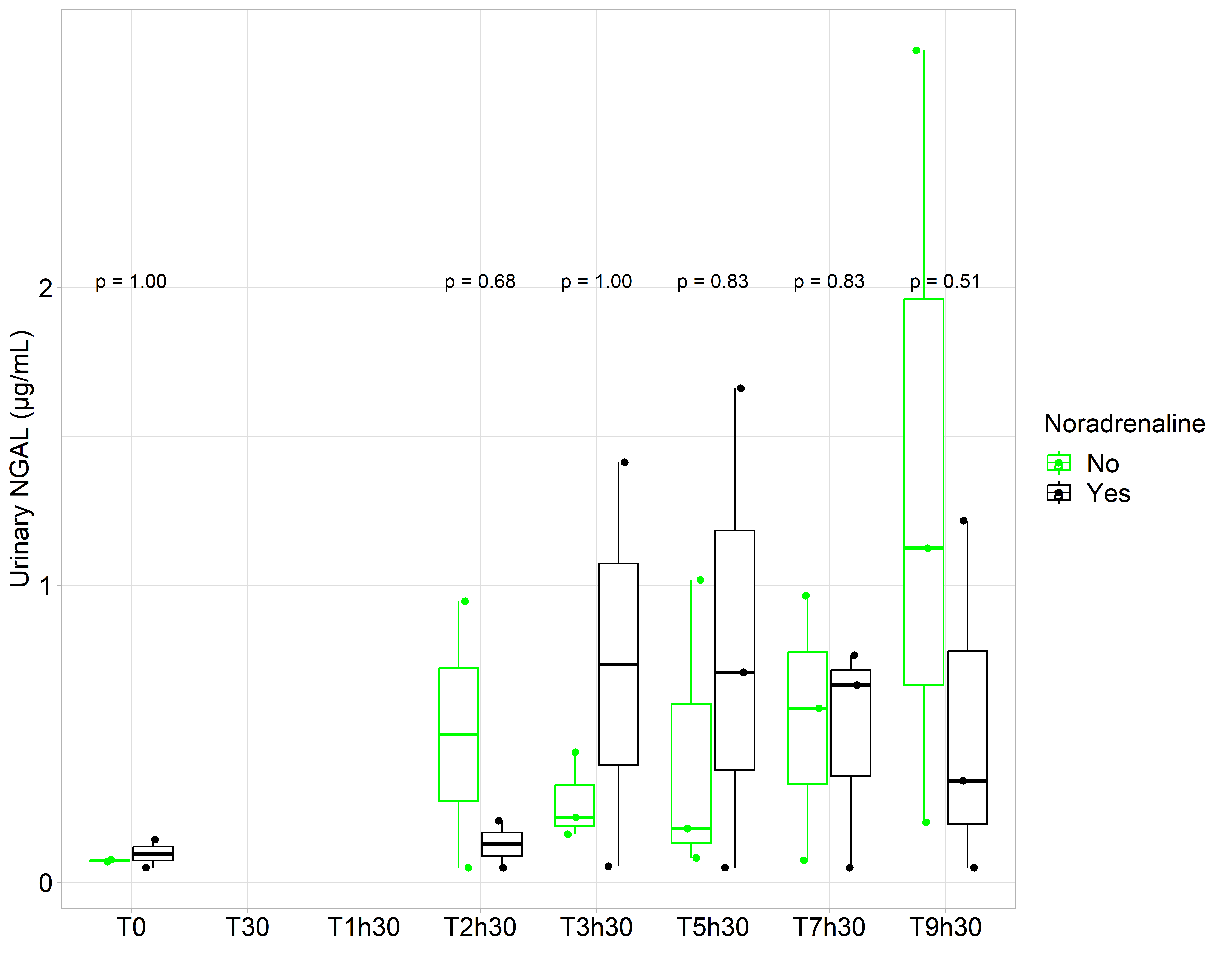
**

**Figure S10a – Urinary NGAL in the Sham group (N = 5). Urinary NGAL was compared between pigs receiving glucose and pigs not receiving glucose. At each timepoint, an analysis using Kruskall-Wallis test was performed**

**
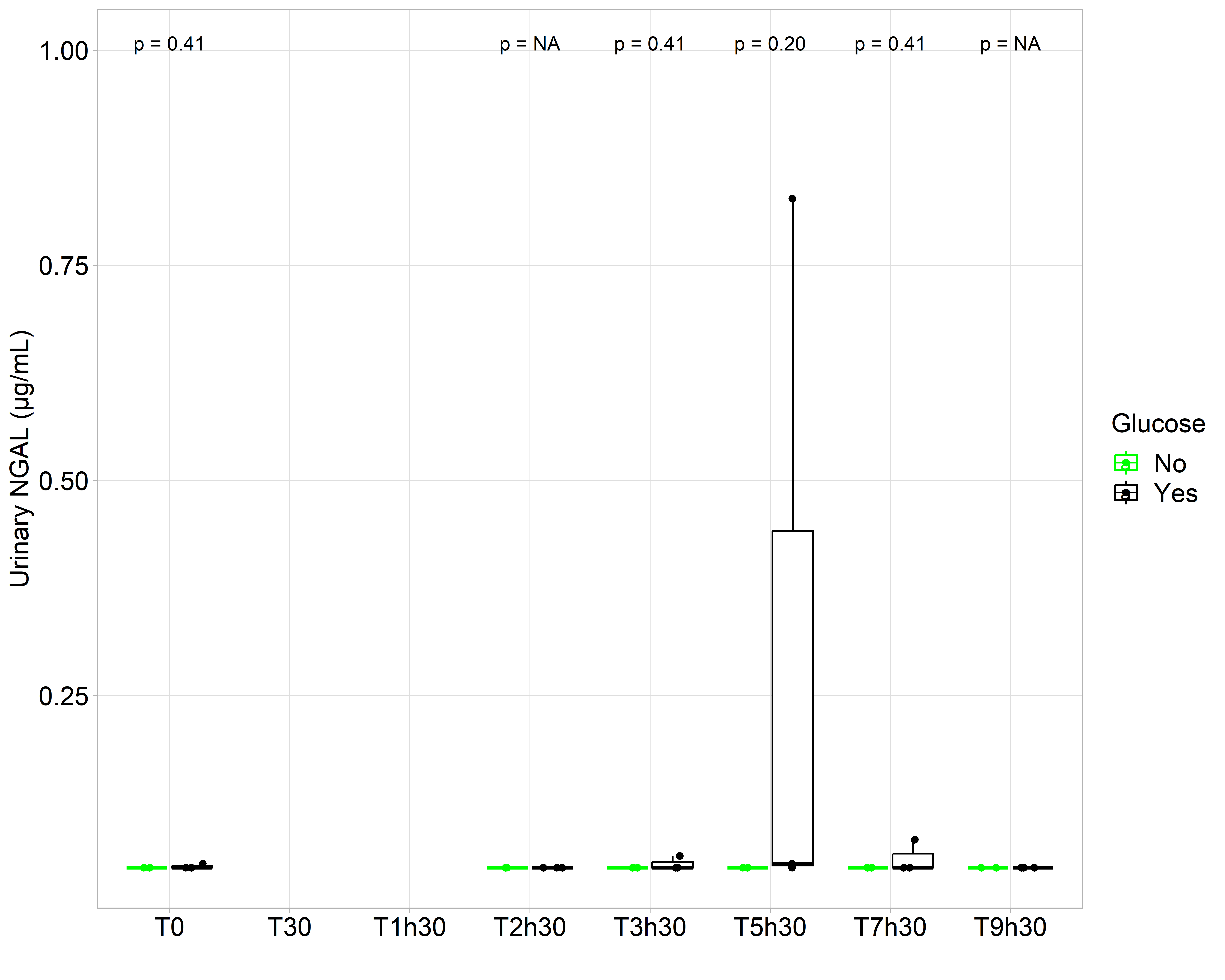
**

**Figure S10b – Urinary NGAL in the IR group (N = 6). Urinary NGAL was compared between pigs receiving glucose and pigs not receiving glucose. At each timepoint, an analysis using Kruskall-Wallis test was performed**

**
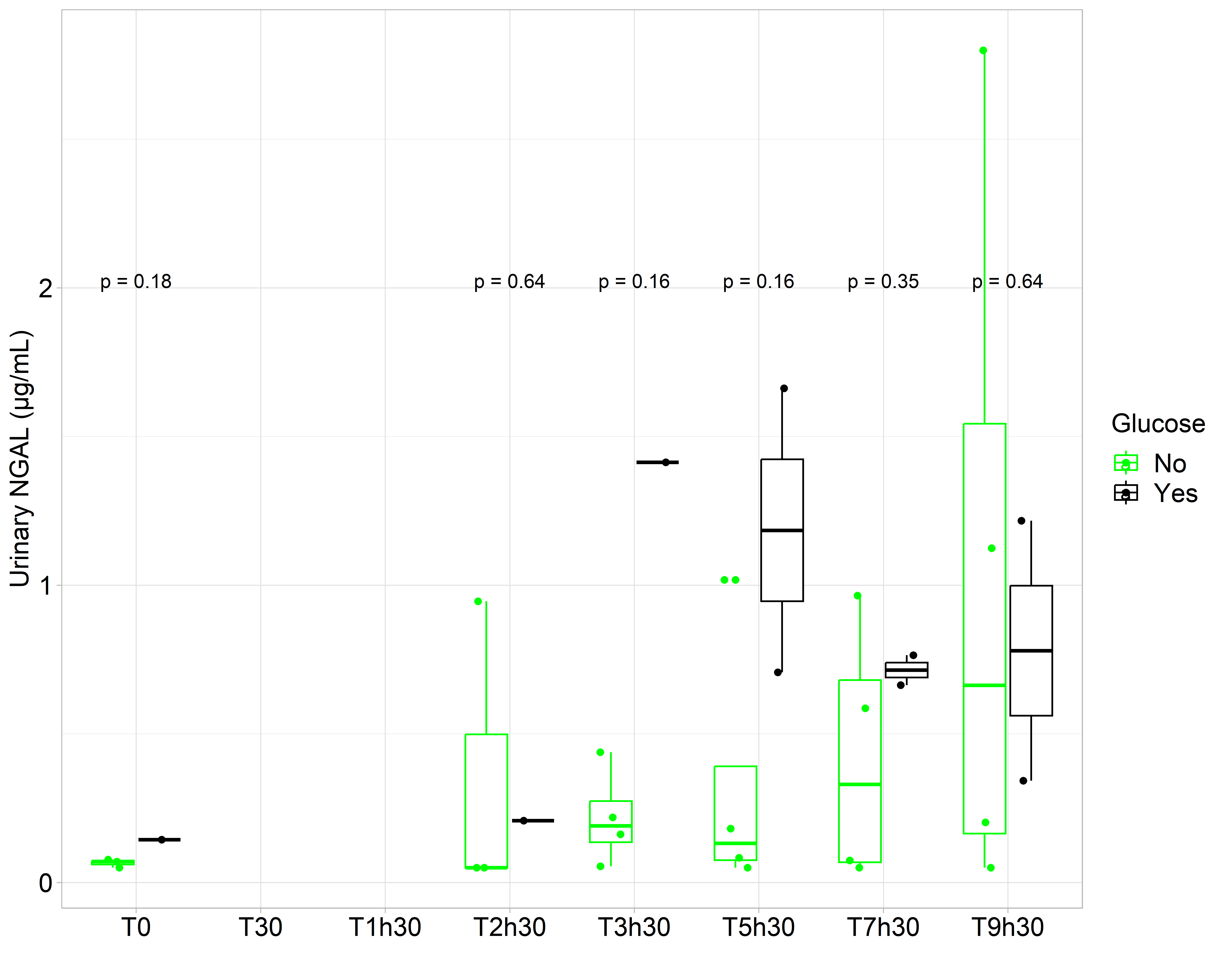
**

**Figure S10c – Plasma creatinine in the Sham group (N = 5). Plasma creatinine was compared between pigs receiving glucose and pigs not receiving glucose. Significance was assessed by linear mixed model.**

**
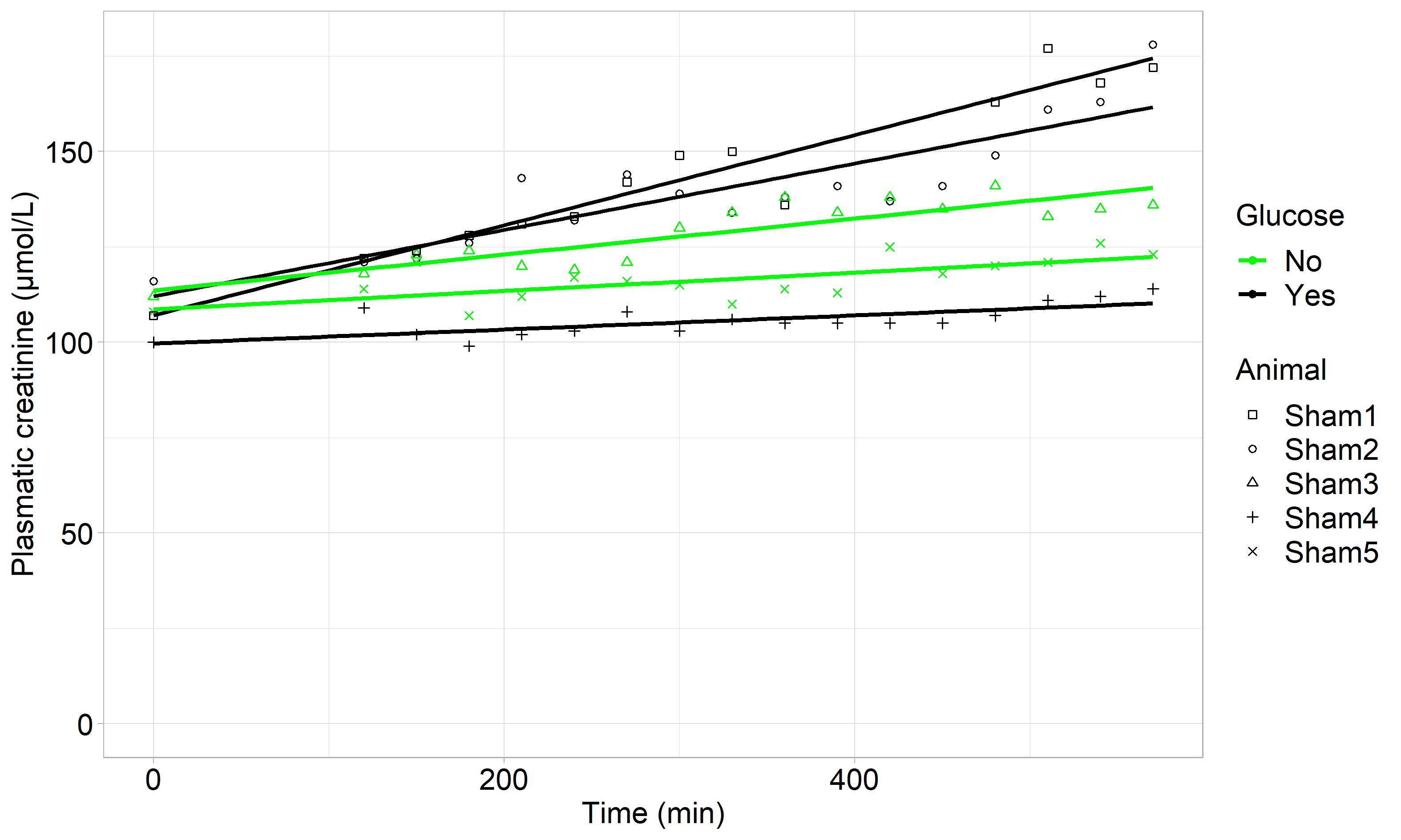
**

The table below shows the results of the models in Sham group. Models are expressed as:

Plasma creatinine ~ Glucose + Time + Glucose:Time + (1|Animal)`

Time, Glucose and interaction between group and time were assimilated as a fixed effect. Animals were assimilated as a random effect.

| **Response variable** | **Glucose** | **Estimate** | **CI95%** | **P** |
| --- | --- | --- | --- | --- |
| Plasma creatinine | No | + 0.036 µmol/L | 0.020 µmol/L, 0.052 µmol/L | < 0.0001 |
|  | Yes | + 0.073 µmol/L | 0.037 µmol/L, 0.110 µmol/L | 0.0006 |

**Figure S10d – Plasma creatinine in the IR group (N = 6). Plasma creatinine was compared between pigs receiving glucose and pigs not receiving glucose. Significance was assessed by linear mixed model.**

**
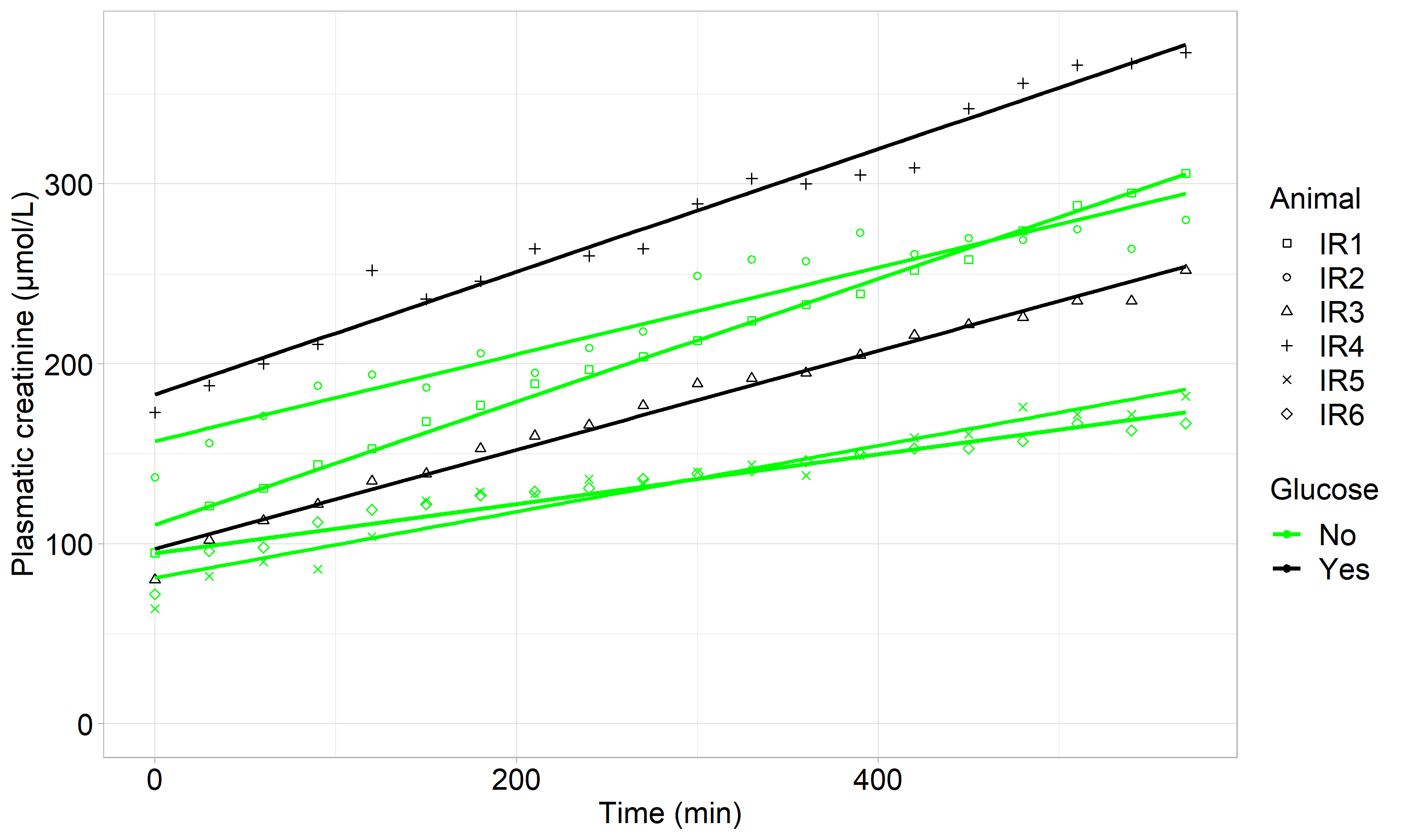
**

The table below shows the results of the models in IR group. Models are expressed as:

Plasma creatinine ~ Glucose + Time + Glucose:Time + (1|Animal)`

Time, Glucose and interaction between group and time were assimilated as a fixed effect. Animals were assimilated as a random effect.

| **Response variable** | **Glucose** | **Estimate** | **CI95%** | **P** |
| --- | --- | --- | --- | --- |
| Plasma creatinine | No | + 0.227 µmol/L | 0.208 µmol/L, 0.245 µmol/L | < 0.0001 |
|  | Yes | + 0.307 µmol/L | 0.256 µmol/L, 0.358 µmol/L | < 0.0001 |

**Figure S11 – Rectal temperature for both group (“IR” group (N = 6) and “Sham” group (N = 5)). At each timepoint, an analysis using Kruskall-Wallis test was performed**

**
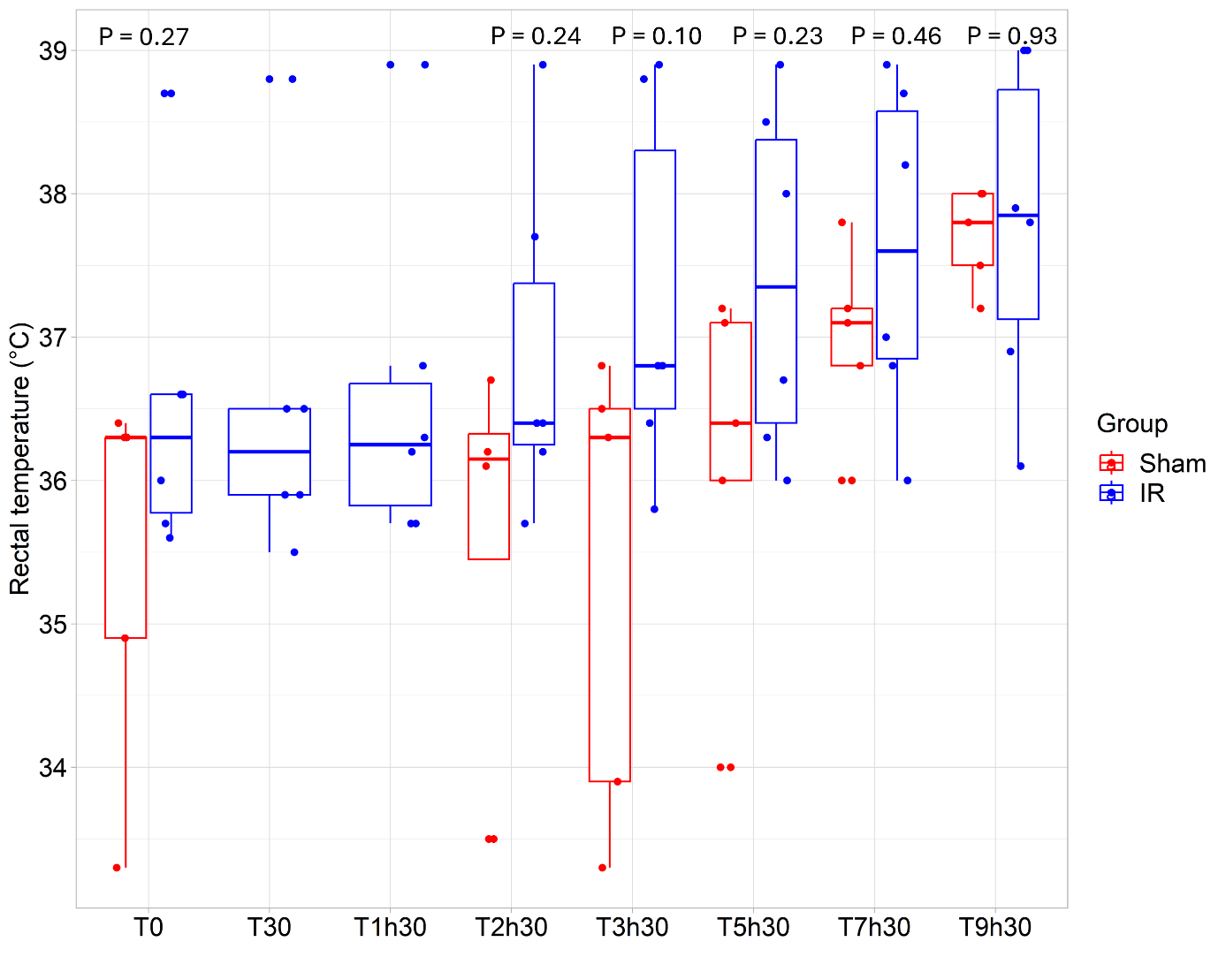
**
